# Metal–Ligand Proton Tautomerism, Electron Transfer, and C(sp^3^)–H Activation by a 4-Pyridinyl-Pincer Iridium Hydride Complex

**DOI:** 10.1021/jacs.3c03376

**Published:** 2023-08-08

**Authors:** Tariq M. Bhatti, Akshai Kumar, Ashish Parihar, Hellan K. Moncy, Thomas J. Emge, Kate M. Waldie, Faraj Hasanayn, Alan S. Goldman

**Affiliations:** † Department of Chemistry and Chemical Biology, Rutgers, The State University of New Jersey, Piscataway, New Jersey 08854, United States; ‡ Centre for Nanotechnology, Indian Institute of Technology Guwahati, Guwahati 781039, Assam, India; § Department of Chemistry, American University of Beirut, Beirut 1107 2020, Lebanon

## Abstract

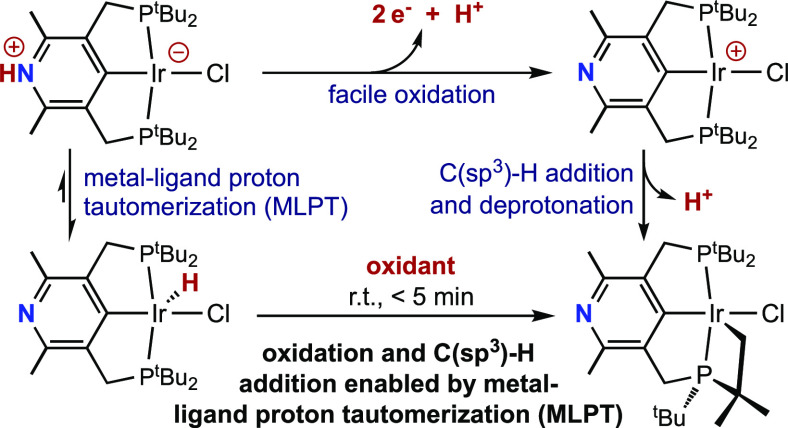

The *para*-N-pyridyl-based PCP pincer proligand 3,5-bis(di-*tert*-butylphosphinomethyl)-2,6-dimethylpyridine (pN-^tBu^PCP-H) was synthesized and metalated to give the iridium complex (pN-^tBu^PCP)IrHCl (**2-H**). In marked contrast with its phenyl-based congeners, e.g., (^tBu^PCP)IrHCl and derivatives, **2-H** is highly air-sensitive and reacts with oxidants such as ferrocenium, trityl cation, and benzoquinone. These oxidations ultimately lead to intramolecular activation of a phosphino-*t*-butyl C(sp^3^)–H bond and cyclometalation. Considering the greater electronegativity of N than C, **2-H** is expected to be less easily oxidized than simple PCP derivatives; cyclic voltammetry and DFT calculations support this expectation. However, **2-H** is calculated to undergo metal–ligand-proton tautomerism (MLPT) to give an N-protonated complex that can be described with resonance forms representing a zwitterionic complex (with a negative charge on Ir) and a *p*-N-pyridylidene (a remote N-heterocyclic carbene) Ir(I) complex. One-electron oxidation of this tautomer is calculated to be dramatically more favorable than direct oxidation of **2-H** (ΔΔ*G*° = −31.3 kcal/mol). The resulting Ir(II) oxidation product is easily deprotonated to give metalloradical **2**^•^ which is observed by NMR spectroscopy. **2**^•^ can be further oxidized to give cationic Ir(III) complex**, 2^+^**, which can oxidatively add a phosphino-*t*-butyl C–H bond and undergo deprotonation to give the observed cyclometalated product. DFT calculations indicate that less sterically hindered analogues of **2^+^** would preferentially undergo intermolecular addition of C(sp^3^)–H bonds, for example, of *n*-alkanes. The resulting iridium alkyl complexes could undergo facile β-H elimination to afford olefin, thereby completing a catalytic cycle for alkane dehydrogenation driven by one-electron oxidation and deprotonation, enabled by MLPT.

## 1 Introduction

**Scheme 1:**
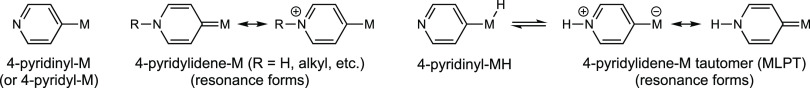
Pyridinyl and Pyridylidene Metal Complexes (Illustrated for Metalation at the 4-Position)

Organometallic pyridinyl and pyridylidene ligands (Scheme 1) are a rich platform for multifunctional and noninnocent reactivity. Pincer ligands are privileged scaffolds in organometallic chemistry, with a wide variety of catalytic applications. Both emerged in the 1970s,^1,2^ and while neither took inspiration from nature at the time, the discovery of a pyridylidene-pincer nickel cofactor in lactate racemase is a gratifying example of their merger in recent years.^3,4^

Pyridinyl and especially pyridylidene ligands can be viewed as a class of Fischer carbenes^2,5−12^ in which p(π) electrons of the pyridyl nitrogen and the backbonding d-electrons of the metal compete to populate a vacant p orbital on the ipso carbon.^13^ The remote nitrogen therefore strongly influences the electronics at the metal center, enabling electronic switchability and proton-responsiveness.^14,15^ Quaternizing the nitrogen results in a significant increase of positive charge at the metal center, including examples with “P(C-pyridinyl)P”-pincer complexes.^16,17^ Milstein and co-workers have studied P(C-pyridinyl)P-pincer ruthenium complexes as platforms for metal–ligand cooperative aromatization/dearomatization, enabling diverse reactivity including dihydrogen activation, alcohol dehydrogenation, and alcohol-amine dehydrogenative coupling.^18^

In the case of lactate racemase, mechanistic studies of the enzyme^19,20^ and synthetic model complexes^21−23^ have arrived at a proton-coupled hydride transfer mechanism^24^ for the racemization of lactate. Current evidence points to the transfer of hydride to and from the carbenic ipso carbon.

The influence of the metal upon pyridinyl ligands is demonstrated in the markedly enhanced basicity and nucleophilicity of 2-pyridinyl and 4-pyridinyl organometallic complexes compared with free pyridine,^25−28^ presumably a consequence of π-electron donation from the metal center. In the case of a metal hydride complex, this interaction would also be expected to increase its acidity. The combination of increased acidity of the hydride and high basicity at nitrogen raises the possibility of an interesting example of metal–ligand proton tautomerism (MLPT), a phenomenon that has achieved increasing recognition of late.^29−31^ In this case a metal hydride would interconvert with a lower oxidation state pyridylidene tautomer—alternatively formulated as a zwitterionic pyridyl complex with a formal negative charge on the metal (Scheme 1).

Beyond the pincer motif, there are a growing number of organometallic pyridinyl and pyridylidene complexes in the literature.^5,12^ However, there are no examples that include transition metal hydrides—or at least ones where the transition metal hydride is the dominant tautomer at equilibrium (examples of C–H activation alpha to a pyridyl nitrogen invariably prefer the pyridylidene tautomer at equilibrium^32−38^).

In the present study, we find that metal–ligand proton tautomerism of a 4-lutidinyl pincer iridium hydride strongly favors its reaction with one-electron oxidants. Initial oxidation, in turn, leads to secondary proton and electron transfer, affording an unusual and highly reactive four-coordinate cationic Ir(III) complex. Ultimately, an intramolecular C(sp^3^)–H activation results, driven by proton and electron transfer, having been initiated by MLPT.^39^

## 2 Experimental Results

### 2.1 Synthesis and Characterization of 2-H

**Figure 1:**
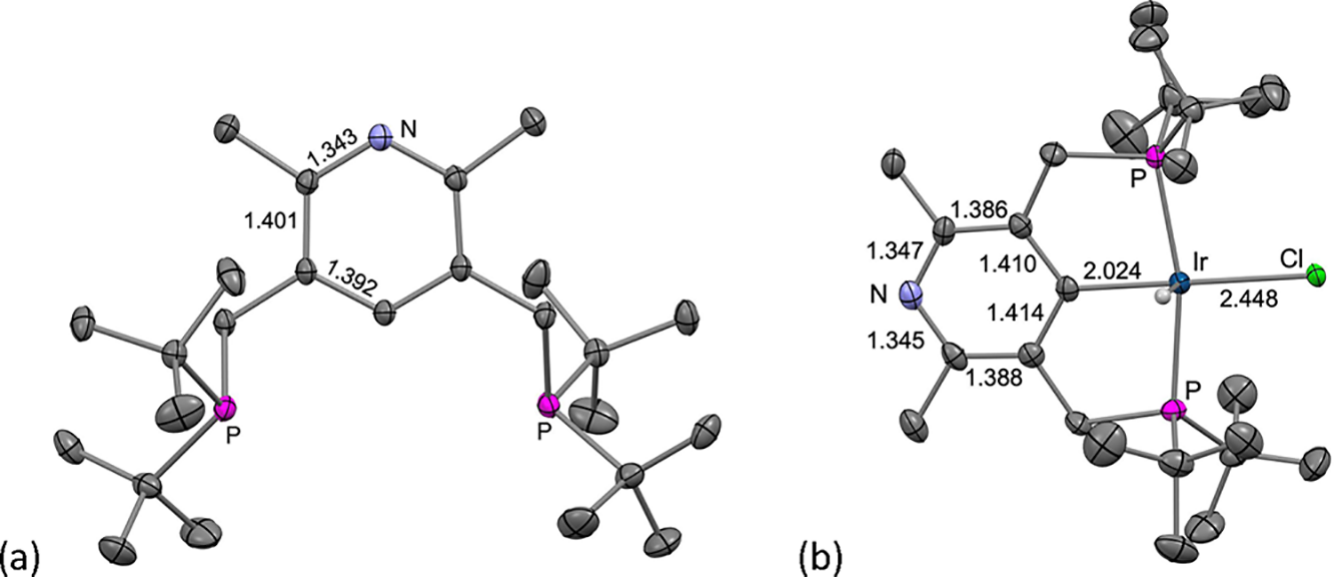
Molecular structures of (a) (pN-^tBu^PCP-H) (**1**) and (b) (pN-^tBu^PCP)IrHCl (**2-H**) determined by XRD. Hydrogen atoms, except for the hydride ligand, were omitted for clarity. Selected bond lengths in Å.

**Scheme 2:**
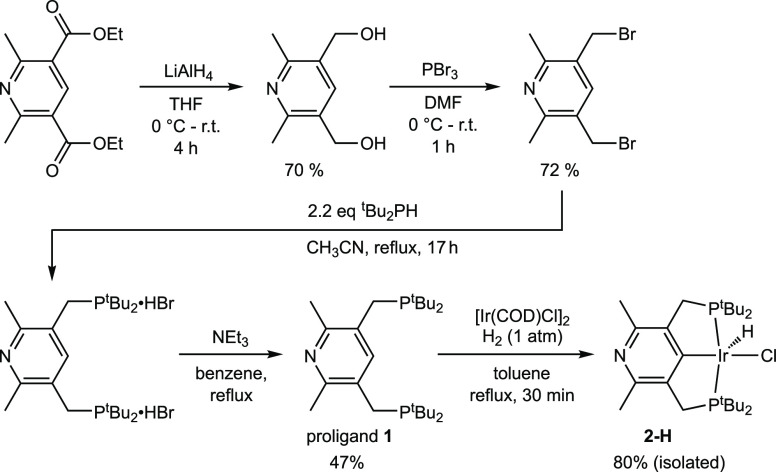
Synthesis of pN-^tBu^PCP-H (1) and Metalation to Give 2-H

The 4-lutidinyl “PCP” proligand, pN-^tBu^PCP-H (**1**), is prepared from the Hantzsch pyridine ethyl ester,^40^ as depicted in Scheme 2 and described in the Supporting Information. X-ray quality crystals of proligand **1** were grown by slow evaporation from benzene; the crystallographically determined molecular structure is shown in Figure 1a. Refluxing a mixture of **1** with [Ir(*COD*)Cl]_2_ (COD = 1,5-cycloctadiene) in toluene under 1 atm hydrogen resulted in a change of the solution color from orange to cherry red within a few minutes, with metalation apparently complete within 30 min. Evaporation of the solvent under vacuum afforded an orange, microcrystalline, air-sensitive powder, **2-H** (Scheme 2). ^1^H, ^13^C, and ^31^P NMR spectra of **2-H** are similar to those of (^tBu^PCP)IrHCl (**3-H**),^1,41^ (*p*-NO_2_-^tBu^PCP)IrHCl,^42^ (*p*-MeO-^tBu^PCP)IrHCl,^43^ and (*p*-Me_2_N-^tBu^PCP)IrHCl.^44^ In benzene-d_6_, a hydride resonance appears as a triplet at −41.9 ppm (^2^*J*_P–H_ = 12 Hz) in the ^1^H NMR spectrum. The phosphino-*t*-butyl groups as well as the methylene protons of the pincer arms are inequivalent in the ^1^H NMR spectrum; the *t*-butyl groups appear as two virtual triplets and the methylene protons appear as two doublets of virtual triplets. The selectively proton-decoupled ^31^P NMR spectrum shows a doublet at 68.8 ppm with a coupling to the hydride proton of ^2^*J*_P–H_ = 12 Hz. X-ray quality crystals of complex **2-H** were grown from pentane/toluene at −40 °C, and the XRD structure (Figure 1b) was consistent with the assignment based on NMR spectroscopy, although the hydride ligand was not unambiguously located.

### 2.2 Basicity of 2-H

**Scheme 3:**
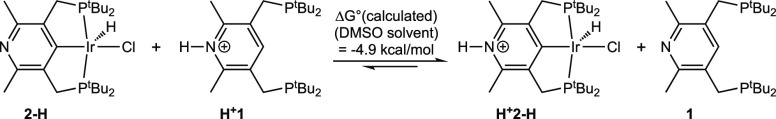
Calculated Equilibrium Illustrating Increased Basicity of **1** upon Metalation

To estimate the basicity of the remote nitrogen in complex **2-H**, a solution of 2,6-lutidinium chloride in DMSO (p*K*_a_ 4.46) was added to solid **2-H** (which is insoluble in DMSO). **2-H** was completely taken up into solution—indicating that the remote nitrogen was protonated by the 2,6-lutidinium salt. The resulting **2-H•HCl** is soluble in DMSO and water. However, when a solution of DBU•HCl in DMSO (p*K*_a_ = 13.9 in DMSO^45^) is added to solid **2**, only some of the materials is taken into the solution, suggesting that p*K*_a_ of **2-H•HCl** is perhaps similar to that of DBU•HCl. Determination of the protonation equilibrium is confounded, however, by the heterogeneous nature of this reaction as well as possible coordination of solvent or chloride trans to the hydride in **2-H•HCl.** Computationally, using density functional theory (see details below and Supporting Information), the pyridinyl nitrogen of **2-H** is predicted to be 3.6 p*K*_a_ units more basic (in DMSO) than the corresponding nitrogen of **1** (Scheme 3).

### 2.3 Oxidations of 2-H

Orange-red solutions of complex **2-H** in noncoordinating solvents immediately turn dark purple upon exposure to air. This attracted our interest because the analogous “parent” complex bearing a phenyl-based PCP ligand, (^tBu^PCP)IrHCl (**3-H**), and even analogues bearing π-donating para-substituents (methoxy^43^ or dimethylamino^44^) do not show similar air-sensitivity. We therefore investigated the oxidation of **2-H** with oxidants that generally afford simpler reactivity than O_2_. Importantly, and consistent with the pronounced difference in air-sensitivity between **2-H** and **3-H**, those oxidants described below that are found to react rapidly with **2-H** (Table 1) undergo no reaction with **3-H** under the same conditions in the timeframe of the investigation.

**Table 1:**
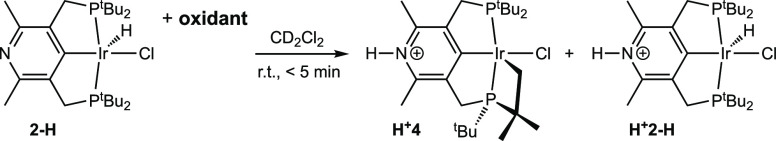
Reaction of 2-H with Various Oxidants

entry	[2-H]_0_ (mM)	oxidant	base	[H^+^4] (mM)	[H^+^2-H] (mM)
1	25	[Cp_2_Fe^+^][PF_6_^–^] (1 eq)		16.3	8.6
2	13.3	[Cp_2_Fe^+^][PF_6_^–^] (2 eq)		9.8	3.5
3	11.7	[Cp_2_Fe^+^][PF_6_^–^] (2 eq)	2,6-lutidine (9 eq)	10.6^a^	
4	12.5	[Ph_3_C^+^][B(C_6_F_5_)_4_^–^] (1 eq)		4.9	7.6
5	7	Benzoquinone (1 eq)^b^		1.9^a^^,^^d^	
6	7	Benzoquinone (1 eq)^c^		5.5^a^^,^^d^	
7	7.5	[(*p*-MeOC_6_H_4_)Ph_2_C^+^][BF_4_^–^] (2 eq)		*no reaction*	
8	11.1	[Cp*_2_Fe^+^][BF_4_^–^] (2 eq)^e^		*no reaction*	

a Remainder of mass balance is unreacted **2-H**.

b 40 min.

c 48 h.

d Product is complex **4**.

e Cp* = η^5^-C_5_Me_5_.

#### 2.3.1 Reaction of **2-H** with Cp_2_Fe^+^

**Scheme 4:**
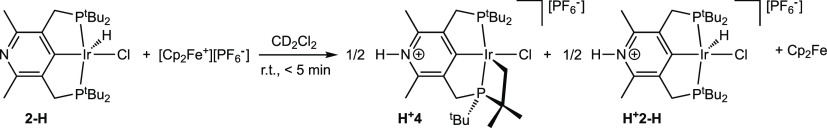
Reaction of **2-H** with 1 Equiv Ferrocenium Hexafluorophosphate

When 1 equivalent of ferrocenium hexafluorophosphate is added to a solution of **2-H** in dichloromethane-d_2_ the orange solution rapidly turns dark purple, followed by a slow conversion to light red. ^31^P and ^1^H NMR spectroscopy reveals that **2-H** has undergone quantitative conversion. The ^31^P{^1^H} NMR spectrum reveals the presence of two major species (Scheme 4).

One of the two major products observed is N-protonated, **2-H** (**H^+^2-H**; Scheme 4) which appears in the selectively decoupled ^31^P NMR spectrum as a doublet at 67.9 ppm, coupled (^2^*J*_P–H_ = 11.5 Hz) to a hydride at −40.5 ppm in the ^1^H NMR spectrum. The phosphino-*t*-butyl groups appear as two overlapping virtual triplets at 1.37 ppm. The second set of signals in the ^31^P NMR spectrum comprises two doublets, at 49.8 and 8.7 ppm, with a strong mutual coupling of 345 Hz. ^31^P–^1^H HMBC (see Supporting Information) was used to correlate the ^31^P NMR signal at 8.7 ppm with two doublets in the ^1^H NMR spectrum at 1.59 and 0.88 ppm, each with integral 3, as well as with two broad apparent triplets at 1.67 and 3.30 ppm, each with integral 1 (attributable to the two methyl groups and the two methylene protons, respectively, of the cyclometalated *t*-butyl group). The doublet at 49.8 ppm in the ^31^P NMR spectrum correlates with two doublets in the ^1^H NMR spectrum, each with integral 9, at 1.31 and 1.19 ppm. The presence of two overlapping signals at 10.66 and 10.57 ppm in a near 1:1 ratio in the ^1^H NMR spectrum indicated the new products are both protonated at the pyridinyl nitrogen. These spectral features are characteristic of a cyclometalated pincer complex,^43−50^ in which one of the *t*-butyl groups of **2-H** has undergone C–H activation to form **H^+^4** (Scheme 4). Thus, coupled with two one-electron oxidations and Ir–C bond formation, the hydride proton and a *t*-butyl proton of **2-H** have undergone net transfer to the pyridinyl nitrogen atoms of the now-cyclometalated complex **4** and another molecule of **2-H**.

**Scheme 5:**
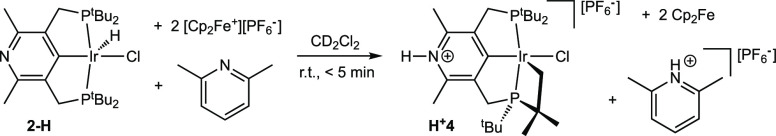
Reaction of **2-H** with 2 Equiv Ferrocenium Hexafluorophosphate and Lutidine

The addition of *two* equivalents ferrocenium hexafluorophosphate to a solution of **2-H** in dichloromethane-d_2_ also results in the orange solution rapidly turning dark purple followed by slow conversion to red, and ^31^P and ^1^H NMR spectroscopy again reveals that **2-H** has undergone quantitative conversion. However, it appears that, while in this case, there are enough oxidizing equivalents to convert all **2-H** to **H^+^4**, the protonation of all pN-pyridyl-PCP nitrogen upon reaction with one equiv. [Cp_2_Fe][PF_6_] (Scheme 4) inhibits further oxidation, and conversion to **H^+^4** is only 74%. Consistent with this hypothesis, in the presence of excess 2,6-lutidine (9 equiv), the reaction of two equiv. [Cp_2_Fe][PF_6_] with **2-H** results in nearly complete (90%) conversion to **H^+^4** upon mixing (Scheme 5).

#### 2.3.2 Reaction of **2-H** with Ph_3_C^+^

**Scheme 6:**
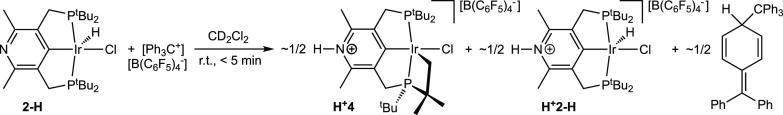
Reaction of **2** with 1 Equiv [Ph_3_C^+^][B(C_6_F_5_)_4_^–^]

Mixing complex **2-H** with 1 equivalent of [Ph_3_C^+^][B(C_6_F_5_)_4_^–^] (−0.11 V^51^ vs Fc/Fc^+^) in dichloromethane-d_2_ results in an instantaneous color change to dark purple. The solution then gradually lightens to orange. The ^1^H NMR spectrum reveals a 0.7:1 ratio of **H^+^4** to **H^+^2-H**, along with a half equivalent of Gomberg’s dimer ((Ph_3_C)_2_; Scheme 6). Thus, the trityl cation has acted as a single-electron oxidant, yielding approximately the same results as were obtained with 1 equivalent [Cp_2_Fe^+^][PF_6_].

#### 2.3.3 Reactions of **2-H** with Benzoquinone (BQ)

**Figure 2:**
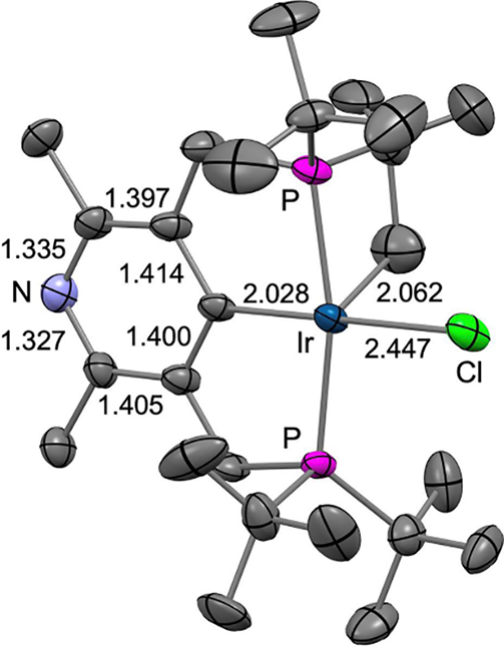
Molecular structure of **4** determined by XRD. H atoms omitted for clarity. Selected bond lengths in Å.

**Scheme 7:**
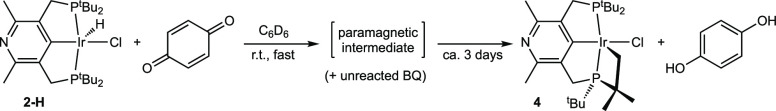
Reaction of **2** with 1 Equiv Benzoquinone

The reaction of complex **2-H** with one equivalent of BQ leads to quantitative conversion to **4** and hydroquinone within 48 h at 22 °C (Scheme 7). Hydroquinone precipitates as a white solid from the reaction mixture. Following filtration, slow evaporation of the filtrate at ambient temperature produced crystals of complex **4**, the molecular structure of which was determined by XRD (Figure 2).

A paramagnetic intermediate is observed in the course of the reaction of **2-H** with BQ. Upon addition of **2-H** (7 mM) to BQ (540 mM) in benzene-d_6_, the solution turns dark purple within 2 min of mixing. NMR reveals that approximately 50% of **2-H** has been converted to this paramagnetic species which has broad signals at −31 and 14.8 ppm in the ^1^H NMR spectrum that integrates in a 1:6 ratio. This ratio allows assignment of the signals to the α-methyl groups and *t*-butyl groups, respectively. It follows then that this intermediate contains two mirror planes of symmetry, which renders all four *t*-butyl groups as well as the two α-methyl groups equivalent on the relevant NMR timescale. The methylene signals of the pincer arms in this paramagnetic intermediate were not observed and may be broadened into the baseline. These signals, assigned to Ir(II) complex **2•**, diminish with concomitant growth of signals attributable to **4** over the next 2 h (see Supporting Information).

#### 2.3.4 Chemical Characterization of the Paramagnetic Intermediate Resulting from the Initial Single-Electron Oxidations

**Scheme 8:**
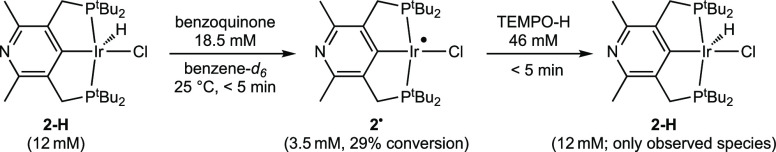
Reaction of **2^•^**, Generated In Situ, with TEMPO-H, to Give **2-H**

In an effort to further characterize the paramagnetic intermediate, a solution of **2-H** (12 mM) in benzene-d_6_ was allowed to react with only a slight excess (1.5 eq) of benzoquinone for 5 min at 25 °C; 29% conversion of **2-H** to the paramagnetic intermediate is observed by ^1^H NMR (Scheme 8). A 4-fold excess of TEMPO-H (1-hydroxy-2,2,6,6-tetramethyl-piperidine)(O-H BDFE = 65.2 kcal/mol^52^) was then added to quench the paramagnetic species. The ^1^H NMR spectrum taken immediately afterward indicated that **2-H** was the sole remaining iridium-containing species in the solution (Scheme 8). On the basis of these data, the paramagnetic species **2^•^** is assigned as the four-coordinate product resulting from the loss of H from **2-H**.

**Scheme 9:**
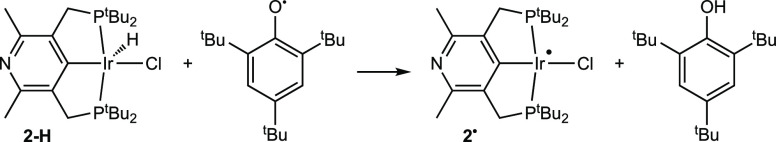
Reaction of **2-H** with 2,4,6-^t^Bu_3_C_6_H_3_O^**•**^

In accord with our assignment of **2**^•^, it was independently generated by the reaction of **2-H** with the isolable tri-t-butylphenoxy radical^52−54^ (BDFE = 76.7 kcal/mol^52,53^) (20% yield and conversion by ^1^H NMR; Scheme 9).

## 3 Mechanistic Considerations and DFT Calculations

### 3.1 Initial Oxidation of 2-H via MLPT

Based on the greater electronegativity of N than C, simple one-electron oxidation of **2-H** is expected to be thermodynamically less favorable than oxidation of phenyl-based (^tBu^PCP)IrHCl (**3-H**) or its 3,5-dimethylphenyl-based derivative **3’-H** (which is even more closely analogous to **2-H**). The results of DFT calculations are consistent with this simple reasoning: the ionization energy of **2-H** is calculated to be 5.2 kcal/mol less favorable than that of **3′-H**. In absolute terms, one-electron oxidation of **2-H** by Cp_2_Fe^+^ in CH_2_Cl_2_ is calculated to be endergonic by 14.0 kcal/mol. These values are in very good agreement with the results of cyclic voltammetry studies. The CV of **3-H** shows a chemically irreversible oxidation wave at 0.33 V vs Fc^0/+^, while that of **2-H** has a chemically irreversible wave at 0.59 V vs Fc^0/+^ (Figure S40) which implies that direct oxidation of **2-H** is 6.0 kcal/mol less favorable.

**Figure 3:**
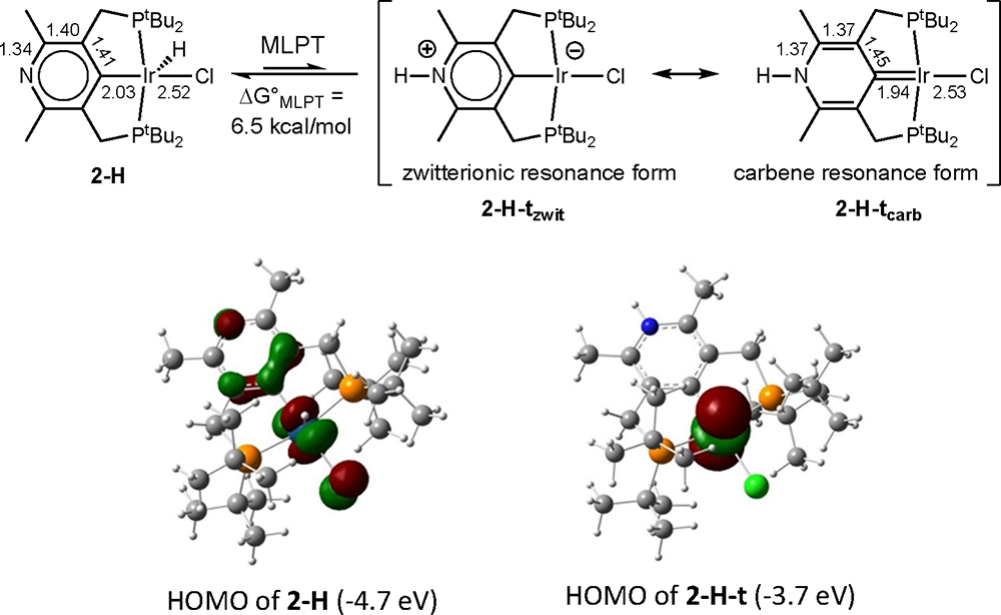
DFT-calculated structures and HOMOs of **2-H** and **2-H-t**. Bond lengths in Å.

We calculate that **2-H** can undergo transfer of H from Ir to the pyridinyl nitrogen; the resulting MLPT tautomer, **2-H-t**, is 6.5 kcal/mol higher in free energy than **2-H**. As shown in Figure 3 (top), there are two limiting resonance structures for the tautomer **2-H-t**, one representing a zwitterionic form (**2-H-t_zwit_**) and one a carbene form (**2-H-t_carb_**). A computed significant contraction of the Ir–C bond length (1.94 Å vs 2.03 Å for **2-H**), and a more pronounced alternation of interatomic distance in the heterocyclic ring of **2-H-t** compared with that of **2-H**, implicates a significant contribution from resonance form **2-H-t_carb_** (Figure 3).

**Figure 4:**
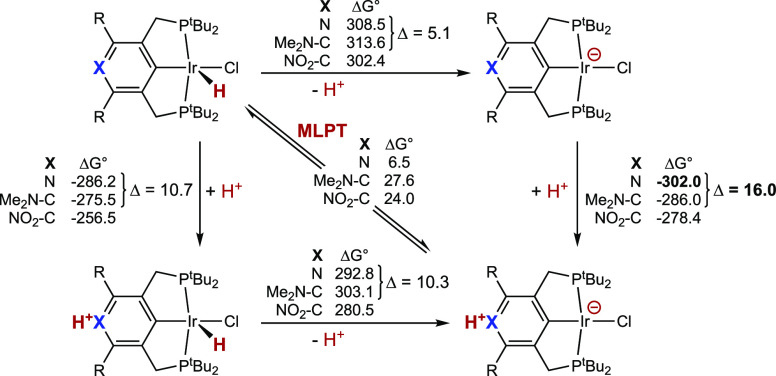
Thermochemical square scheme for MLPT calculated for **2-H** (R = Me) and (*p*-X-^tBu^PCP)IrHCl (X = Me_2_N and NO_2_; R = H). Calculated Gibbs free energies at 298 K and 1 atm in kcal/mol, M06L-D3 in CH_2_Cl_2_ continuum. Differences (Δ) between **2-H** (R = Me) and (*p*-X-^tBu^PCP)IrHCl (X = Me_2_N), discussed in the text, specifically indicated.

The low calculated free energy of tautomerization of **2-H**, Δ*G*°_MLPT_ = 6.5 kcal/mol, is attributable to the relatively high acidity of the **2-H** hydride, a result of the electron-withdrawing nature of the pyridinyl group, combined with the basicity of the pyridinyl nitrogen. The former effect is manifest by the calculated free energy of deprotonation of **2-H**, 308.5 kcal/mol, which, for comparison, is 3.9 kcal/mol less than that of **3′-H** and 5.1 kcal/mol less than that of (*p*-Me_2_N-PCP)IrHCl. The high basicity of the pyridinyl nitrogen is reflected in its proton affinity being 10.7 kcal/mol greater than that of the dimethylamino group of (*p*-Me_2_N-PCP)IrHCl. The pyridinyl N thus plays an unusual dual role of a group that is both quite basic and electron-withdrawing. The difference in Δ*G*°_MLPT_ between **2-H** and (*p*-Me_2_N-PCP)IrHCl (21.1 kcal/mol), however, is greater than even the sum of these two individual components (15.8 kcal/mol) would imply. This may be attributed to π-conjugation in tautomer **2-H-t** being more extensive than in **2-H**, as is suggested by the carbene resonance form **2-H-t_carb_**. Accordingly, protonation of the N site of **2^–^** is 16.0 kcal/mol, more exergonic than that of (*p*-Me_2_N-PCP)IrCl^**–**^ (compared with a difference of 10.7 kcal/mol for N-protonation of the respective neutral hydride complexes). These differences, and comparisons with (*p-*NO_2_-PCP)IrHCl, a (pincer)IrHCl complex with a strongly electron-withdrawing substituent, are illustrated in Figure 4.^55^

**Figure 5:**
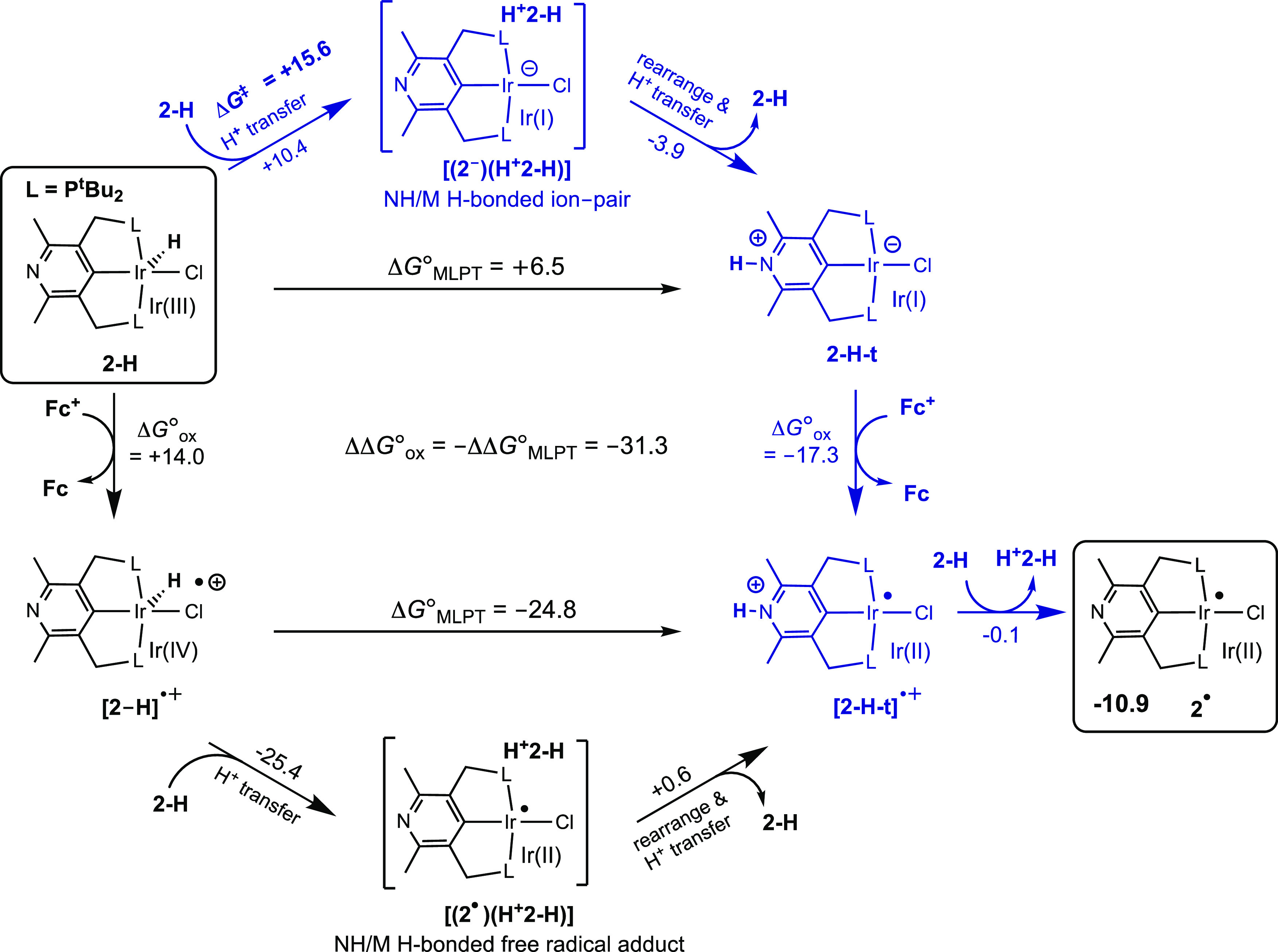
Thermochemical square scheme for the oxidation of **2-H** by Cp_2_Fe^+^ (**Fc^+^**) to give **[2-H-t]^•+^**, via direct oxidation of **2-H** or via tautomer **2-H-t**, and subsequent deprotonation by another molecule of **2-H** to give **2^•^**. Possible intermolecular pathways illustrated for MLPT steps, occurring either prior to or after oxidation, with favored (MLPT first) pathway indicated in blue. Calculated Gibbs free energies at 298 K and 1 M in kcal/mol; M06L-D3 in CH_2_Cl_2_ continuum.

One plausible pathway for the proposed MLPT would be via initial intermolecular proton transfer of the Ir–H proton of **2-H** to the nitrogen of a second **2-H** molecule. The barrier for this step is computed to be quite low (15.6 kcal/mol, Figure 5). Proton transfer initially gives an ion pair that can rearrange to deliver the proton to the nitrogen site of the deprotonated molecule of **2-H**, thereby affording **2-H-t.**

While tautomerization of **2-H** to give **2-H-t** is endergonic by only 6.5 kcal/mol, the oxidation of **2-H-t** by Cp_2_Fe^+^ is calculated to be very favorable (Δ*G*°_ox_ = −17.3 kcal/mol). Thus, MLPT of **2-H** favors oxidation by a remarkable extent of 31.3 kcal/mol (ΔΔ*G*°_ox_, Figure 5). For purposes of qualitatively understanding this effect, it is perhaps convenient to consider the zwitterionic resonance form **2-H-t_zwit_**. The parent complex **2-H** has a d^6^ Ir(III) center and a HOMO derived largely from the metal 5d_xz_ orbital (Figure 3). MLPT to give **2-H-t_zwit_** creates a square planar complex with a d^8^ Ir(I) center having a formally negative charge and a 5d_z_2 HOMO that is 1.0 eV higher than the HOMO in **2-H** (Figure 3). The reductive nature of MLPT with respect to the metal thereby explains the greatly more favorable oxidation of **2-H-t** relative to **2-H.**

Note that the difference in the oxidation energies of **2-H** and **2-H-t** (ΔΔ*G*°_ox_ = −31.3 kcal/mol) is necessarily equal to the difference in the tautomerization energies of **2-H** and its oxidized form [**2-H]^•+^** (−ΔΔ*G*°_MLPT_). Thus, in contrast with the parent complex **2-H**, MLPT of the radical cation **[2-H]^•+^** is highly exergonic (Δ*G*°_MLPT_ = −24.8 kcal/mol; Figure 5). This may be viewed in terms of the increased metal oxidation state and the increased electric charge greatly increasing the acidity of the IrH bond in **[2-H]^•+^** relative to **2-H** (with an expected decrease of at least 15 p*K*_a_ units^56,57^), while having a much smaller effect on the basicity of the nitrogen which is distant from the center of oxidation. Thus, upon oxidation, the nitrogen becomes the favored site for the proton.

For a given molecule, Δ*G*°_MLPT_ is equal to the difference between the Ir–H and N–H BDFEs. Similar to the acidity, the N–H BDFE in **2-H-t** is not expected to greatly change upon oxidation. Thus, the large change in Δ*G*°_MLPT_ implies that the BDFE of the Ir–H bond in **2-H** is greatly weakened upon oxidation.

Oxidation of **2-H-t** generates the free radical cation **2-H-t^•+^**. Subsequent deprotonation of **2-H-t^•+^** by an unreacted molecule of **2-H** is computed to be essentially ergoneutral (Δ*G*° = −0.1 kcal/mol), yielding the d^7^ Ir(II) complex **2^•^**, our assignment for the paramagnetic intermediate observed in the reaction with benzoquinone (Schemes 7 and 8). The net energy of the formation of **2^•^** and **H^+^2-H** from two molecules of **2-H** and ferrocenium is computed to be quite negative, Δ*G*° = −10.9 kcal/mol. MLPT thereby enables a facile path for net “H-abstraction” from a metal hydride, **2-H**, via one-electron oxidation. The calculations in the next section show that oxidation of **2^•^** by another ferrocenium is viable and that it triggers intramolecular C–H activation.

### 3.2 Oxidation of 2^•^ and Intramolecular C–H Activation

Oxidation of the Ir(II) complex **2^•^** by a second equivalent of ferrocenium is calculated to be only slightly endergonic (Δ*G*° = 7.9 kcal/mol). We calculated two minima, with nearly identical energies, for the resulting four-coordinate d^6^ cation. One of these has a square planar geometry with a triplet spin state (^3^**2^+^**), while the other has a seesaw geometry with a closed shell singlet state (**2^+^_bent_**). **2^+^_bent_** features an agostic C–H bond from a phosphino-*t*-butyl group donating into the otherwise empty orbital of the metal, with Ir–H and Ir–C bond distances of 2.08 and 2.81 Å.^58^ For the triplet state, no minimum with an agostic bond could be located which is not surprising given that this state has no empty metal d atomic orbital.

**Figure 6:**
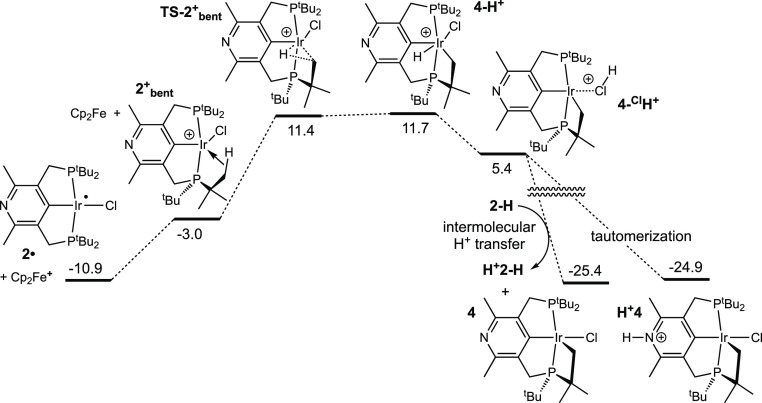
Oxidation of **2•** by Cp_2_Fe^+^, and subsequent cyclometalation and deprotonation to give **4**; calculated Gibbs free energies in kcal/mol normalized relative to two **2-H** and Cp_2_Fe^+^ (Figure 5); M06L-D3 in CH_2_Cl_2_ continuum.

Oxidative cleavage of the agostic C–H bond of **2^+^_bent_** is computed to proceed readily (Δ*G*^‡^ = 14.4 kcal/mol). The transition state^59^**TS-2^+^_bent_** has a geometry characterized by a nearly (but not quite) complete C–H bond cleavage (d = 1.65 Å) and large degree of Ir–H (1.56 Å) and Ir–C (2.22 Å) bond formation. **TS-2^+^_bent_** leads to the six-coordinate cyclometalated species **4-H^+^** which is calculated to be extremely acidic. Proton transfer from **4-H^+^** to the pyridinyl nitrogen of a second, neutral, molecule of **2-H**, to afford the observed metallacycle product **4** is highly exergonic (Δ*G*° = −36.6 kcal/mol). However, the proton in **4-H^+^** is in a sterically very encumbered environment and may be kinetically inaccessible for direct transfer to a pyridinyl nitrogen. A proton shift from the iridium center in **4-H^+^** to a phosphino group (bridging an Ir–P bond^60−62^) is exergonic by 4.3 kcal/mol, and the proton on the phosphine in **4-^P^H^+^** should be more accessible for transfer to **2-H**. Similarly, the shift of a proton from the iridium center in **4-H^+^** to the chloride ligand (Figure 6) is also exergonic, by 6.3 kcal/mol, leading to (**4-^Cl^H**^+^) which may also be viewed as an unsaturated cationic species with a coordinated HCl molecule. This species could readily transfer a proton to the basic nitrogen site of a molecule of **2-H**, either directly or perhaps by initial dissociation of free HCl. Attempts to locate TSs for these or other metal-to-ligand proton shifts were not successful due to very flat PESs, but it seems clear that the very favorable intermolecular deprotonation of **4-H^+^** would occur readily via one or more of these or perhaps other pathways.

## 4 Considerations of Selectivity and Implications for Catalysis

As discussed above, unlike the unsubstituted parent pincer complex (^tBu^PCP)IrHCl,^63^**3-H**, or derivatives, the para-pyridyl-based **2-H** reacts rapidly at ambient temperature with oxidants including O_2_, trityl cation, ferrocenium, and benzoquinone. We attribute the facile oxidation of **2-H** to a preceding MLPT reaction. The equilibrium of the MLPT reaction lies toward **2-H** rather than **2-H-t**. This stands in contrast to 2-pyridinyl^64,65^ and imidazolyl metal hydride complexes^32,33,36^ where proton transfer from metal to ligand is favored at equilibrium. Recently, Kuo and Goldberg have reported an iridium pincer system, based on a bis(pyrazolyl)pyridine ligand, in which both Ir(III)-H and (L-H)Ir(I) tautomers are observable.^29^ But while the formation of **2-H-t** is calculated to be slightly endergonic (Δ*G*° = 6.5 kcal/mol), DFT calculations predict that its oxidation is far more favorable than that of **2-H** (by 31.3 kcal/mol), to give **H^+^2^•^** which is then deprotonated to give **2^•^**. Oxidation of the resulting Ir(II) complex **2^•^** is calculated to yield intermediate, **2^+^_bent_** , which is calculated to readily insert into an sp^3^ C–H bond of a phosphino-*t*-butyl group to produce the highly acidic species **4-H**^+^, which upon deprotonation gives the observed cyclometalated complex **4**. The oxidation of **2-H-t** is not observed by CV since its equilibrium concentration at the electrode is too small.

**Scheme 10:**
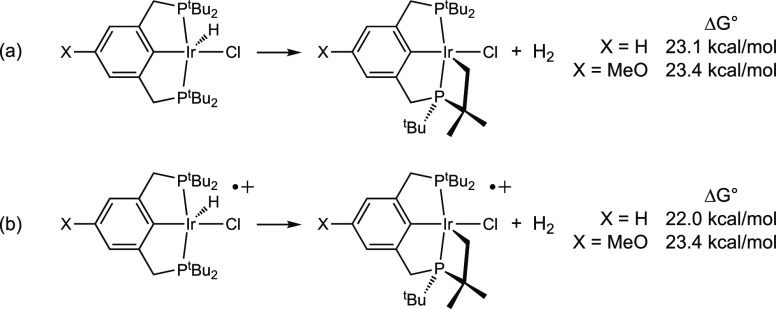
Thermodynamics of Dehydrogenative Cyclometalation of (X-^tBu^PCP)IrHCl and Their Corresponding One-Electron Oxidation Products

It is possible that cationic, d^6^ pincer iridium complexes similar to **2^+^_bent_** have been involved in prior examples of cyclometalation reactions without being identified. For example, Koridze and coworkers reported that treatment of (^tBu^POCOP)IrHCl and (EtO_2_C-^tBu^POCOP)IrHCl with trifluoroacetic acid leads to cyclometalation with loss of H_2_.^49^ Mayer and coworkers undertook cyclic voltammetry studies of (^tBu^PCP)IrHCl and (MeO-^tBu^PCP)IrHCl, in which they found that oxidation leads to cyclometalation with loss of H_2_.^43,66,67^ They proposed that upon one-electron oxidation, cyclometalation to yield H_2_ (Scheme 10, eq b) becomes thermodynamically favorable. Our calculations, however, strongly indicate that the thermodynamics of such dehydrogenative cyclometalation reactions are substantially endergonic and strikingly unaffected by single-electron oxidation (Scheme 10).

It is important to note that [(pN-^tBu^PCP)Ir(*III*)Cl^+^], **2^+^**, undergoes *intra*molecular C–H activation, in contrast with Ir(I) complexes of isostructural ^tBu^PCP or ^tBu^POCOP ligands which are among the most well-known complexes for *inter*molecular C–H activation (and functionalization).^68−70^ The different reactivity is undoubtedly not based on electronic factors; the C–H bond of a *t*-butyl group is certainly not very different than that of an *n*-alkane, with respect to electronic factors, in any way that would strongly bias its relative reactivity toward an Ir(III) versus an Ir(I) center. Presumably the preference of the Ir(III) species to undergo intramolecular C–H addition, in contrast with the Ir(I) fragments, is based on the very different geometries and steric environments of the respective vacant coordination sites that enable the C–H addition reactions. Specifically, the active site in the case of (pincer)Ir(I) complexes is trans to the pincer aryl carbon, as compared to a site cis to the analogous carbon in the case of [(pN-^tBu^PCP)Ir(*III*)Cl^+^]. The trans site of (PCP)Ir(I) complexes is significantly less crowded than the cis sites,^71^ allowing intermolecular access to an alkane. Conversely, the coordination site cis to the pincer ipso carbon is easily accessible to a phosphino-*t*-butyl group, while cyclometalation, in contrast with intermolecular C–H activation (in this case or generally^72,73^), is not disfavored by crowding.

**Figure 7:**
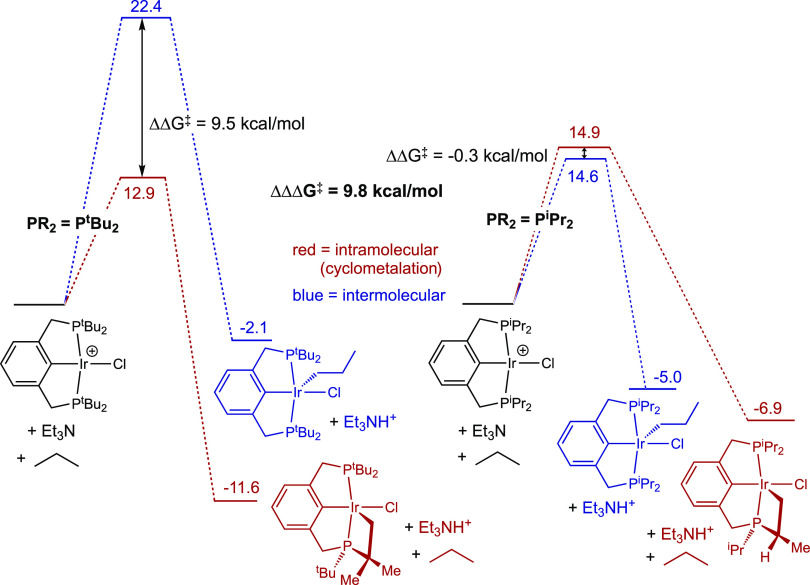
Free energies (kcal/mol) of intramolecular (cyclometalation) versus intermolecular C–H activation by (^R4^PCP)IrCl^+^; R = ^i^Pr and ^t^Bu; M06L-D3 in *n*-heptane continuum (Et_3_N used as a model base; the choice of base has no effect on values of Δ*G*^‡^ or relative values of Δ*G*°).

Calculations were conducted with the parent fragment (^tBu^PCP)IrCl (**3**) to explore and quantify the proposed role of steric factors and the ligand architecture. Cyclometalation by **3^+^** (coupled with deprotonation by triethylamine as a model base) is found to be 9.5 kcal/mol more exergonic than the addition of a propane primary bond,^74,75^ while the kinetic barrier to cyclometalation, Δ*G*^‡^, is also (somewhat coincidentally) 9.5 kcal/mol less (Figure 7). In contrast, for the less crowded di-isopropyl-phosphino analogue, (^iPr^PCP)IrCl^+^, cyclometalation is calculated to have a kinetic barrier very slightly *greater* (ΔΔ*G*^‡^ = 0.3 kcal/mol) than the barrier to the addition of a propane primary bond (which is calculated to be quite low, Δ*G*^‡^ = 14.6 kcal/mol). Thus, in comparing [(^tBu^PCP)IrCl^+^] versus [(^iPr^PCP)IrCl^+^], the calculated difference in selectivity for intermolecular versus intramolecular C–H activation is quite pronounced, 9.8 kcal/mol (expressed as ΔΔΔ*G*^‡^; Figure 7). The thermodynamic preference for cyclometalation by **3^+^** versus intermolecular C–H addition is also much greater than for (^iPr^PCP)IrCl^+^, ΔΔ*G*° = −9.5 kcal/mol versus −1.9 kcal/mol (ΔΔΔ*G*° = 7.6 kcal/mol).

**Scheme 11:**
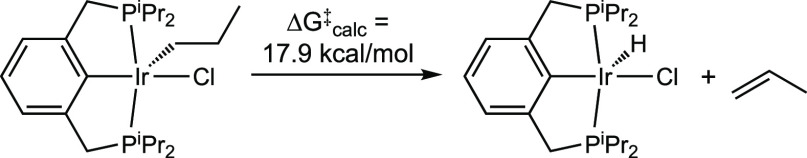
β-H-Elimination by (Hypothetical) Product of C–H Addition/Deprotonation by (^iPr^PCP)IrCl^+^

The reversal of selectivity in C–H activation, from intramolecular to intermolecular by the simple substitution of *i*-Pr groups for *t*-Bu groups, demonstrates that the observation of cyclometalation does not reflect an intrinsic tendency of cationic Ir(III) centers to necessarily undergo cyclometalation. This has important implications for exploiting, for catalytic applications, the unusual reactivity described in this work. For example, successive oxidations and loss of a proton from a more cyclometalation-resistant analogue of **2-H**, if followed by the addition of an *n*-alkyl C–H bond which would yield a species analogous to **4**, of the general form (PCP)IrCl(*n*-alkyl). β-H elimination by this 16-electron species, in analogy with the well-known behavior of (PCP)IrH(*n*-alkyl) complexes,^68−70^ would return the hydride reactant; in the case of (^iPr^PCP)IrCl(*n*-Pr), the calculated barrier, Δ*G*^‡^, is a modest 17.9 kcal/mol (Scheme 11). These reactions would comprise a catalytic cycle for alkane dehydrogenation, driven by two proton-coupled electron transfers.

One could envision this by applying in an electrochemical system or a purely chemical system driven by O_2_ as the ultimate oxidant (directly, or indirectly as in the case of the Wacker reaction system^76^). We have previously reported that (^tBu^PCP)IrH_2_ can catalyze PCET-driven alkane dehydrogenation;^77^ however, that catalyst operates via an Ir(I) species which is subject to overoxidation, and thus far it requires a strong base for deprotonation. The high-oxidation-state cycle envisioned here might circumvent either or both of these issues.

## 5 Conclusions

(pN-^tBu^PCP)IrHCl, **2-H**, is intrinsically more difficult to oxidize (i.e., has a higher oxidation potential or free energy of one-electron oxidation) than the phenyl-based analogue (^tBu^PCP)IrHCl or the even more closely related derivative, **3′-H**. Nevertheless, **2-H** displays much greater reactivity with oxidants including ferrocenium, trityl cation, and benzoquinone, as well as O_2_.

DFT calculations reveal the key intermediacy of an Ir(I) tautomer, resulting from MLPT, which can be characterized as either a zwitterion or a remote NHC complex. This tautomer is subject to facile one-electron oxidation to give the net loss of a hydrogen atom from the iridium center. The resulting product is a cationic N-protonated iridium(II) complex **H^+^2^•^**. Deprotonation then gives **2^•^**, which is also fairly easily oxidized, to give **2^+^**, completing the net loss of a hydride from **2-H**. The 4-coordinate d^6^ iridium complex **2^+^** is calculated to undergo cyclometalation/C–H addition to give the strongly acidic complex **4-H^+^**. Loss of a proton from **4-H^+^** gives the observed cyclometalated iridium(III) complex **4**.

PCP-type pincer ligands have yielded a very rich manifold of chemistry, particularly for the activation of small molecules and especially C–H bonds.^68−70^ The para-pyridyl pincer ligand appears to add a new dimension to this manifold by allowing the low-energy deprotonation of the metal center of a hydride complex, via MLPT. In this work, we see that such deprotonation facilitates oxidation, leading to the net loss of H• and ultimately H^**–**^, to give a highly reactive coordinatively unsaturated cationic metal center.

## Supporting Information Available

Complete experimental details and synthetic procedures, NMR data, computational details, computed energies and thermodynamic quantities (PDF)
Optimized structures for calculated species (.mol format) (ZIP)
