# Supplementary material for: Metal–Ligand Proton Tautomerism, Electron Transfer, and C(sp3)–H Activation by a 4-Pyridinyl-Pincer Iridium Hydride Complex
Source: J Am Chem Soc. 2023 Aug 8;145(33):18296–306. doi: 10.1021/jacs.3c03376 (PMC10450815; doi:10.1021/jacs.3c03376)
Supplement: Supplementary file 1 — ja3c03376_si_001.pdf [file ja3c03376_si_001.pdf]

Supporting Information

Metal-Ligand Proton Tautomerism, Electron-Transfer, and C(sp^3)-H Activation by a 4 Pyridinyl-Pincer Iridium Hydride Complex

Tariq M. Bhatti[a], Akshai Kumar[b], Ashish Parihar[a], Hellan K. Moncy[a], Thomas Emge[a], Kate M. Waldie[a], Faraj Hasanayn[c*], Alan S. Goldman[a,b*]

[a] *Department of Chemistry and Chemical Biology, Rutgers, The State University of New Jersey, Piscataway, New Jersey 08854, United States*

[b] *Centre for Nanotechnology, Indian Institute of Technology Guwahati, Guwahati, 781039, Assam, India*

[c] *Department of Chemistry, American University of Beirut, Beirut 1107 2020, Lebanon*

*Email: fh19@aub.edu.lb; alan.goldman@rutgers.edu

General Information 3
Synthesis of compounds 4
2,6-dimethyl-3,5-bis(methylol)pyridine (*Scheme 1*) 4
2,6-dimethyl-3,5-bis(bromomethyl)pyridine (*Scheme 1*) 6
Proligand 1 (*Scheme 1*) 8
Complex 2-H (*Scheme 1*) 10
1,3-bis(di-tert-butylphosphinomethyl)-4,6-dimethylbenzene (“dm-tBuPCP”) 13
Complex 3’-H 15
Compounds prepared through literature methods 17
Materials from chemical suppliers: 18
Reactions of 2-H in Table 1: 19
Entry 1: $[Cp_2Fe^+][PF_6^-]$ (1 eq.) (*Scheme 4)* 19
Entry 2: $[Cp_2Fe^+][PF_6^-]$ (2 eq.) 21
Entry 3: $[Cp_2Fe^+][PF_6^-]$ (2 eq.) + 2,6-lutidine (9 eq.) (*Scheme 5*) 25
Entry 4: Trityl $BArF^{20}$ (1 eq.) (*Scheme 6*) 26
Entries 5 and 6: Benzoquinone (1 eq.), 40 mins and 48 hours 29
Entry 7: $[(p\text{-MeO-}C_6H_4)_3C^+][BF_4^-]$ (3 eq.) 31
Entry 8: $[Cp^*{}_2Fe^+][BF_4^-]$ (2 eq.) 32
Observations of iridium(II) intermediate 2• 33
Reaction of 2-H (7 mM) with benzoquinone (540 mM) 33
Quenching iridium(II) intermediate with TEMPO-H to return 2-H 35
Scheme 9. Reaction of 2-H with 2,4,6-tri-*tert*-butylphenoxy radical. 38

Cyclic voltammetry 41
Computational Methods 44
X-Ray Diffraction Data 46
Proligand 1 47
Table S2. Crystal data and structure refinement for Proligand 1 48
Table S3. Atomic coordinates (x 104) and equivalent isotropic displacement parameters ($Å^2$x 10^3) 49
Table S4. Bond lengths [Å] and angles [°] 50
Table S5. Anisotropic displacement parameters ($Å^2$x 10^3). 53
Table S6. Hydrogen coordinates (x 10^4) and isotropic displacement parameters ($Å^2$x 10^3) 54
Table S7. Torsion angles [°] 55
Complex 2-H 56
Table S8. Crystal data and structure refinement 57
Table S9. Atomic coordinates (x 10^4) and equivalent isotropic displacement parameters ($Å^2$x 10^3) 58
Table S10. Bond lengths [Å] and angles [°] 59
Table S11. Anisotropic displacement parameters ($Å^2$x 10^3). 63
Table S12. Hydrogen coordinates (x 10^4) and isotropic displacement parameters ($Å^2$ x 10^3) 64
Table S13. Torsion angles [°] 66
Table S14. Hydrogen bonds [Å and °]. 68
Complex 4 69
Table S15. Crystal data and structure refinement 70
Table S16. Atomic coordinates (x 10^4) and equivalent isotropic displacement parameters ($Å^2$x 10^3) 71
Table S17. Bond lengths [Å] and angles [°] 73
Table S18. Anisotropic displacement parameters ($Å^2$x 10^3) 78
Table S19. Hydrogen coordinates (x 10^4) and isotropic displacement parameters ($Å^2$x 10^3) 80
Table S20. Torsion angles [°] 82
Table S21. Hydrogen bonds 84

General Information

Unless otherwise specified, all procedures involving organometallic compounds were conducted under an argon atmosphere using an MBraun glovebox or Schlenk line. Solvents were purchased as anhydrous grade and purged with argon before use. Benzene-d_6 was degassed via freeze-pump-thaw cycles and then dried over 3Å molecular sieves prior to use. Ultra-high purity hydrogen was purchased from Airgas and used as received. ^{1}H, ^{13}C, ^{31}P, COSY, and NOESY/EXSY NMR spectra were recorded on a 500 MHz Varian spectrometer. Chemical shifts are reported in ppm. ^{13}C NMR and ^{1}H NMR signals are referenced to the residual solvent signals, ^{31}P NMR signals are referenced to an external standard of 85% H_3PO_4. Microanalysis samples were weighed with a PerkinElmer Model AD6000 Autobalance and their compositions were determined with a PerkinElmer 2400 Series II Analyzer.

Synthesis of compounds

2,6-dimethyl-3,5-bis(methylol)pyridine (*Scheme 1*)

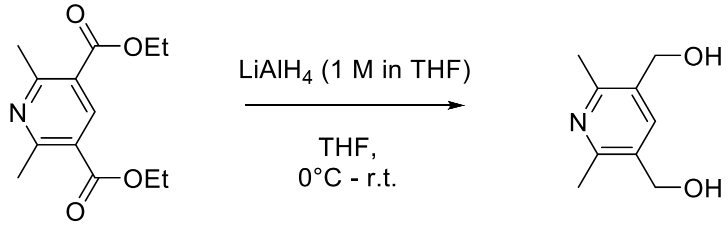

Lithium aluminum hydride (1 M in THF, 25 mL, 25 mmol) was added to a 100-mL Schlenk flask and then cooled to 0 °C in an ice bath. Diethyl 2,6-dimethylpyridine-3,5-dicarboxylate (5.1 g, 20.3 mmol) was dissolved into 25 mL of anhydrous THF and loaded into an addition funnel. The ester solution was added, dropwise, to the LAH solution with stirring. The addition proceeds smoothly with little effervescence. Upon completion of the addition, the mixture was allowed to stir overnight. The following day, it was quenched by sequential addition of approximately 0.9 mL water (dropwise at first), then 0.9 mL 15% NaOH, and finally 2.7 mL of additional water. After stirring for 15 minutes, the overlying solvent was removed by rotary evaporation. The resulting solids were dissolved in 100 mL boiling methanol and vacuum filtered through celite. An additional 75 mL methanol was used to complete the transfer. 75 mL of acetonitrile was added to the filtrate, and the combined solution was concentrated on a rotavap until it became syrupy (total mass = 15 g). As it cools, crystals spontaneously start to form. This syrup was then dried under vacuum on the Schlenk line, leading to copious white solids. Total mass of product = 3.5 g.

^{1}H NMR (500 MHz, DMSO-*d*$_6$) δ 7.61 (s, 1H), 4.45 (s, 4H), 2.33 (s, 6H).

NOTES:

1. This product is a respiratory irritant. Care should be taken when weighing out dried material.
2. This product is soluble in methanol, dimethylsulfoxide, dimethylformamide, and N-methylpyrrolidone. It is insoluble in diethyl ether, tetrahydrofuran, dichloromethane, chloroform, benzene, acetone, or acetonitrile.
3. In multiple preparations of this compound, it invariably contains residual alumina even after iterative methanol extractions and methanol-acetonitrile precipitations. It may be used in the next step without further purification.
4. This compound has been prepared previously using different workups. See:
   - Illa, G. T.; Hazra, S.; Satha, P.; Purohit, C. S., *CrystEngComm* **2017,** *19* (32), 4759-4765. DOI: 10.1039/C7CE01075C
   - Wang, J.-H.; Li, M.; Zheng, J.; Huang, X.-C.; Li, D., *Chemical Communications* **2014,** *50* (65), 9115-9118. DOI: 10.1039/C4CC04100C
5. This compound has also been prepared via catalytic hydrogenation:
   Elangovan, S.; Wendt, B.; Topf, C.; Bachmann, S.; Scalone, M.; Spannenberg, A.; Jiao, H.; Baumann, W.; Junge, K.; Beller, M., *Advanced Synthesis & Catalysis* **2016,** *358* (5), 820-825. DOI: 10.1002/adsc.201500930

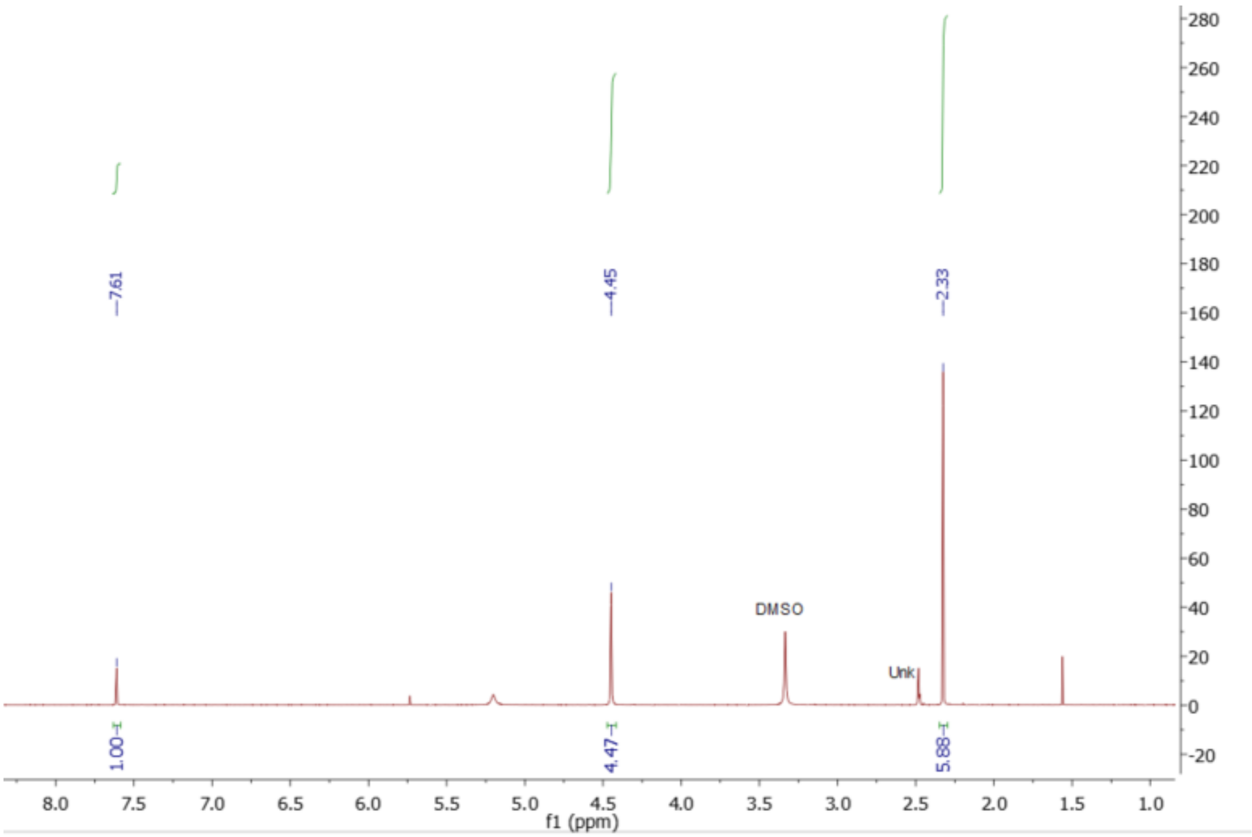

Figure S1: ^{1}H-NMR of 2,6-dimethyl-3,5-bis(methylol)pyridine in DMSO-d_6.

2,6-dimethyl-3,5-bis(bromomethyl)pyridine (*Scheme 1*)

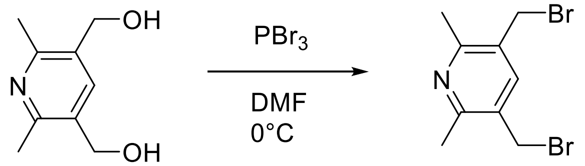

2,6-dimethyl-3,5-bis(methylol)pyridine (638 mg, 3.82 mmol) was poured into a flame-dried 100-mL Schlenk flask. The solid was suspended into 20 mL anhydrous DMF, sonicated to homogenize, and cooled to 0°C in an ice-water bath. The atmosphere was purged with argon. Phosphorus tribromide (0.72 mL, 7.7 mmol) was added by syringe, slowly, with stirring (strong exotherm). The mixture becomes yellow and homogeneous. Stirring was continued at 0°C for 5 minutes, then the flask was warmed to room temperature and allowed to stir for 1 hour. The mixture becomes turbid. The contents were poured into 100 mL of saturated sodium carbonate solution and extracted with 4 x 50 mL ethyl acetate. The pooled ethyl acetate extracts were washed with 4 x 50 mL deionized water, then once with 50 mL of saturated sodium chloride solution, dried over sodium sulfate, and concentrated to ca. ¼ of its initial volume on a rotavap. At this point, 75 mL of n-heptane was added. The solution was then evaporated until copious white crystals precipitate from solution. The overlying solvent is decanted, the crystals are washed with cold heptane, and dried under vacuum. Yield: 863 mg, 78% yield (Note 1).

^{1}H NMR (500 MHz, Chloroform-d) δ 7.50 (s, 1H), 4.45 (s, 4H), 2.59 (s, 6H).

NOTES:

1. Yields of 5 additional batches: 66.7%, 65.0%, 65.8%, 67% (5 min reaction time), 82.3%. Average = 71%.
2. Reaction fails in wet solvent.
3. This compound has been prepared before using dioxane as solvent.See:
    - Illa, G. T.; Hazra, S.; Satha, P.; Purohit, C. S., *CrystEngComm* **2017,** *19* (32), 4759-4765. DOI: 10.1039/C7CE01075C
4. N-Methylpyrrolidone is a good substitute for DMF.
5. Purity of this intermediate is essential for purity of subsequent products. Although the above workup usually affords white, crystalline material, less pure product may be further purified through flash chromatography on silica gel using 1:1 v/v Ethyl acetate/heptane (Rf = 0.4).

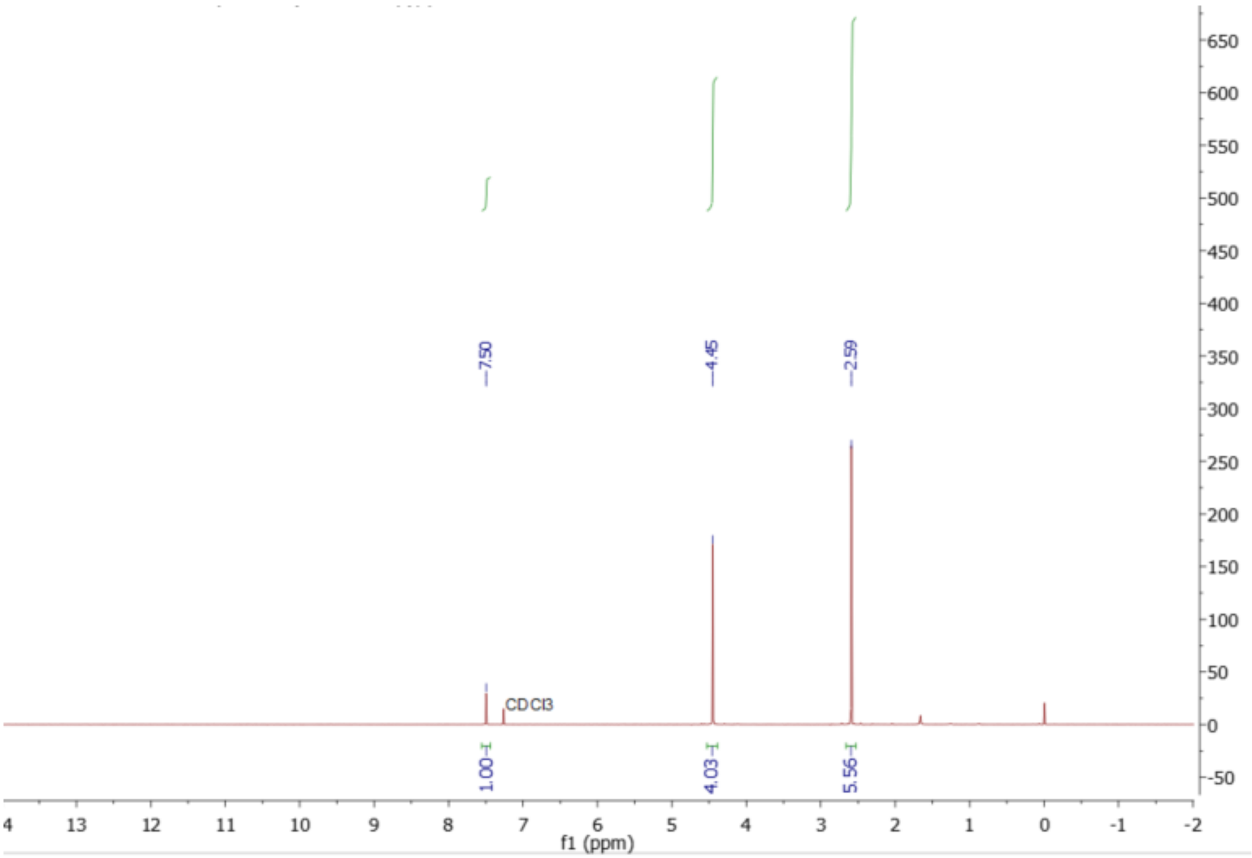

Figure S2: ^{1}H-NMR of 2,6-dimethyl-3,5-bis(bromomethyl)pyridine in chloroform-d.

Proligand 1 (*Scheme 1*)

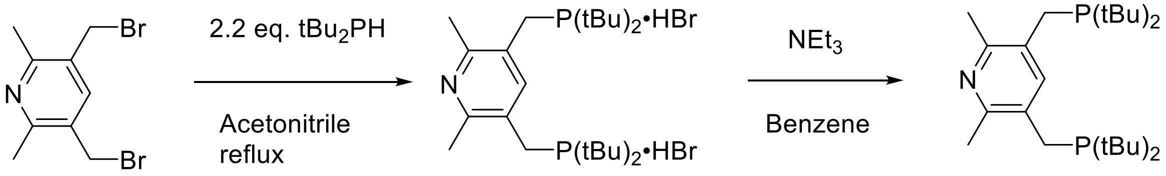

2,6-dimethyl-3,5-bis(bromomethyl)pyridine (2.49 g, 8.6 mmol) was dissolved into 200-250 mL acetonitrile. Ditertbutylphosphine (4.0 mL, 2.5 eq.) was added via syringe. The mixture was heated to reflux.

After 17 hours, the mixture was a suspension of copious, fluffy, white precipitates. Stirring was stopped and the solids were allowed to settle. Then, the overlying liquid was decanted off by cannula filtration.[1] The residual solids were dried under vacuum. The white solids were suspended in 120 mL benzene and 3.6 mL triethylamine (3 eq. with respect to 2,6-dimethyl-3,5-bis(bromomethyl)pyridine) was added by syringe. The mixture was refluxed overnight.[3] Then, the mixture was cannula filtered and evaporated to dryness. Isolated yield: 1.7 g, 4.0 mmol, 47%. Calculated (wt. %): **C**: 70.89, **H**: 11.18, **N**: 3.31. Found (wt. %): **C**: 70.43, **H**: 10.93, **N**: 3.33.

Residues were washed from sides of flask with benzene and allowed to evaporate to dryness under an argon atmosphere, affording X-Ray quality crystals.

^{31}P NMR (202 MHz, Benzene-d_6) δ 26.79 .

^{1}H NMR (500 MHz, Benzene-d_6) δ 8.04 (t, J = 2.5 Hz, 1H), 2.74 (s, 6H), 2.64 (d, J = 2.3 Hz, 4H), 1.02 (d, J = 10.6 Hz, 36H).

^{13}C NMR (126 MHz, Benzene-d_6) δ 152.68 (t, J = 2.4 Hz), 139.89 , 131.21 (d, J = 11.5 Hz), 31.52 (d, J = 24.4 Hz), 29.55 (d, J = 13.5 Hz), 24.85 (d, J = 26.6 Hz), 22.83 (d, J = 5.2 Hz).

NOTES:

1. On standing, crystals of the HBr salt (1 gram) form in the decanted liquid. Including this, overall yield is 74%.
2. Using wet solvent leads to long induction times and may affect yield.
3. 4 hours is sufficient.

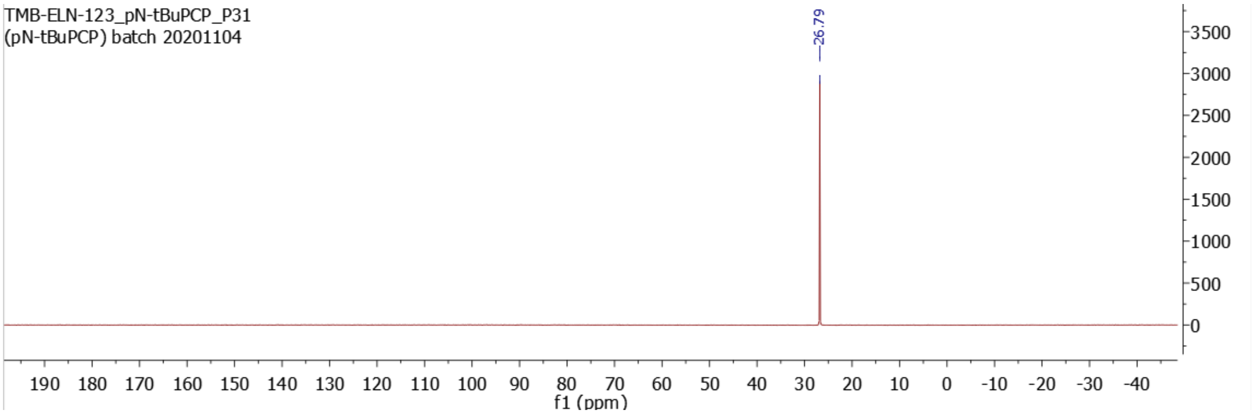

Figure S3: ^{31}P-NMR of pro-ligand ***1*** *in benzene-d6.*

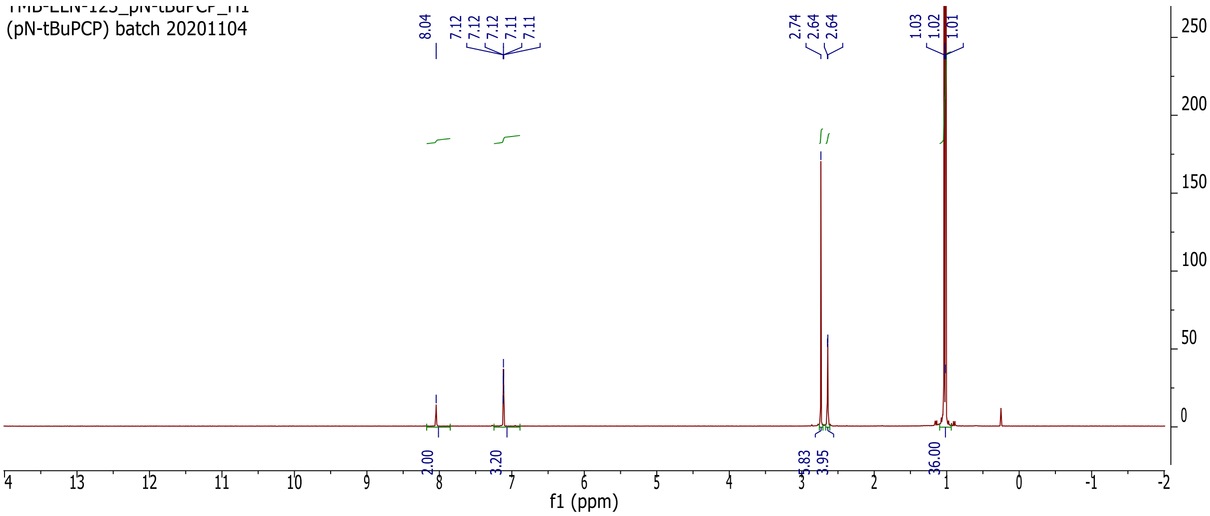

Figure S4: ^{1}H-NMR of pro-ligand ***1*** *in benzene-d6.*

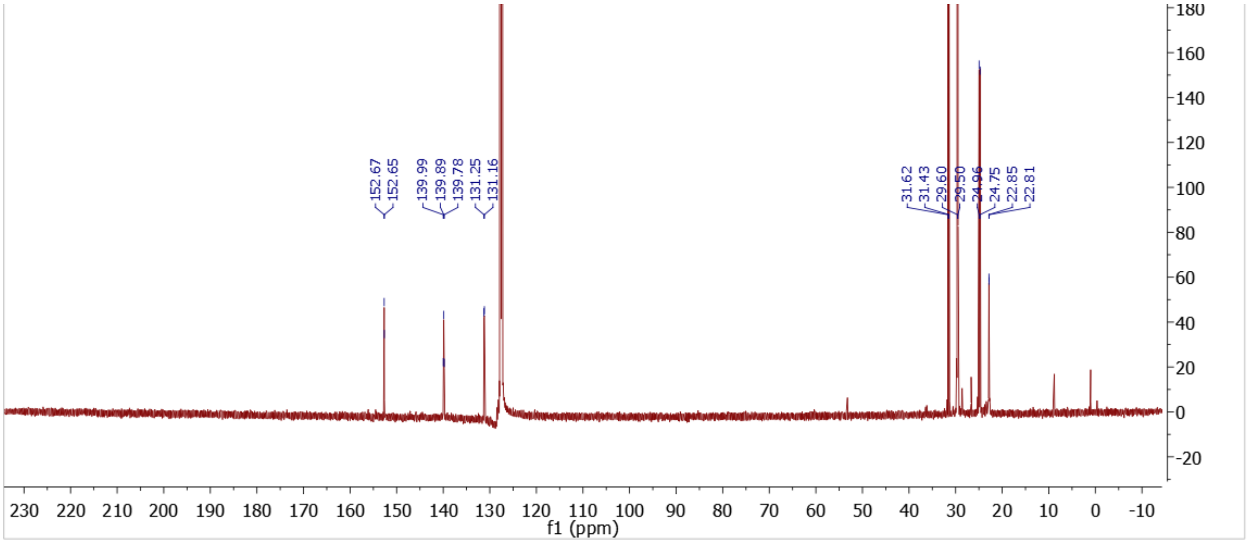

Figure S5: ^{13}C-NMR of pro-ligand ***1*** *in benzene-d6.*

Complex 2-H (*Scheme 1*)

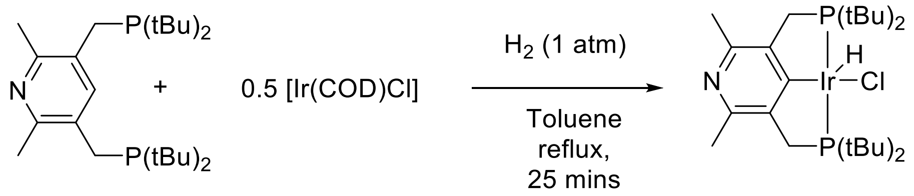

Inside an argon-filled glovebox, ligand (96 mg, 0.46 mmol) and $[Ir(COD)Cl]_2$ (76 mg, 0.24 mmol) were weighed into a 50 mL Schlenk flask and dissolved into 10 mL anhydrous toluene. On a Schlenk line, the solution was purged with hydrogen until the mixture becomes bright red. Then, it was heated to reflux for 25 minutes. After this time, the solution was brought to room temperature and the solution was carefully evaporated to dryness. Inside a glovebox, the orange residues were redissolved into 5-10 mL anhydrous benzene and transferred into a 20 mL screw-cap vial, which was covered with a 24/40 septum. On a Schlenk line, this solution was frozen with liquid nitrogen and lyophilized by applying vacuum through a needle through the septum. Isolated yield: 135 mg, 90%. Calculated (wt. %): **C**: 46.11, **H**: 7.27, **N**: 2.15. Found (wt. %): **C**: 46.33, **H**: 7.17, **N**: 2.09.

X-Ray quality crystals of (pN-tBuPCP)IrHCl were grown from a toluene-pentane solution at -40°C.

^{31}**P NMR** (202 MHz, Benzene-d6) δ 67.84 (d, J = 12.1 Hz).
^{1}**H NMR** (500 MHz, Benzene-d6) δ 2.99 (dt, J = 17.4, 4.0 Hz, -C*H*$_2$-), 2.85 (dt, J = 17.5, 4.0, Hz,-C*H*$_2$-), 2.56 (s, C_{Pyr}-C*H*$_3$), 1.22 (t, J = 6.6 Hz, P-C(C*H*$_3$)$_3$), 1.17 (t, J = 6.8 Hz, P-C(C*H*$_3$)$_3$), -41.86 (t, J = 12.2 Hz, Ir-*H*).
^{13}**C NMR** (126 MHz, Benzene-d6) δ 158.08 , 147.95 , 143.09 , 37.33 (t, J = 10.1 Hz), 34.58 (t, J = 11.1 Hz), 32.04 (t, J = 13.8 Hz), 29.49 , 28.85 , 24.39 .

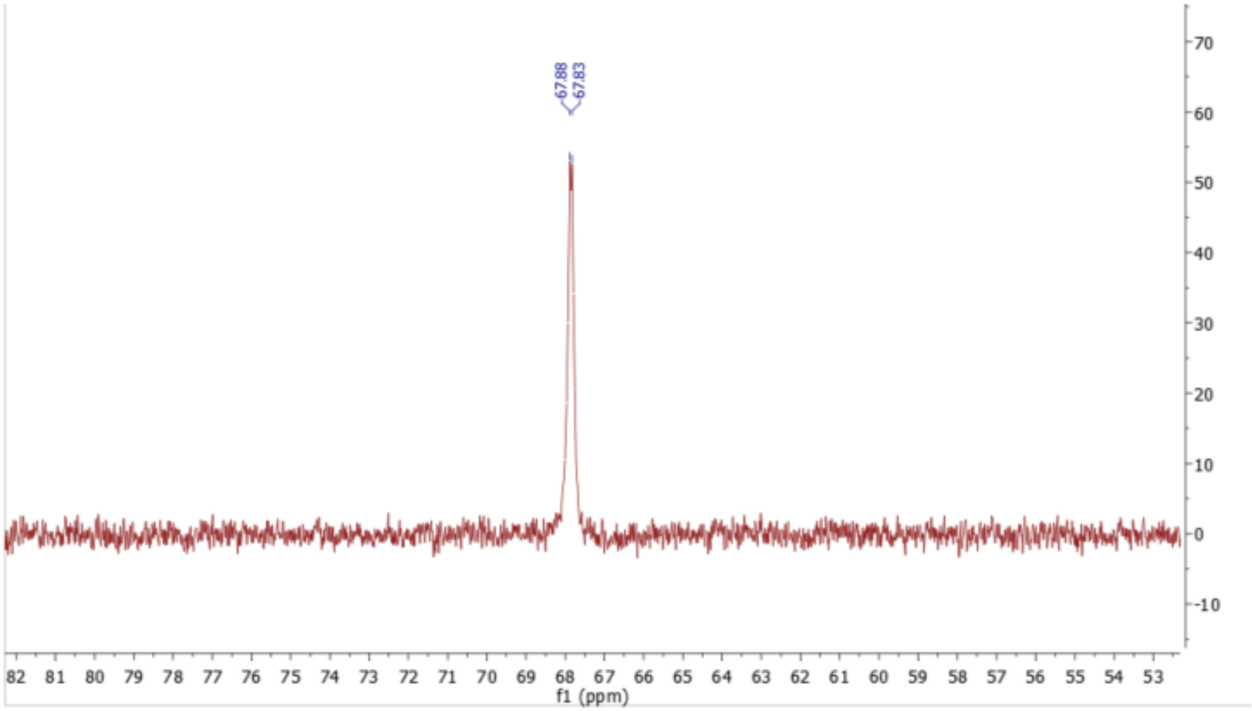

*Figure S6: ^{31}P-NMR of complex **2-H** in benzene-d6.*

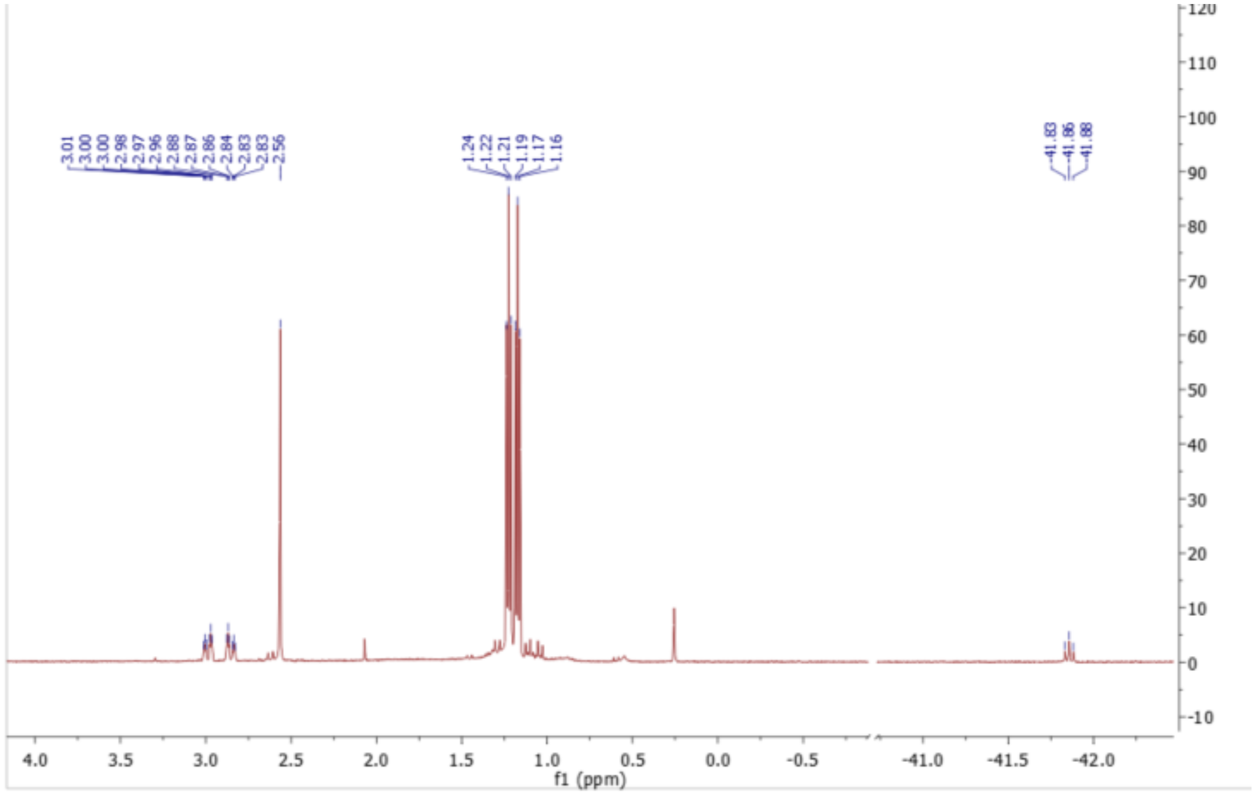

*Figure S7: ^{1}H-NMR of complex **2-H** in benzene-d6. Spectrum contains signals from residual toluene from synthesis..*

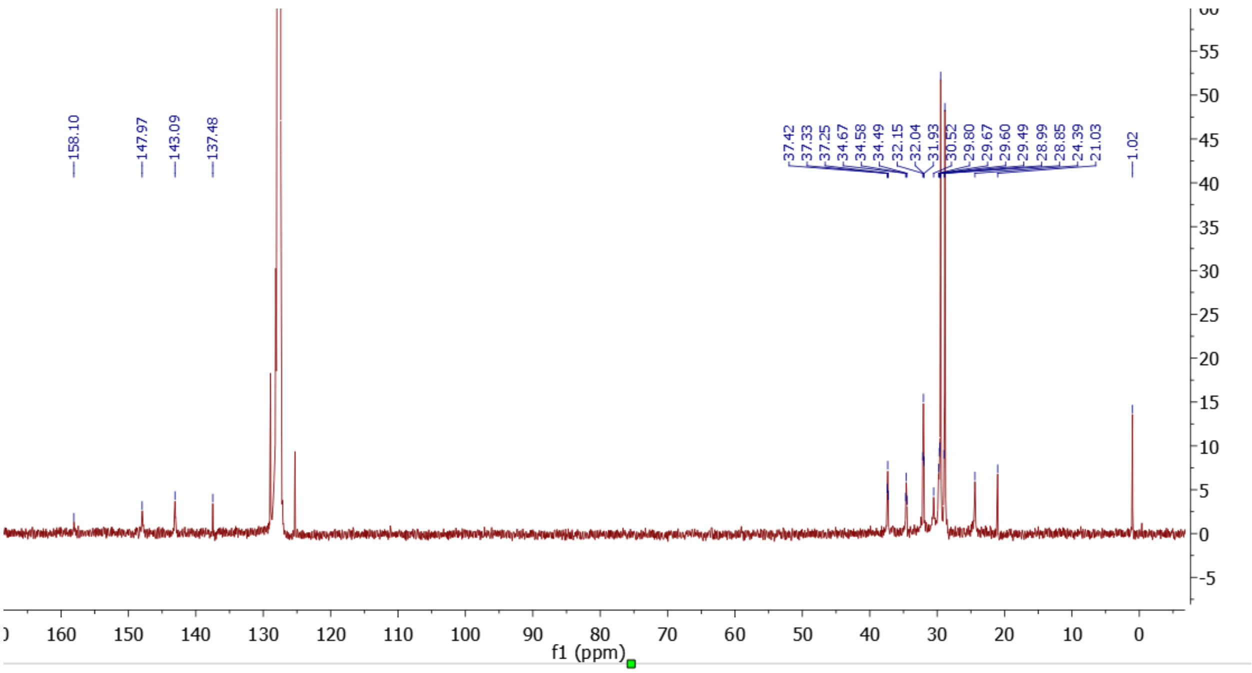

Figure S8: ^{13}C-NMR of complex ***2-H*** *in benzene-d6. Spectrum contains signals from residual toluene from synthesis.*

1,3-bis(di-tert-butylphosphinomethyl)-4,6-dimethylbenzene ("dm-tBuPCP")

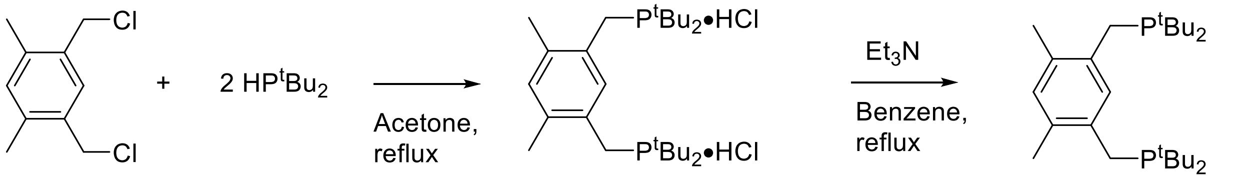

In a 50 mL Schlenk flask, 1,3-bis(chloromethyl)-4,6-dimethylbenzene (300 mg, 1.46 mmol) was dissolved in 20 mL anhydrous acetone and di-tertbutylphosphine (475 µL, 3.21 mmol, 2.2 eq.) was added by syringe. The mixture was heated to reflux for 24 hours, by which time a heavy white precipitate forms. The overlying solvent was evaporated under vacuum and the solids were resuspended in anhydrous benzene. Then, triethylamine (3.5 mL) was added and the mixture was heated to reflux for 6 hours. The resulting suspension was cannula filtered. The filter solids were washed with anhydrous acetone. The combined filtrates are evaporated to collect the product as a white solid. 185 mg, 30% yield. Calculated (wt. %): **C**: 73.89, **H**: 11.45, **N**: 0. Found (wt. %): **C**: 73.20, **H**: 11.54, **N**: 0.04.

^{31}P NMR (162 MHz, Benzene-d$_6$) δ 25.55
^{1}H NMR (400 MHz, Benzene-d$_6$) δ 7.80 (s, 1H), 6.86 (s, 1H), 2.78 (d, J = 2.4 Hz, 4H), 2.39 (s, 6H), 1.08 (d, J = 10.4 Hz, 36H).
^{13}C NMR (126 MHz, Benzene-*d$_6$*) δ 136.56 (d, *J* = 9.8 Hz), 133.65 (t, *J* = 11.6 Hz), 133.42 (t, *J* = 2.0 Hz), 133.14 , 32.00 (d, *J* = 25.0 Hz), 30.09 (d, *J* = 13.6 Hz), 26.45 (d, *J* = 26.6 Hz), 20.34 (d, *J* = 7.1 Hz).

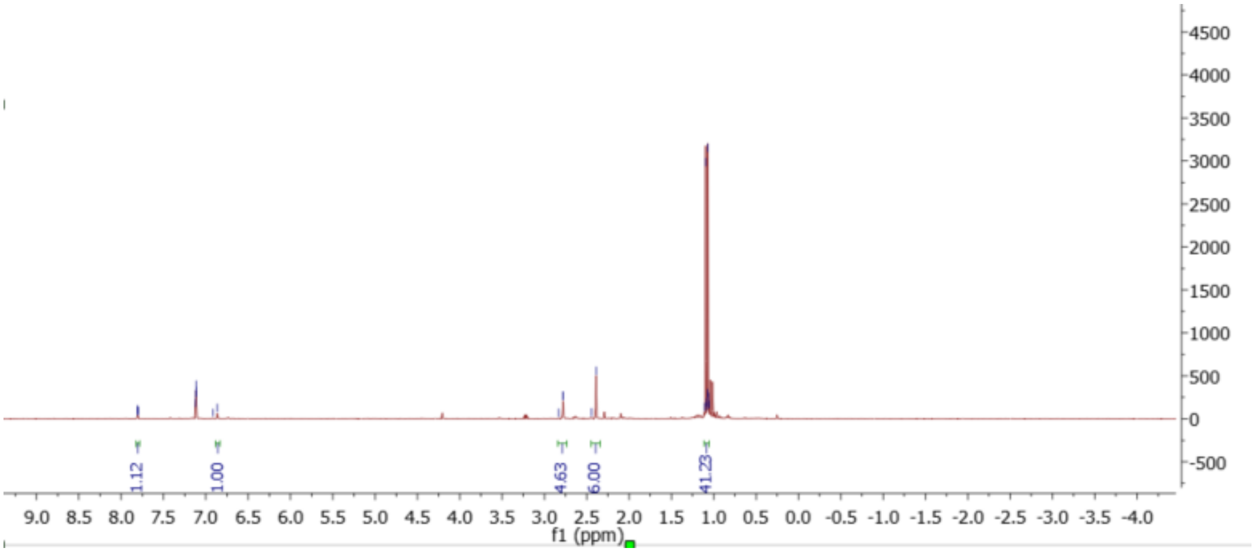

Figure S9: ^{1}H- NMR of ligand dm-tBuPCP in benzene-d6.

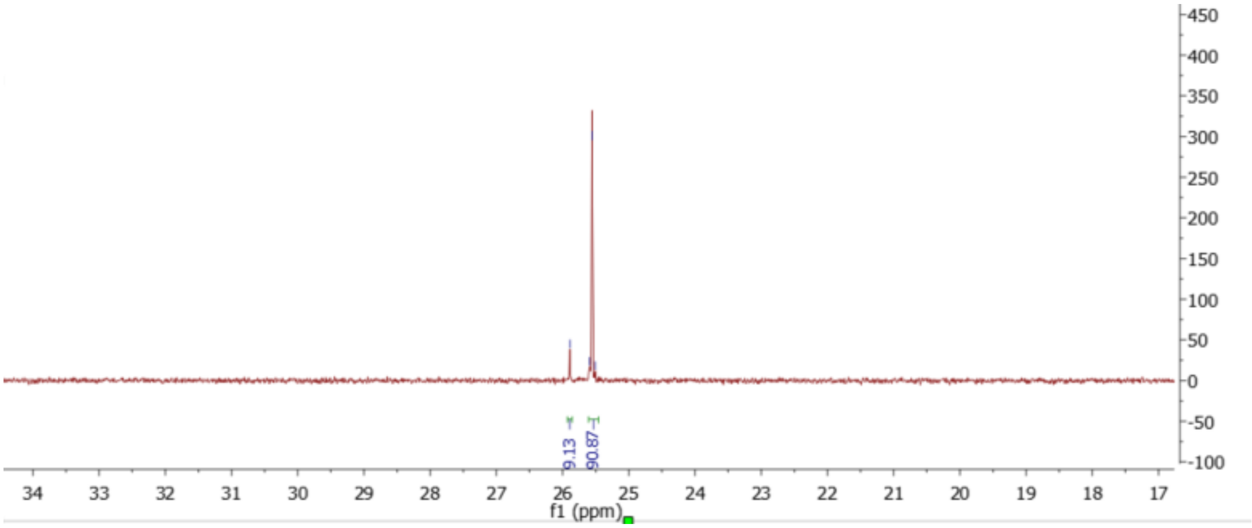

Figure S10: ^{31}P - NMR of ligand dm-tBuPCP in benzene-d6.

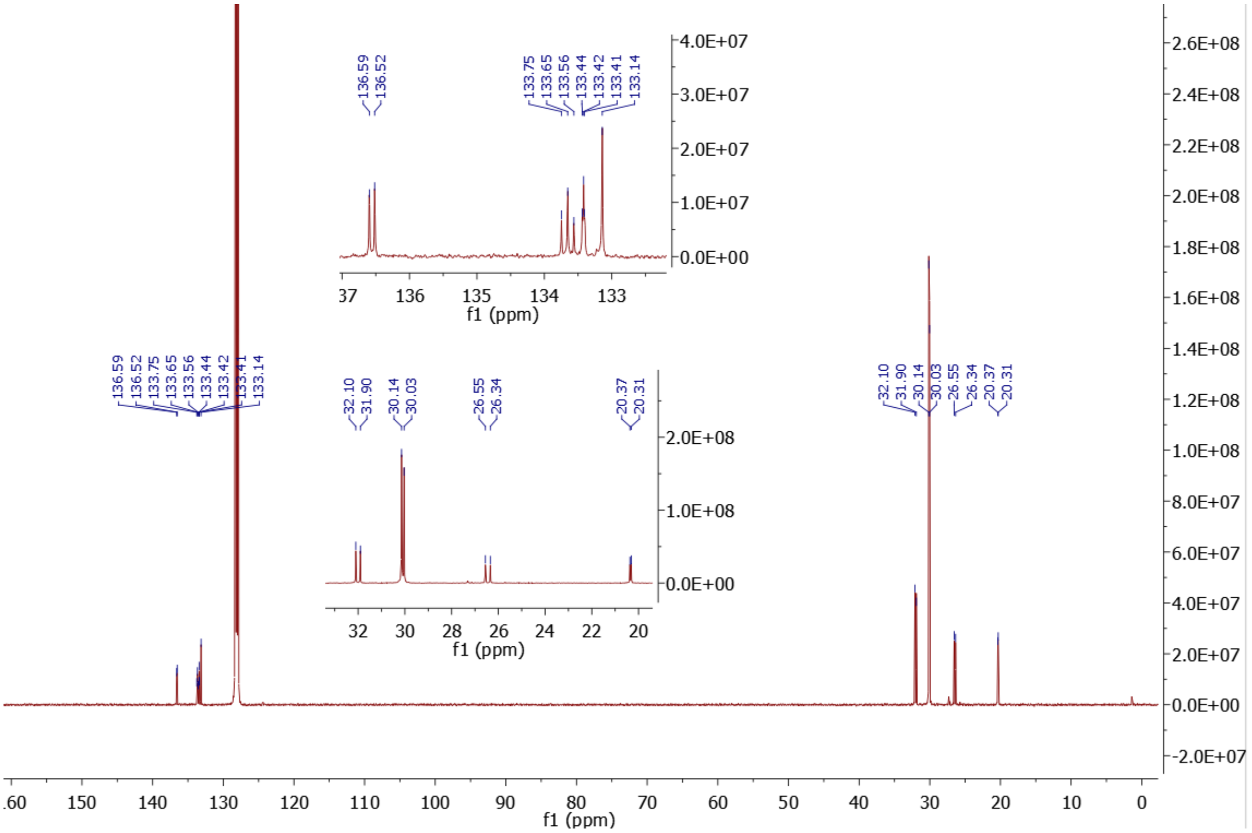

Figure S11: ^{13}C- NMR of ligand dm-tBuPCP in benzene-d6.

Complex 3’-H

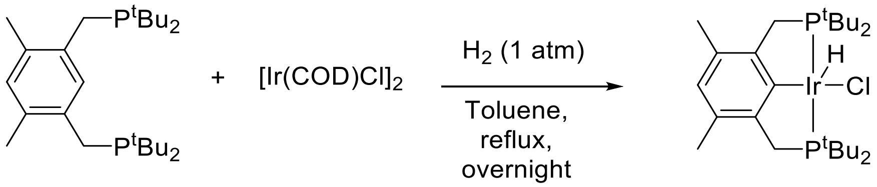

dm-^{tBu4}PCP ligand (254 mg, 0.6 mmol) and $[Ir(COD)Cl]_2$ (200 mg, 0.3 mmol) were weighed into a 50 mL Schlenk flask and dissolved into 25 mL anhydrous toluene inside a glovebox. The flask was heated to 110°C on an oil bath overnight under argon atmosphere. The following morning, the toluene solvent was evaporated under vacuum. The residues were dissolved into 3 x 10 mL pentanes and filtered via cannula. The pentane was evaporated under vacuum, affording an orange-red powder. Yield: 254 mg (88%). Calculated (wt. %): **C**: 48.02, **H**: 7.44, **N**: 0. Found (wt. %): **C**: 48.55, **H**: 7.66, **N**: -0.03.

Product was further purified by recrystallization from hexanes at -40°C.

^{31}P NMR (162 MHz, Benzene-d_6) δ 66.38 (d, J = 12.0 Hz).
^{1}H NMR (400 MHz, Benzene-d_6) δ 3.08 (dt, J = 17.4, 3.8 Hz, 2H), 2.94 (dt, J = 17.5, 4.0 Hz, 2H), 2.21 (s, 6H), 1.29 (t, J = 6.5 Hz, 18H), 1.24 (t, J = 6.7 Hz, 18H), -42.50 (t, J = 12.6 Hz, 1H).
^{13}C NMR (126 MHz, Benzene-d_6) δ 147.55 (t, *J* = 8.5 Hz), 145.72 (d, *J* = 3.7 Hz), 129.47 (t, *J* = 7.3 Hz), 126.35 , 37.30 (t, *J* = 10.2 Hz), 34.50 (t, *J* = 11.2 Hz), 32.90 (t, *J* = 14.1 Hz), 29.71 (t, *J* = 2.9 Hz), 28.97 (t, *J* = 2.1 Hz), 22.09 .

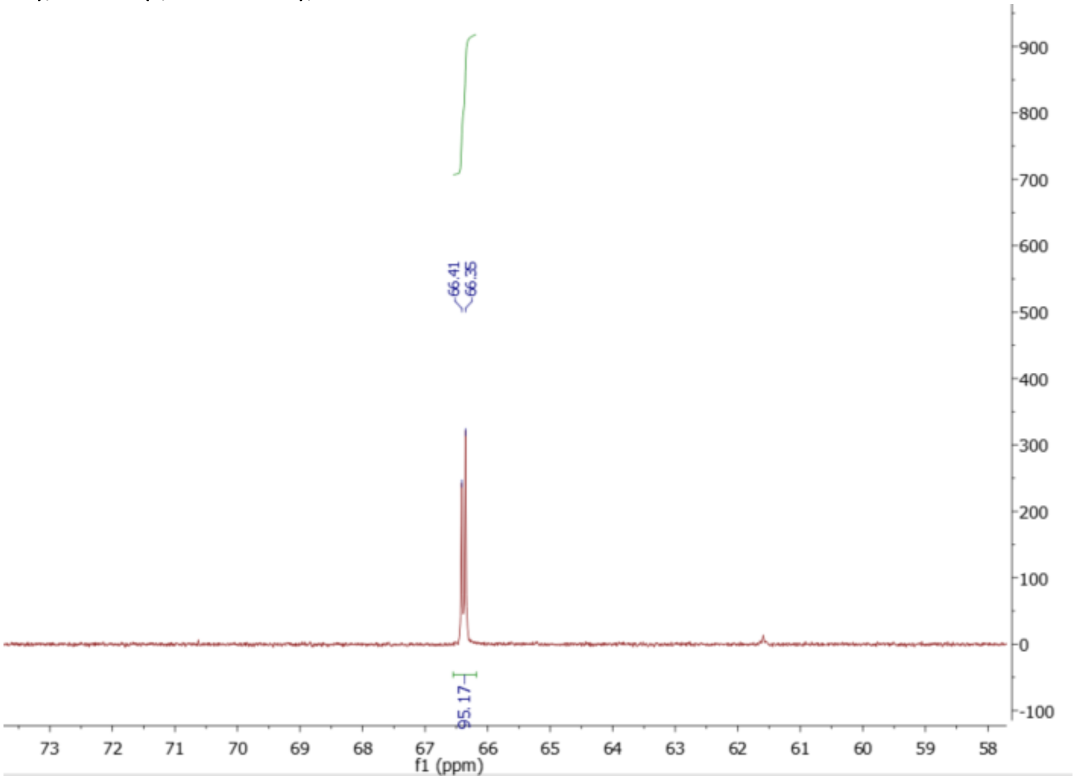

Figure S12: ^{31}P - NMR of complex 3’-H in benzene-d_6.

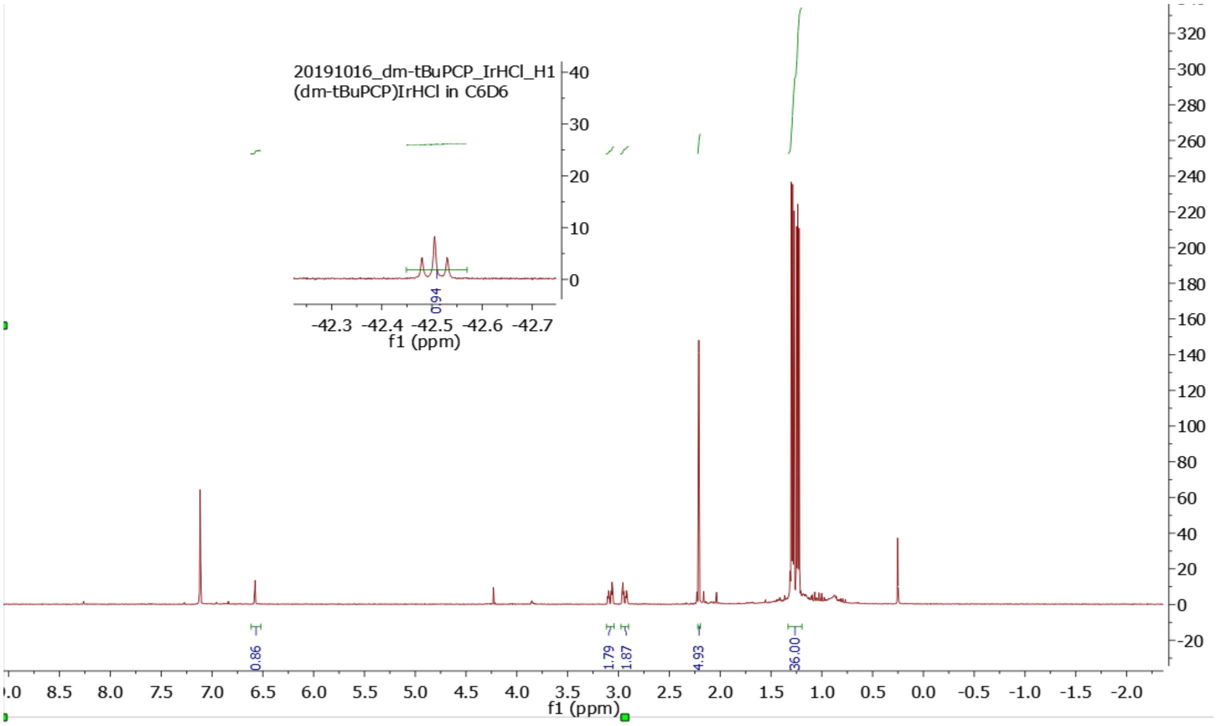

Figure S13: ^{1}H - NMR of complex 3’-H in benzene-d6.

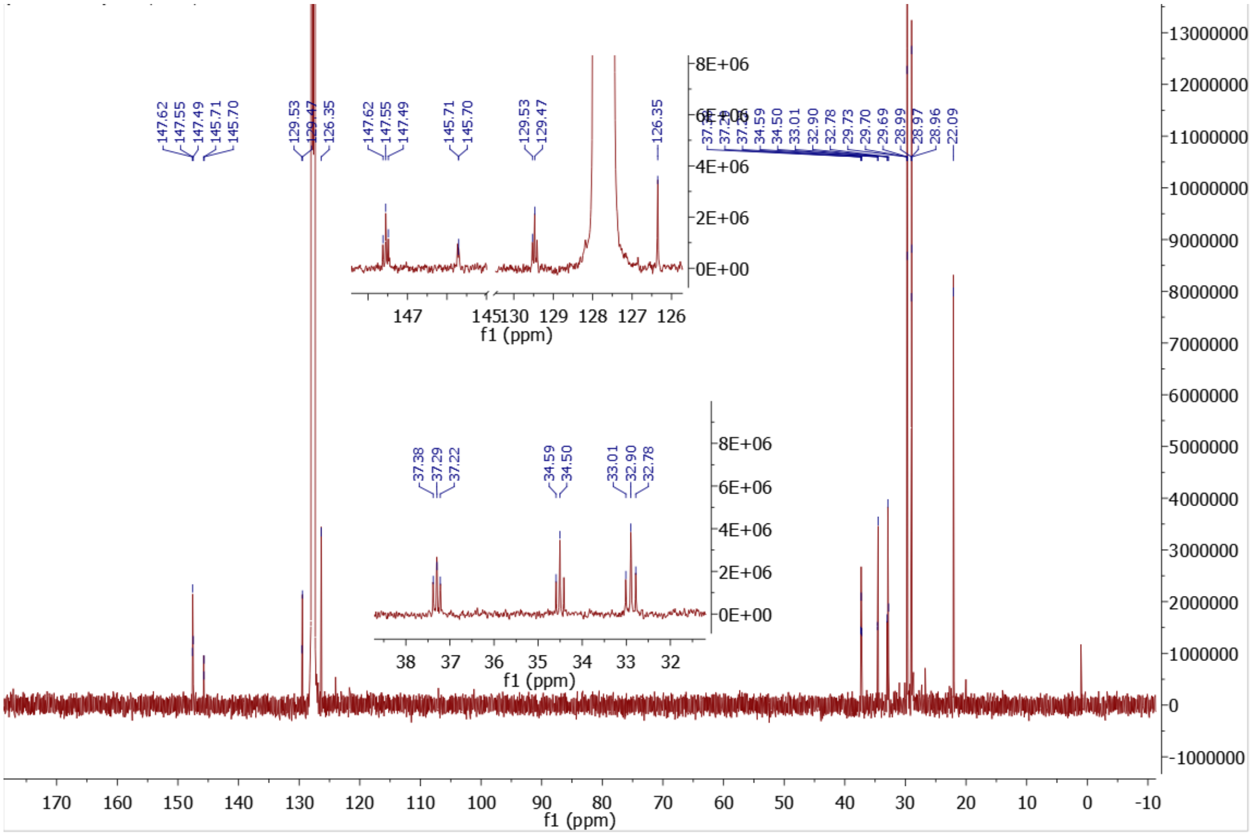

Figure S14: ^{13}C - NMR of complex 3’-H in benzene-d6.

Compounds prepared through literature methods

Compound	Reference	DOI
1,3-bis(chloromethyl)-4,6-dimethylbenzene	Shimizu, T.; Hida, K.; Yamato, T., *Journal of Chemical Research* **2009,** *2009* (7), 443-447.	10.3184/030823409X465222
2,4,6-tri-tertbutylphenoxy radical	Manner, V. W.; Markle, T. F.; Freudenthal, J. H.; Roth, J. P.; Mayer, J. M., *Chemical Communications* **2008,** (2), 256-258.	10.1039/B712872J
Decamethylferrocene	Goodwin, C. A. P.; Giansiracusa, M. J.; Greer, S. M.; Nicholas, H. M.; Evans, P.; Vonci, M.; Hill, S.; Chilton, N. F.; Mills, D. P., *Nature Chemistry* **2021,** *13* (3), 243-248.	10.1038/s41557-020-00595-w
Decamethylferrocenium tetrafluoroborate	Weiss, C. J.; Das, P.; Miller, D. L.; Helm, M. L.; Appel, A. M., *ACS Catalysis* **2014,** *4* (9), 2951-2958.	10.1021/cs500853f
TEMPO-H	Giffin, N. A.; Makramalla, M.; Hendsbee, A. D.; Robertson, K. N.; Sherren, C.; Pye, C. C.; Masuda, J. D.; Clyburne, J. A. C., *Organic & Biomolecular Chemistry* **2011,** *9* (10), 3672-3680.	10.1039/C0OB00999G
Methoxytrityl alcohol	Akhrem, I. S.; Avetisyan, D. e. V.; Afanas'eva, L. V.; Artyushin, O. I., *ChemistrySelect* **2020,** *5* (26), 7835-7839.	10.1002/slct.201904255
Methoxytrityl tetrafluoroborate	Mosaferi, E.; Ripsman, D.; Stephan, D. W., *Chemical Communications* **2016,** *52* (53), 8291-8293.	10.1039/C6CC03970G
$[Ir(COD)Cl]_2$	Choudhury, J.; Podder, S.; Roy, S., *Journal of the American Chemical Society* **2005,** *127* (17), 6162-6163.	10.1021/ja0506004

Materials from chemical suppliers:

1. **Diethyl 2,6-dimethylpyridine-3,5-dicarboxylate** (Aldrich catalog #120251). Used as received.
2. **Lithium Aluminum Hydride Solution (1 M in THF)** (Aldrich catalog # 212776). Used as received
3. **Tetrahydrofuran** (Aldrich catalog # 401757). Used as received.
4. **Methanol**. (VWR catalog # BDH1135)
5. **Acetonitrile**. (Aldrich catalog # 271004). Bubbled with argon.
6. **Phosphorus tribromide** (Aldrich catalog # 256536). Used as received.
7. **Anhydrous dimethylformamide** (Aldrich catalog # 227056). Used as received.
8. **Di-tert-butylphosphine.** (Strem catalog # 15-1040). Used as received.
9. **Anhydrous triethylamine** (Aldrich catalog # 471283). Used as received.
10. **Anhydrous benzene** (Aldrich catalog # 401765). Bubbled with argon.
11. **Anhydrous Toluene** (Aldrich catalog#244511). Bubbled with argon.
12. **Benzoquinone**. (Aldrich catalog#B10358). Purified by sublimation.
13. **Trityl BArF^{20}**. (Strem catalog # 05-5000). Used as received.
14. **Methylene chloride-d_2**. (Cambridge Isotope Labs Catalog # DLM-23-10). Used as received.
15. **Chloroform-d.** (Cambridge Isotope Labs Catalog # DLM-7-100). Passed through a plug of anhydrous sodium sulfate.
16. **Benzene-d_6.** (Cambridge Isotope Labs Catalog # DLM-1-100). Degassed by iterative freeze-pump-thaw cycles and stored over molecular sieves inside glovebox.
17. **DMSO-d_6**. (Cambridge Isotope Labs Catalog # DLM-10-10). Used as received.
18. **Ferrocenium hexafluorophosphate** (Aldrich Catalog # 388297). Used as received.
19. **2,6-Lutidine.** (Aldrich catalog # L3900) Distilled by bulb to bulb distillation, degassed, and stored over molecular sieves inside a glovebox.

Reactions of 2-H in Table 1:

General comments: Handling of all materials was done inside an argon-filled glovebox. Reactions were performed at room temperature. Unless otherwise noted, where reactions occurred, they did so immediately upon mixing.

Entry 1: $[Cp_2Fe^+][PF_6^-]$ (1 eq.) (*Scheme 4)*

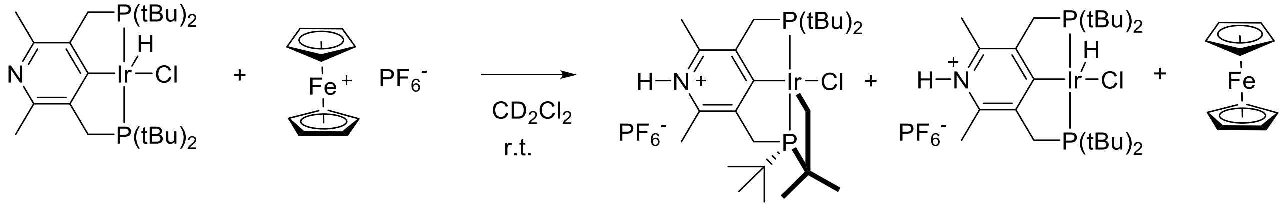

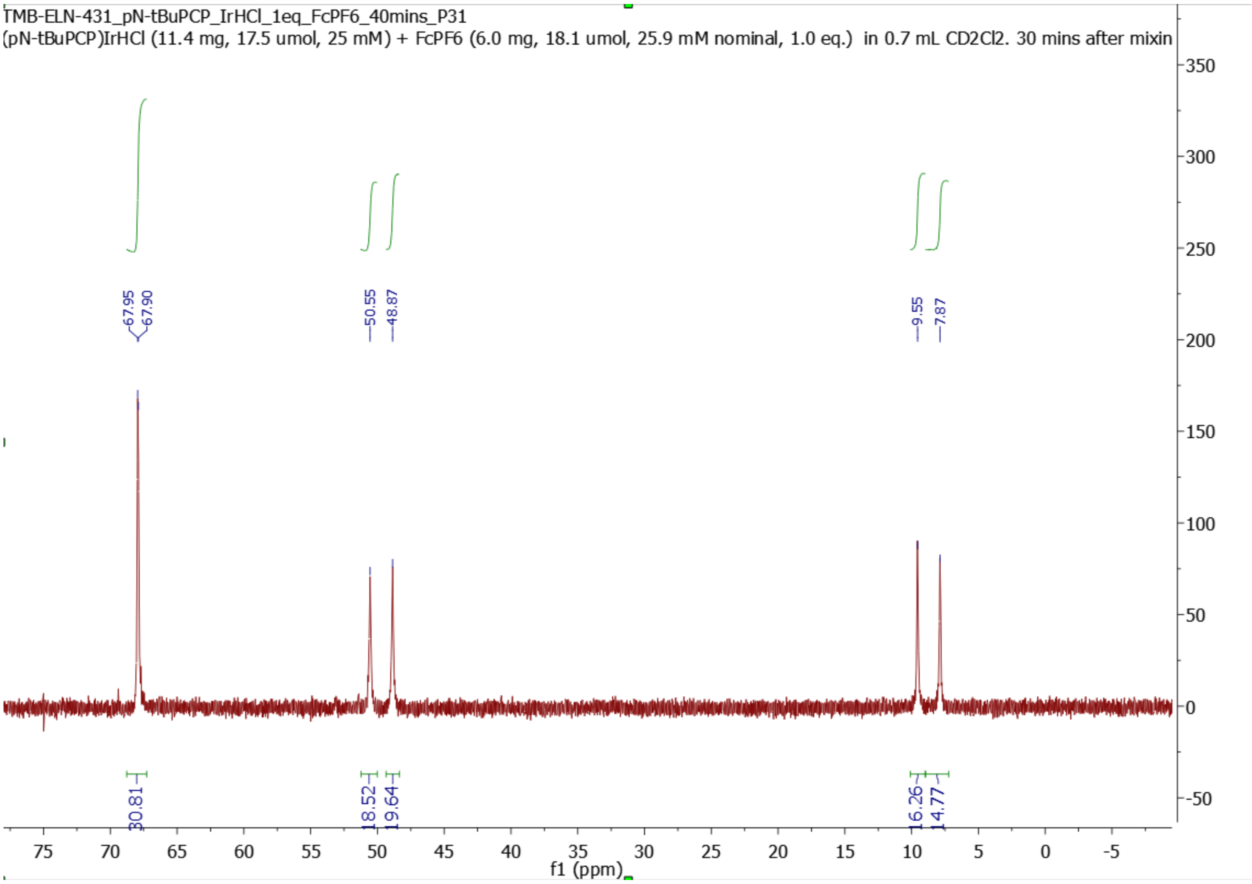

Fig. S15: ^{31}*P-NMR for Entry 4. Identified species:* ***H⁺-2-H*** *(30.8%, 7.7 mM) and* ***H⁺-4*** *(69.2%, 17.3 mM).*

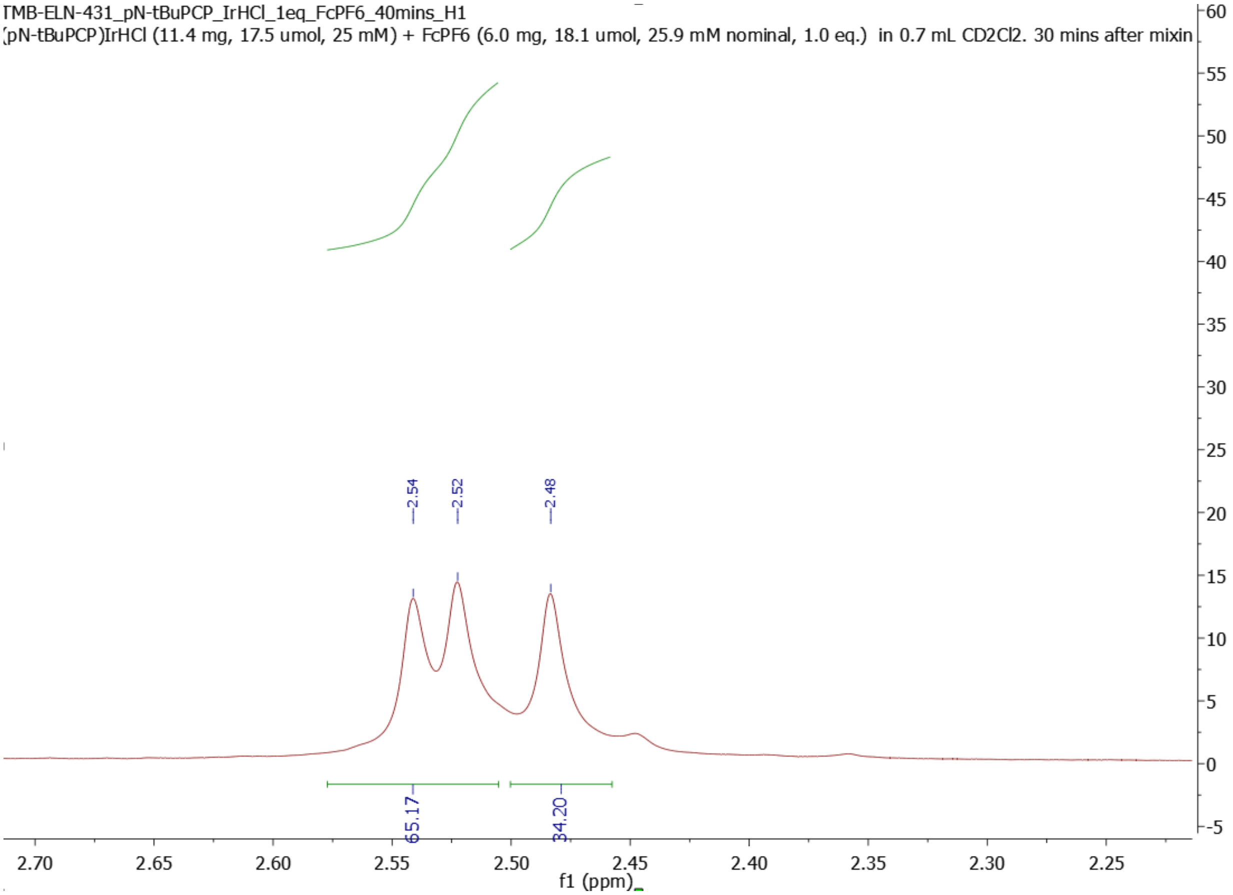

Fig. S16: [1]*H-NMR for Entry 4, expansion of α-methyl group signals which were used for quantitation. Identified species:* ***H^+-2-H*** *(34.2%, 8.6 mM) and* ***H^+-4*** *(65.2%, 16.3 mM).*

Entry 2: $[Cp_2Fe^+][PF_6^-]$ (2 eq.)

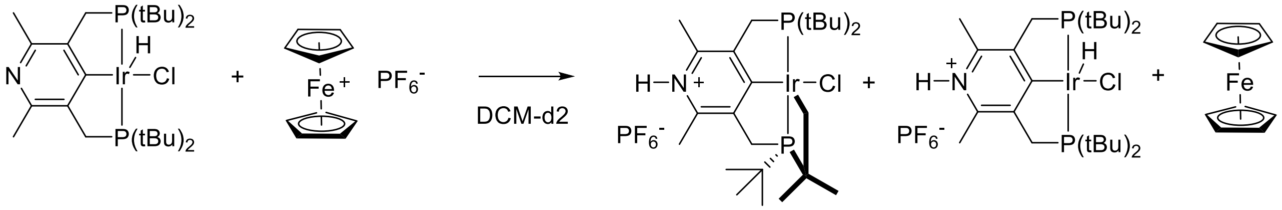

(pN-tBuPCP)IrHCl (6.5 mg, 10 umol) in 0.75 mL CD_2Cl_2 was poured into a 1 dram vial containing FcPF6 (6.5 mg, 19.6 umol), and agitated with a pipette to mix. Solution darkens upon mixing. Reaction mixture is transferred to a J. Young NMR tube for analysis.

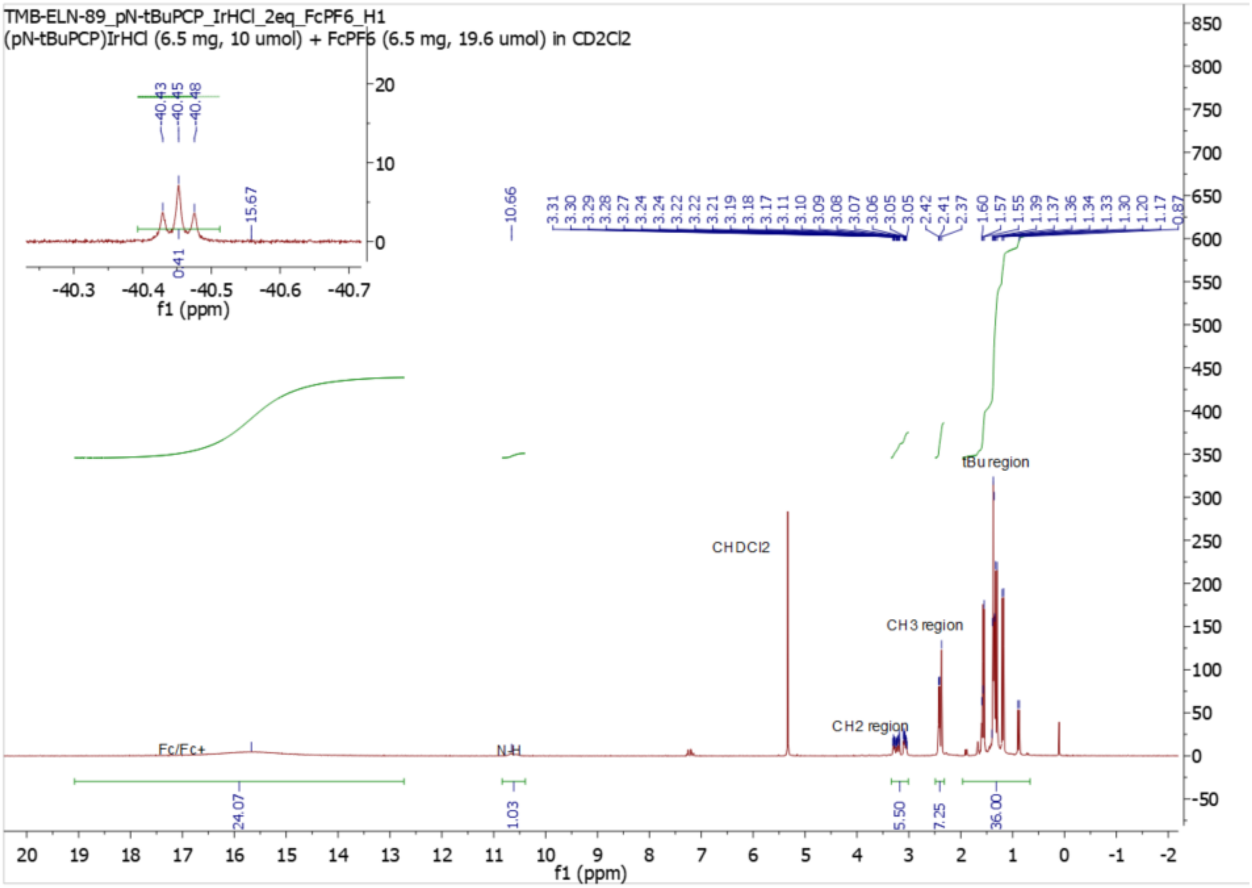

Fig. S17. *^1H NMR spectrum after reaction.*

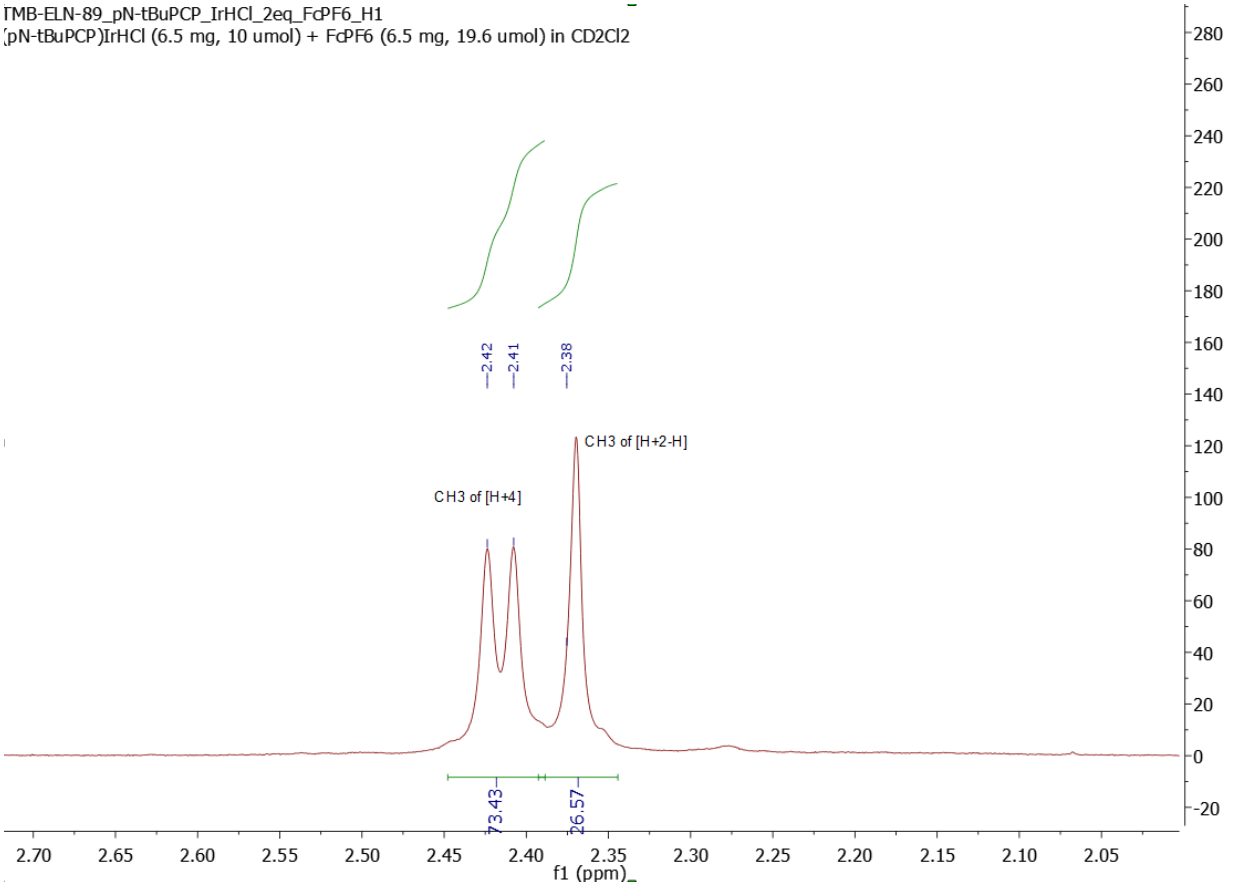

*Fig. S18: Portion of ^{1}H-NMR spectrum used to quantify **H⁺4** (73.4%, 9.8 mM) and **H⁺2-H** (25.6%, 3.5 mM)*

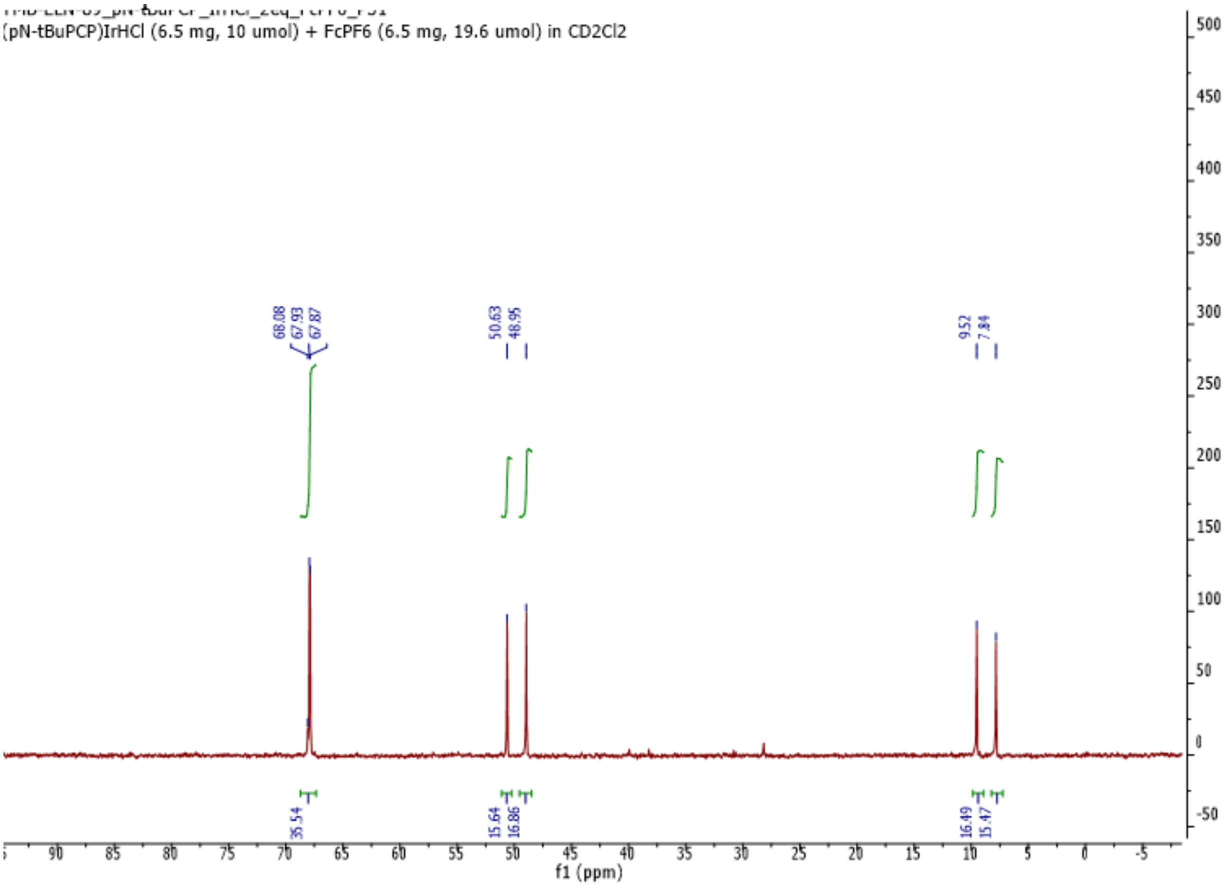

Fig. S19: ^{31}P NMR spectrum of mixture after reaction.

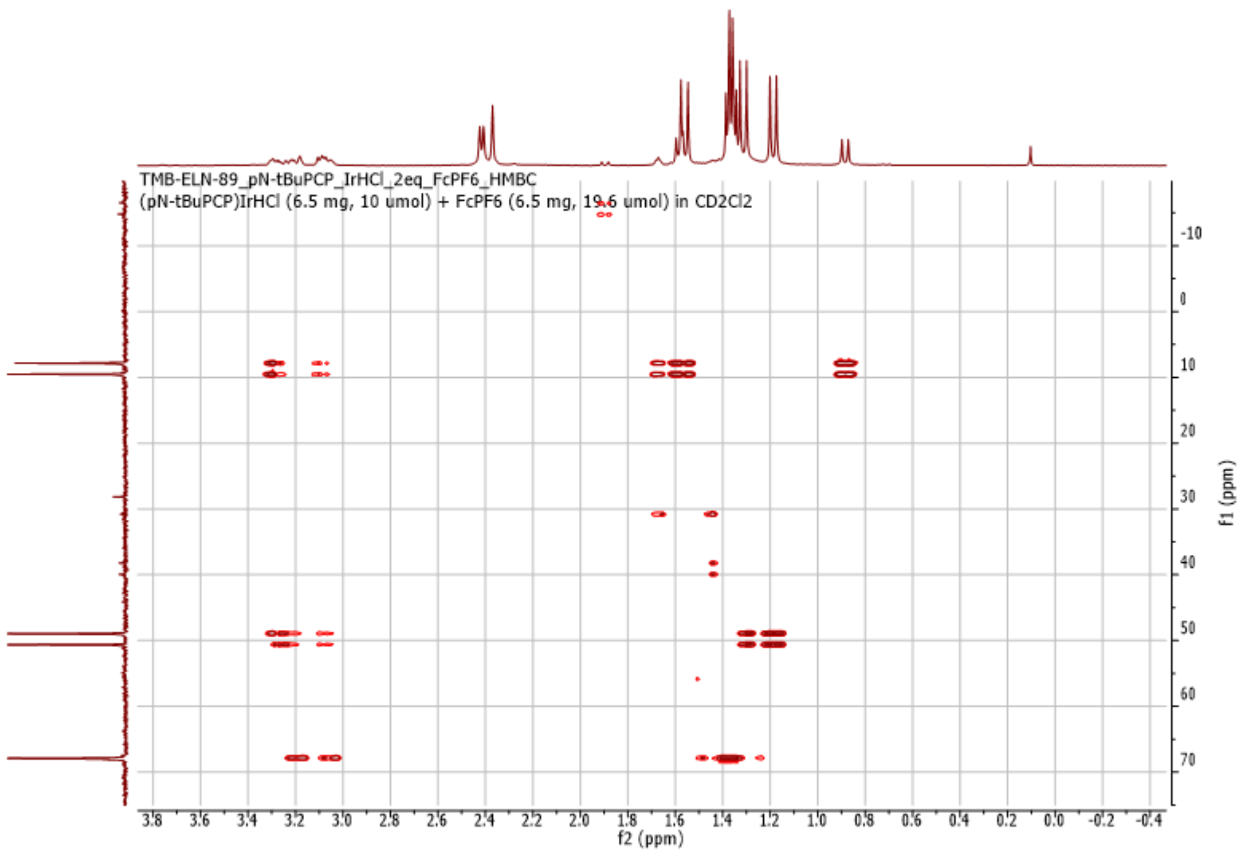

Fig. S20: *^{31}P-^{1}H HMBC spectrum (8 Hz filter) of mixture after reaction. Used to characterize* ***H^{+}4.***

Entry 3: [Cp_2Fe^+][PF_6^-] (2 eq.) + 2,6-lutidine (9 eq.) (*Scheme 5*)

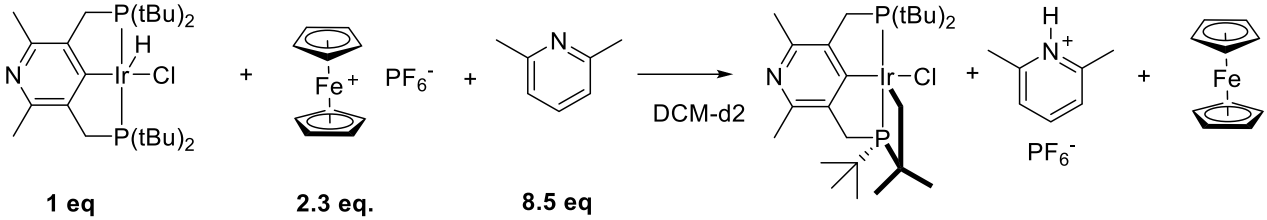

2-H (5.7 mg, 8.8 µmol) dissolved in 0.7 mL CD_2Cl_2 was poured into a 1 dram vial containing ferrocenium tetrafluoroborate (6.7 mg, 20.3 µmol) and 2,6-lutidine (8 mg, 74.8 µmol). The solution darkens upon mixing, and then lightens to amber/brown with black heterogeneous solids. ^{31}P-NMR (below) shows near complete cyclometallation, along with two unidentified signals compromising ~10% of the total ^{31}P integral.

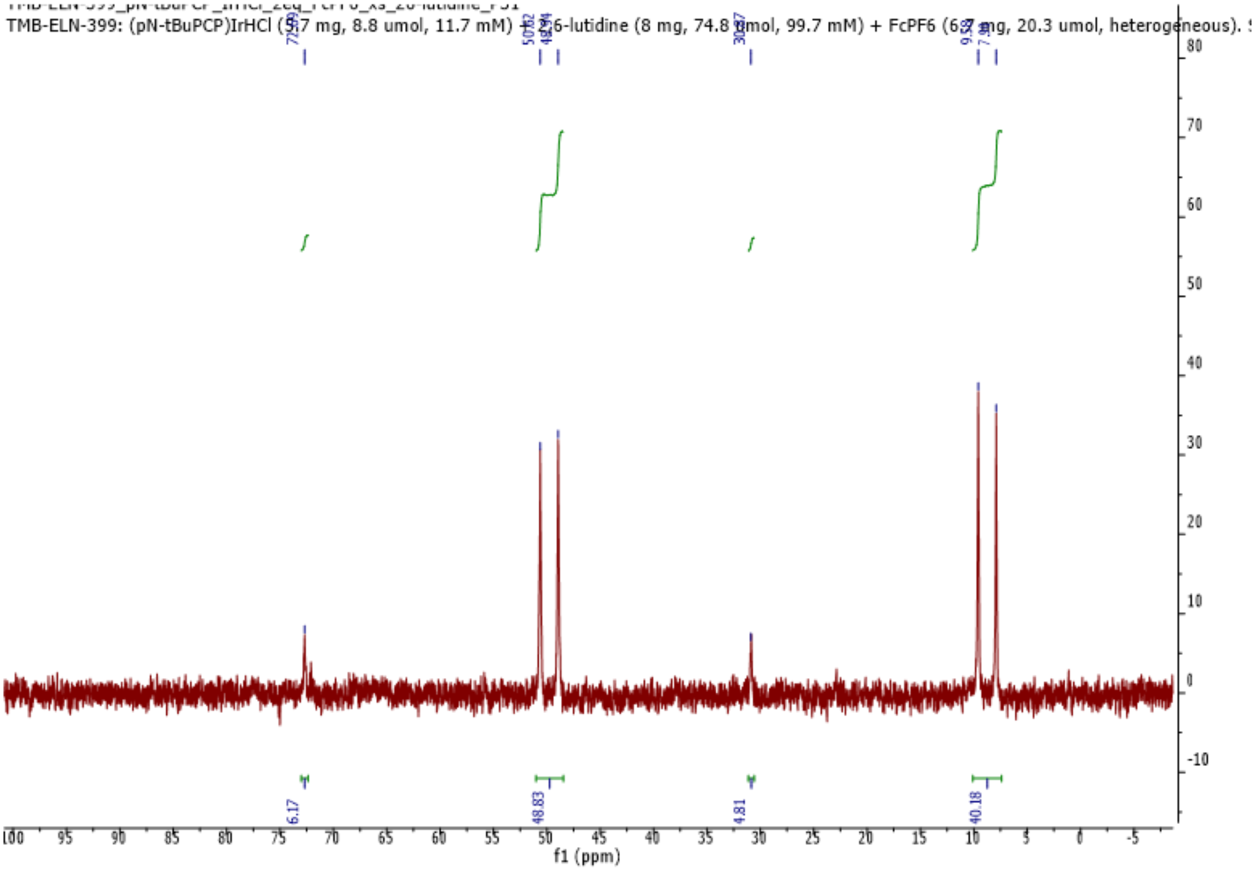

*Fig. S21: ^{31}P-NMR after a reaction of **2-H** + 2 eq. $FcPF_6$ in CD_2Cl_2.*

Entry 4: Trityl BArF[20] (1 eq.) (*Scheme 6*)

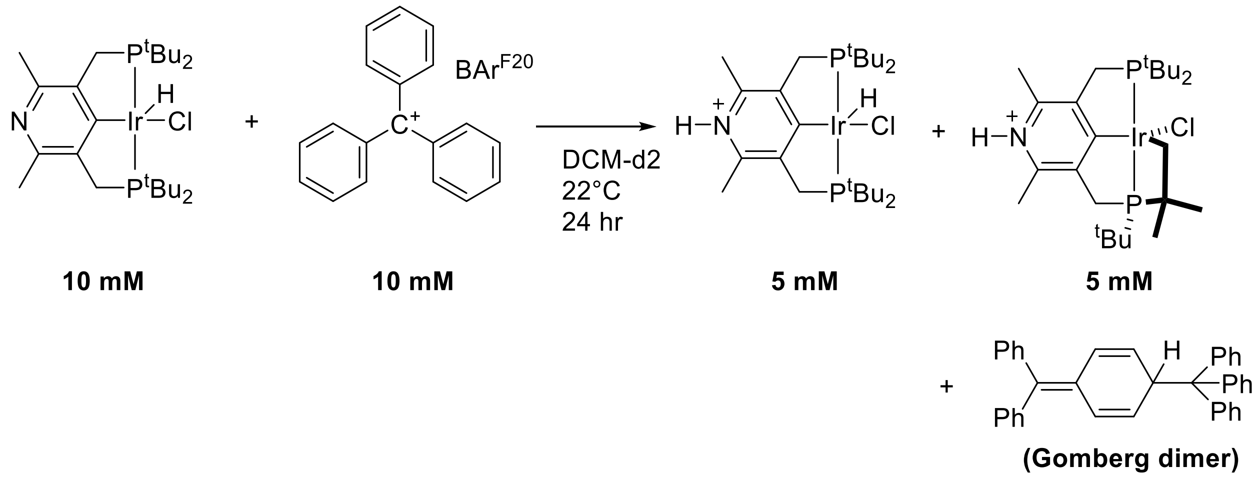

Premade solutions of trityl BAr^{F20} and (pN-^{tBu}PCP)IrHCl in DCM-d_2 were combined in the appropriate amounts to give a 10 mM solution of each inside a J. Young NMR tube. ^{1}H NMR and ^{31}P NMR spectra were acquired 45 minutes later.

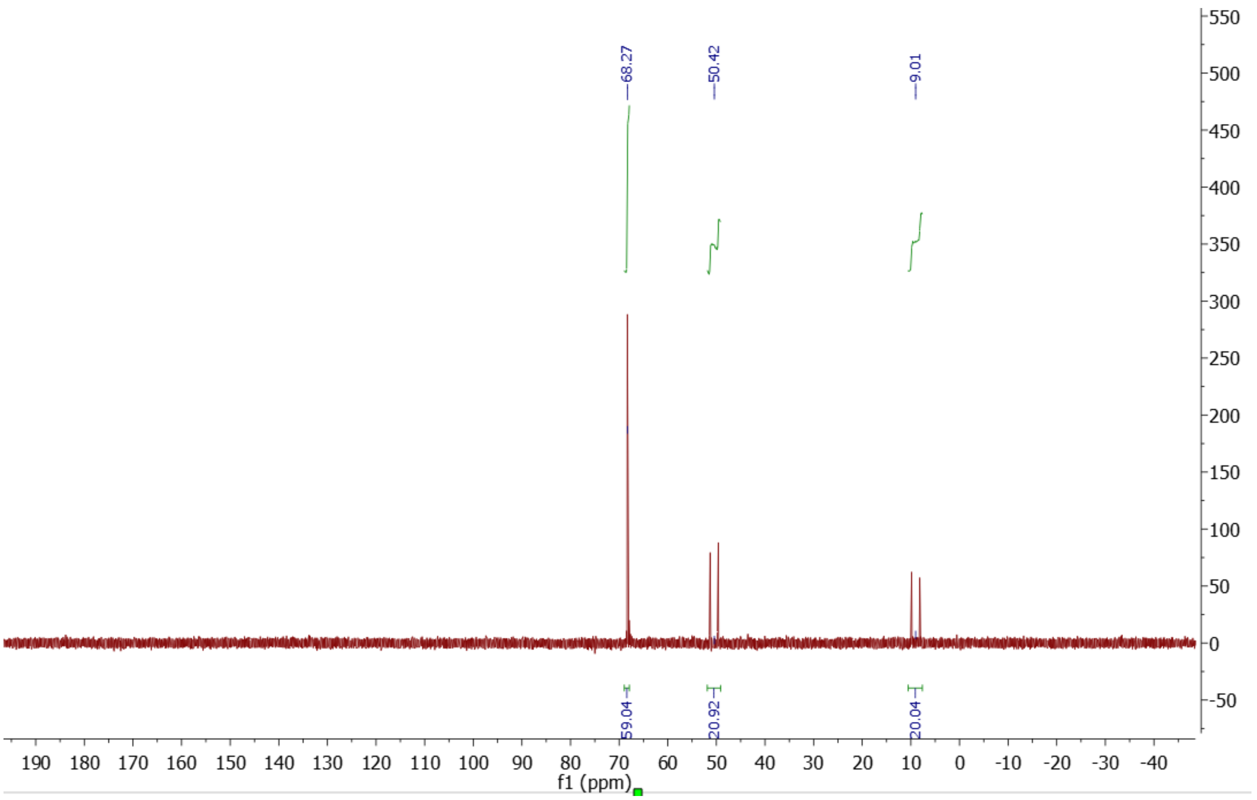

*Fig. S22: ^{31}P-NMR spectrum for entry 4. Identified species: **H$^+$-2-H** (5.9 mM, 59%) and **H$^+$-4** (4.1 mM, 41%)*

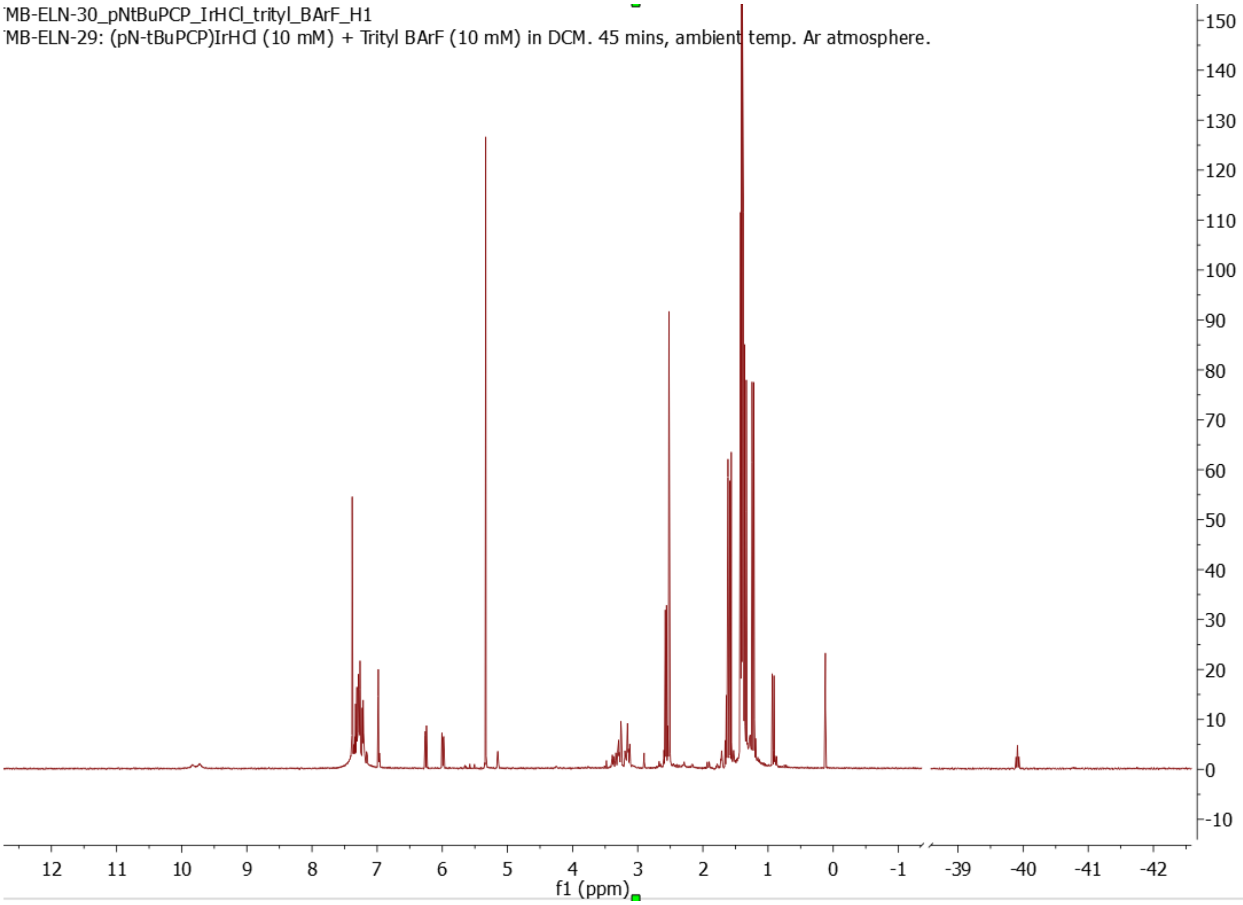

*Fig. S23: ^{1}H-NMR spectrum for entry 4. Identified species: **H^+-2-H** (5.9 mM, 59%) and **H^+-4** (4.1 mM, 41%). Wide view. Region between -1 ppm and -39 ppm is clipped (no signals present).*

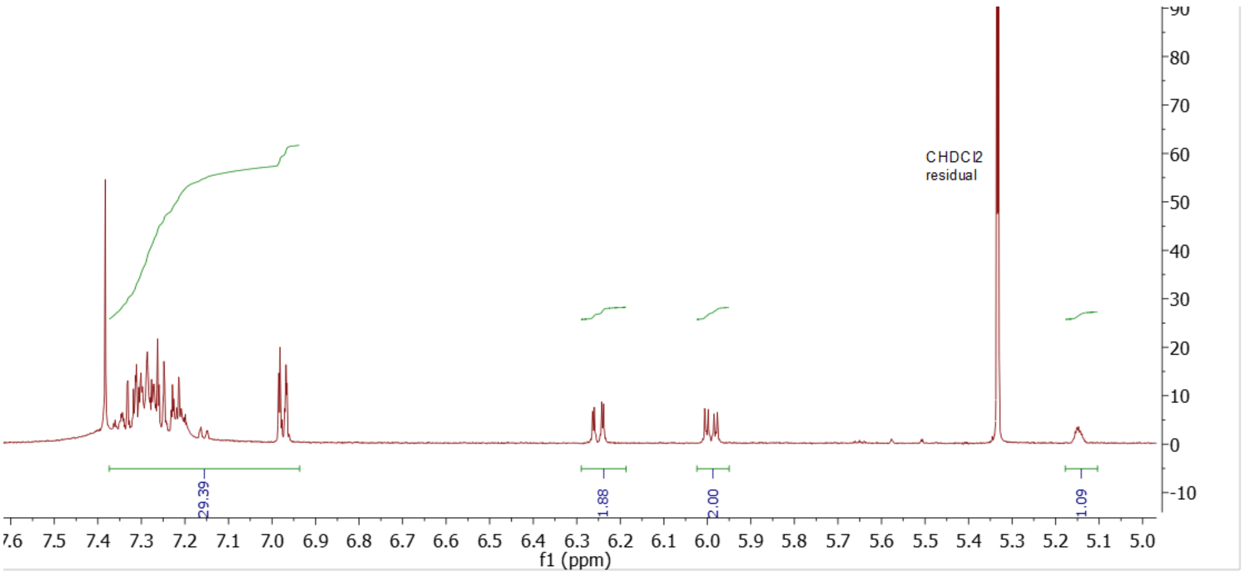

Fig. S24: Signals for Gomberg's dimer in ^{1}H-NMR.

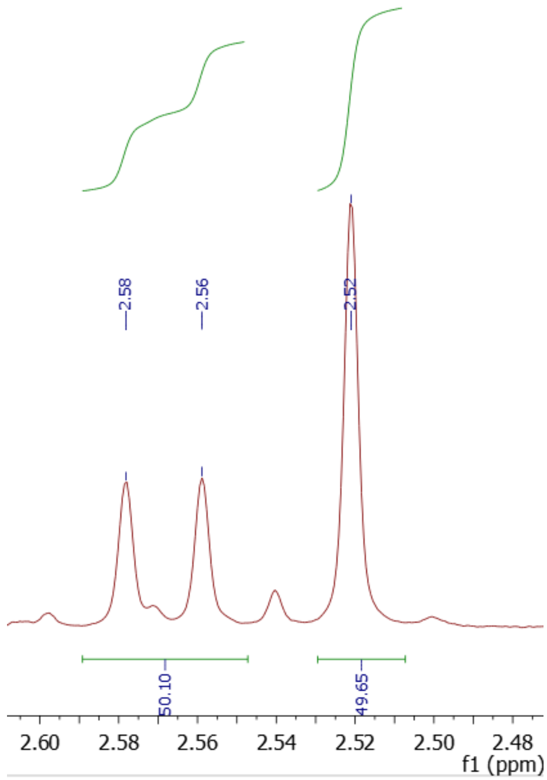

*Fig. S25: Integrals of methyl groups **[H+2-H]**[BArF] and **[H+4]**[BArF] products was used to determine their relative concentrations. No starting material remains.*

Entries 5 and 6: Benzoquinone (1 eq.), 40 mins and 48 hours

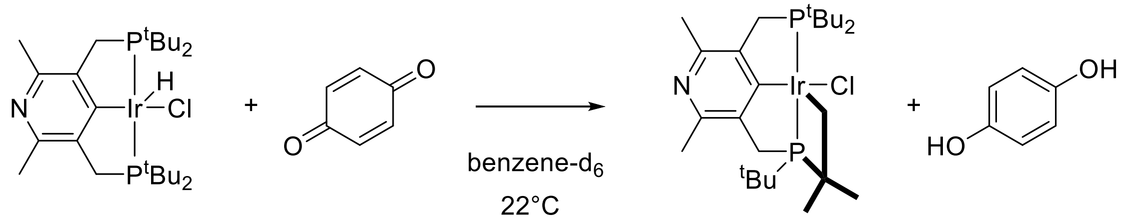

7 mM   7 mM

Solutions of **2-H** and benzoquinone were prepared separately in benzene-d$_6$, then combined under an argon atmosphere inside a glovebox. The combined mixture was transferred to a J. Young NMR tube and analyzed by ^{1}H-NMR after 40 minutes and 48 hours.

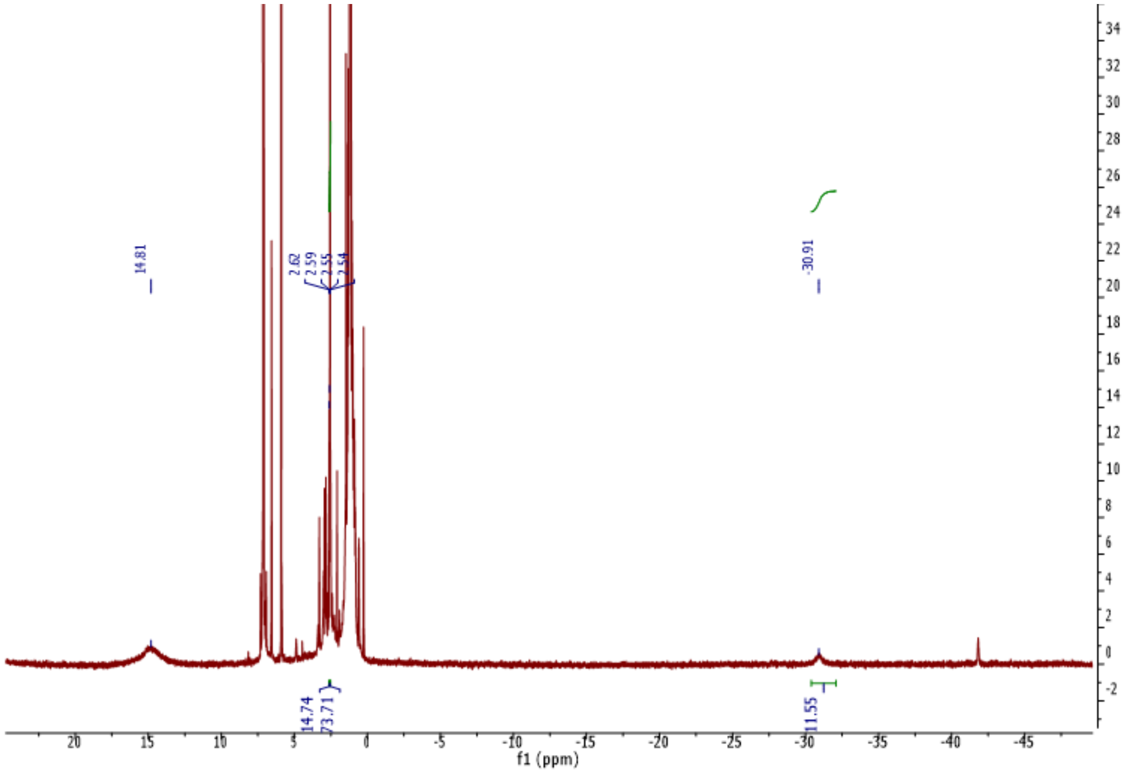

Fig. S26: ^{1}*H-NMR of* ***2-H*** *(7 mM, 1 eq.) + benzoquinone (7 mM, 1 eq.), 40 minutes, room temperature, in benzene-d$_6$. Identified species:* ***4*** *(1.0 mM, 15%),* ***2-H*** *(5.1 mM, 74%),* ***2*** *(0.8 mM, 11%).*

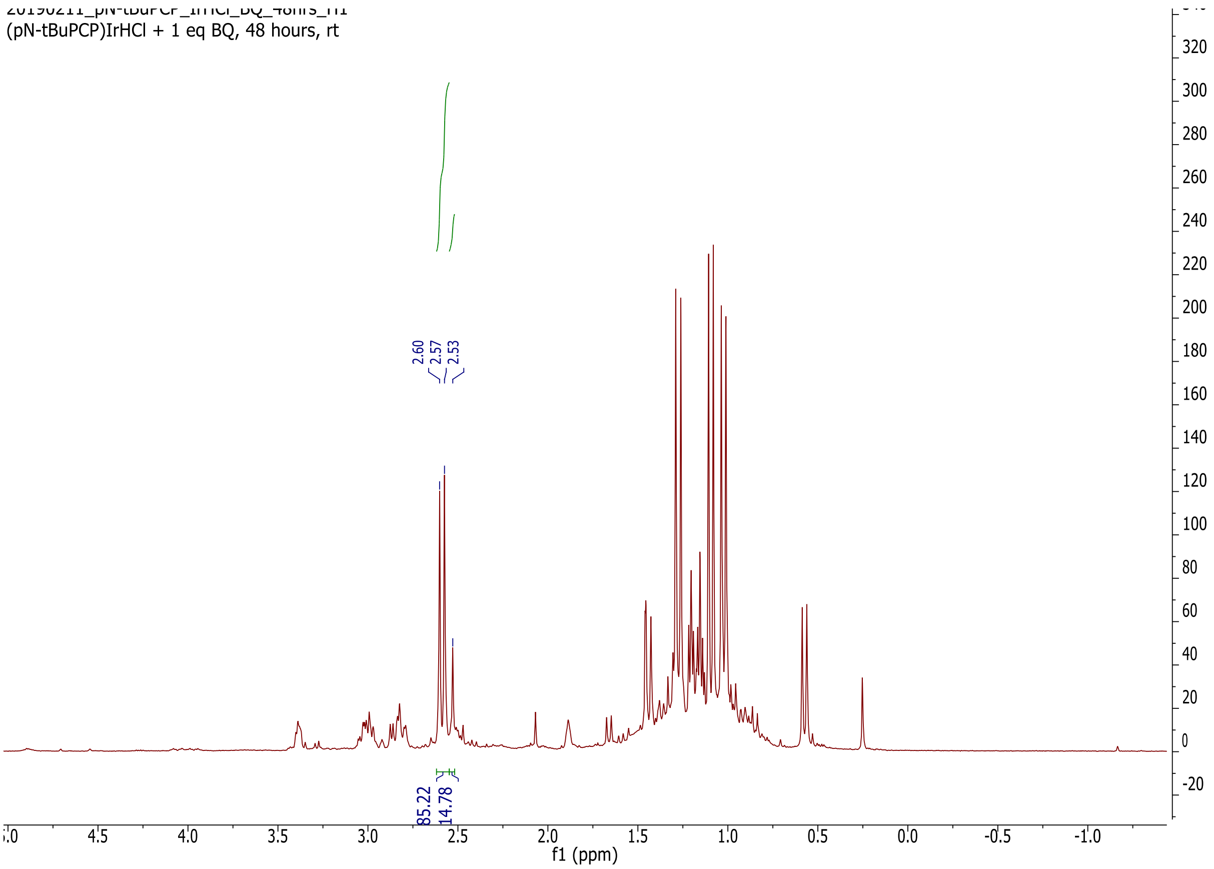

Fig. S27: ^{1}*H-NMR of* ***2-H*** *(7 mM, 1 eq.) + benzoquinone (7 mM, 1 eq.), 48 hours, room temperature, in benzene-d*$_6$*. Identified species:* ***4*** *(5.9 mM, 85%),* ***2-H*** *(1.0 mM, 15%).*

Entry 7: [(*p*-MeO-C_6H_4)$_3C^+$][BF_4^-] (3 eq.)

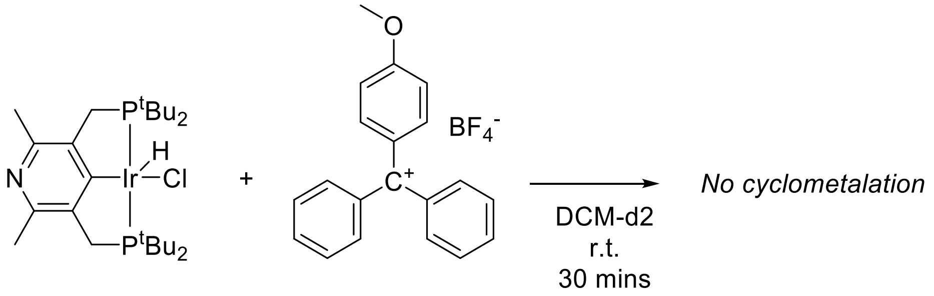

17.3 mM **50 mM**

2-H (7.9 mg, 12.1 µmol) was dissolved into 0.7 mL dichloromethane-d2 inside a 1 dram vial. This solution was transferred to a J. Young tube for NMR analysis. This solution was then mixed with methoxytrityl tetrafluoroborate (12.6 mg, 35 µmol, 2.9 eq.), and analyzed again by NMR. No apparent reaction ensues.

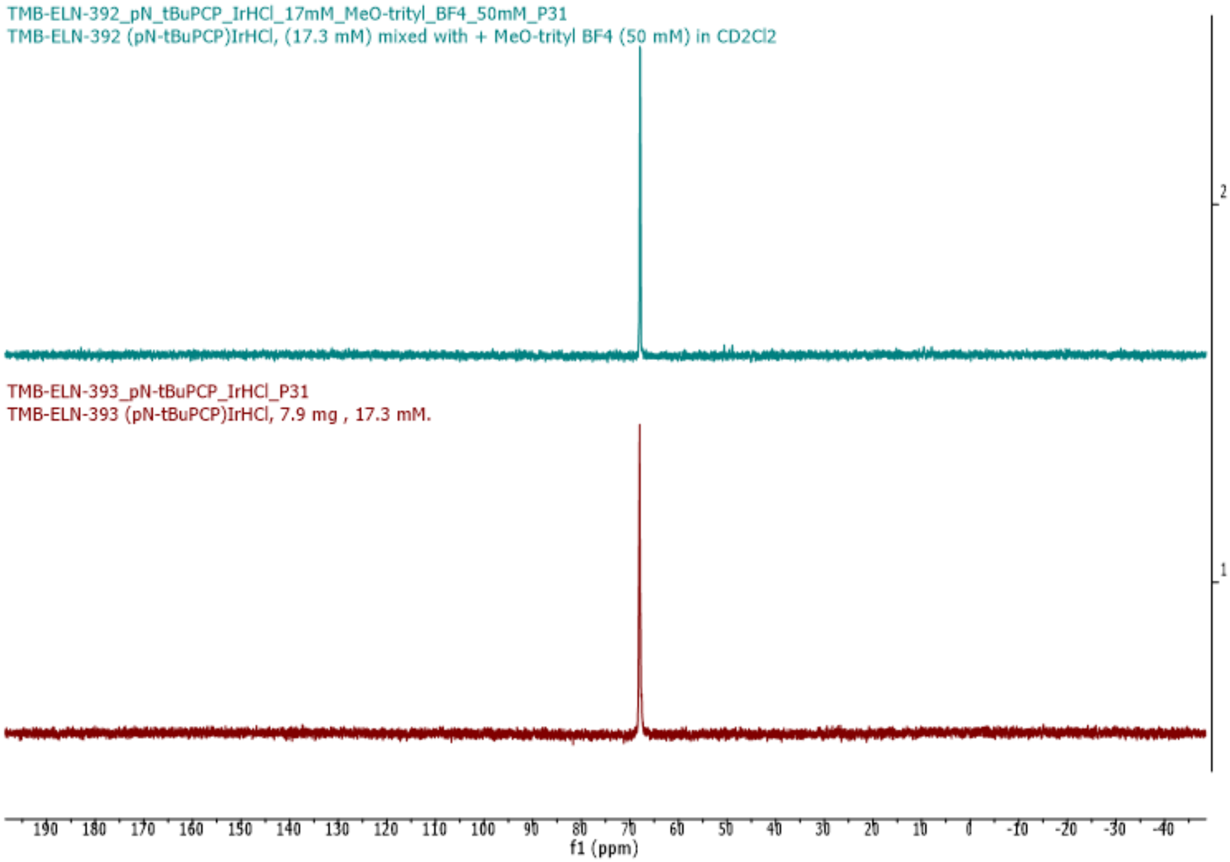

*Fig. S28: ^{31}P-NMR data. Bottom: **2-H** before mixing with methoxytrityl tetrafluoroborate. Top: after mixing.*

Entry 8: [Cp*$_2$Fe$^+$][BF$_4^-$] (2 eq.)

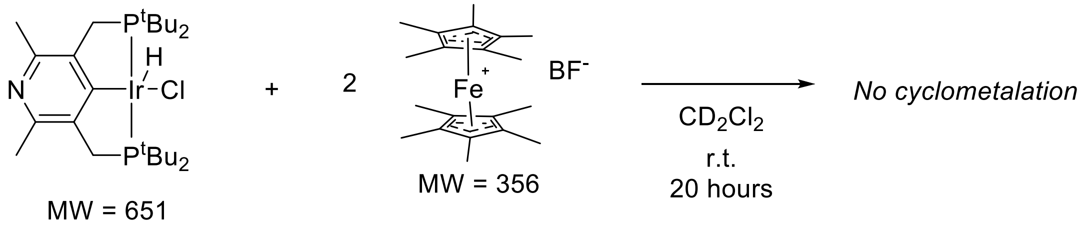

2-H (5.4 mg, 8.29 µmol) and decamethylferrocenium tetrafluoroborate (7.6 mg, 18.4 µmol, 2.22 eq.) were weighed into separate 1 dram vials. CD_2Cl_2 (0.75 mL) was added to the vial containing **2-H**, agitated with a pipette to fully dissolve, and then the resulting orange-red solution was transferred to a J. Young NMR tube. NMR spectra were recorded. This solution was then poured into the vial containing decamethylferrocenium tetrafluoroborate, the mixture was agitated with a pipette to fully dissolve, and then the solution was transferred back to the J. Young NMR tube. The sealed solution was allowed to stand inside the glovebox for 20 hours before another set of NMR spectra were recorded.

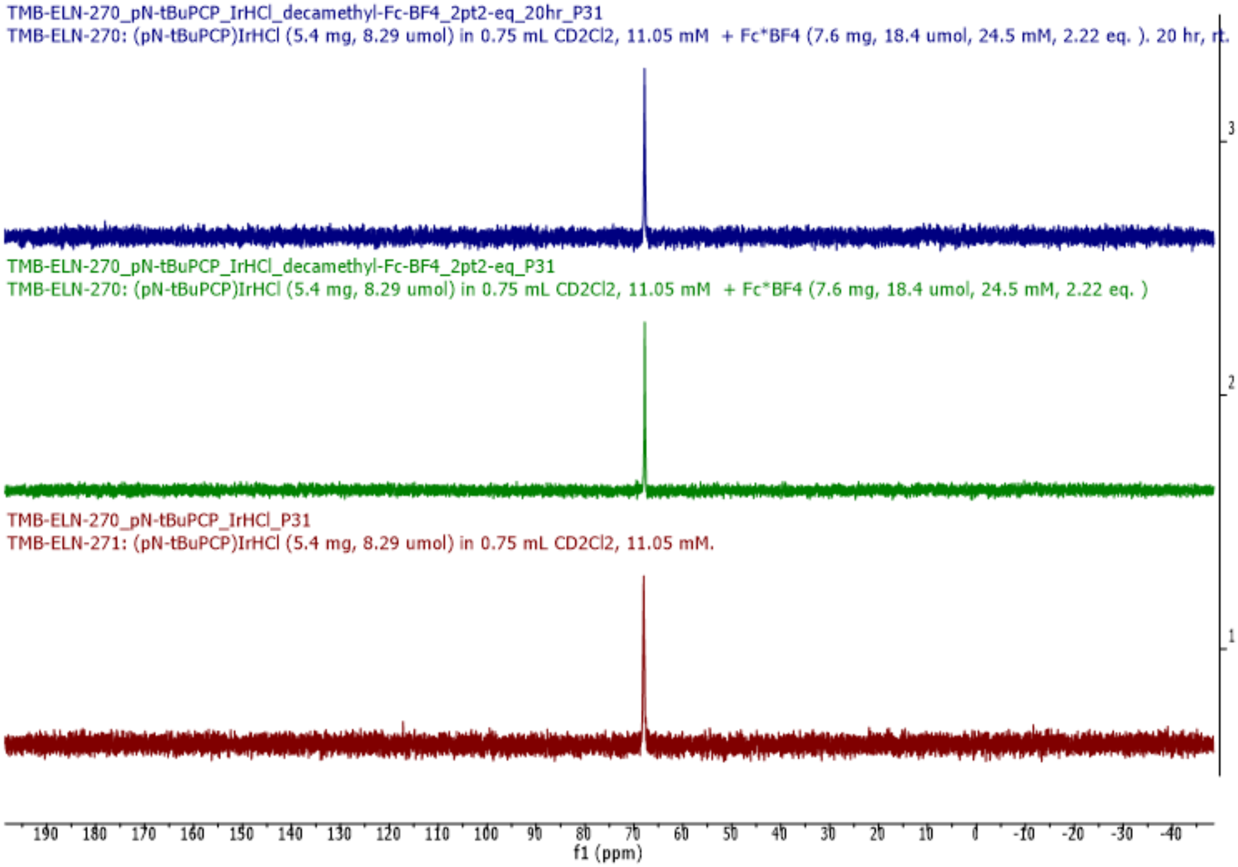

*Fig. S29: ^{31}P-NMR data. Bottom (red trace): **2-H** before contacting Fc*BF$_4$. Middle (green trace): immediately after contacting Fc*BF$_4$. Top (blue trace): After 20 hours of contacting Fc*BF$_4$.*

Observations of iridium(II) intermediate 2•

Reaction of 2-H (7 mM) with benzoquinone (540 mM)

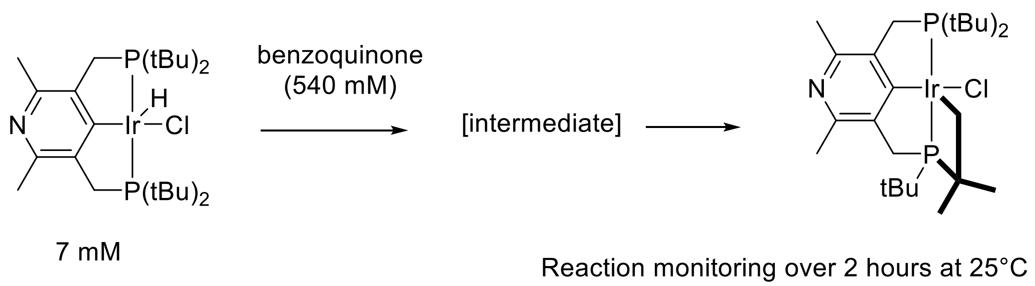

Inside an argon-filled glovebox, complex **2-H** (3 mg, 4.6 µmol) was weighed directly into a J. Young NMR tube and sealed with a rubber septum. Separately, a solution of benzoquinone (40 mg, 378 µmol) was prepared in 700 µL benzene-d_6 in a 1-dram vial, and sealed with a rubber septum. At the instrument, a 1-mL Hamilton syringe was used to pierce the septum of the 1 dram vial, draw out the benzoquinone solution, and inject it into the J. Young NMR tube containing **2-H**. The top of the J. Young NMR tube and the rubber septum were quickly covered with parafilm, the mixture tapped to homogenize, and the tube was inserted into the NMR. Locking and shimming took 100 seconds.

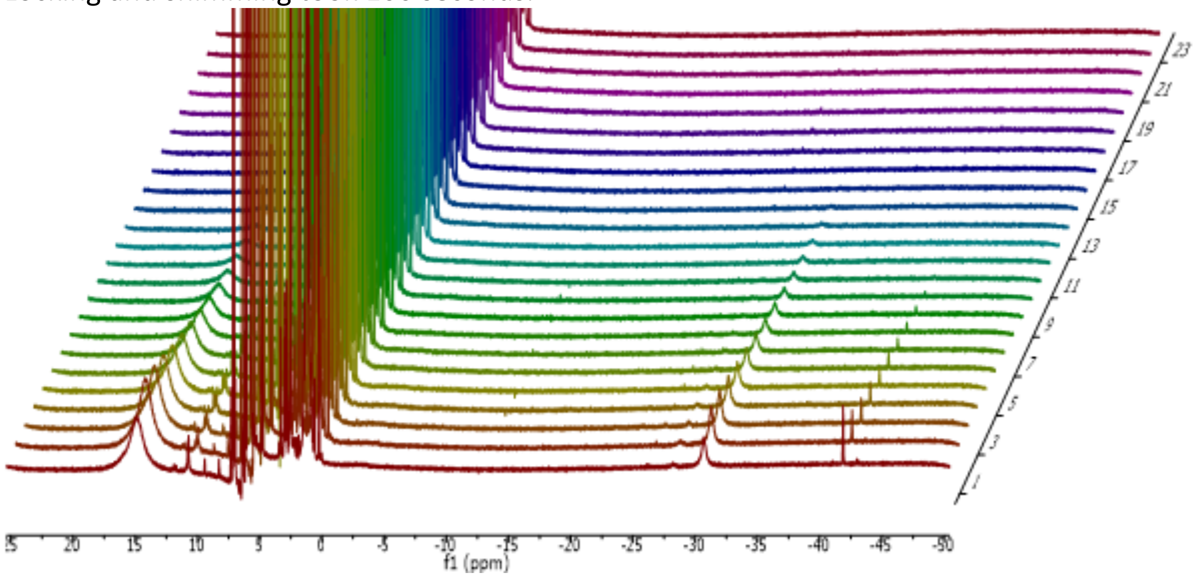

*Fig. S30: Reaction of **2-H** in excess benzoquinone was monitored with one acquisition every 5 minutes for 2 hours (total 24 scans). Starting material **2-H** rapidly decays (see hydride signal at -41.8 ppm) with concomitant growth of an intermediate (signals at +15 ppm and -30 ppm), followed by slower decay of both.*

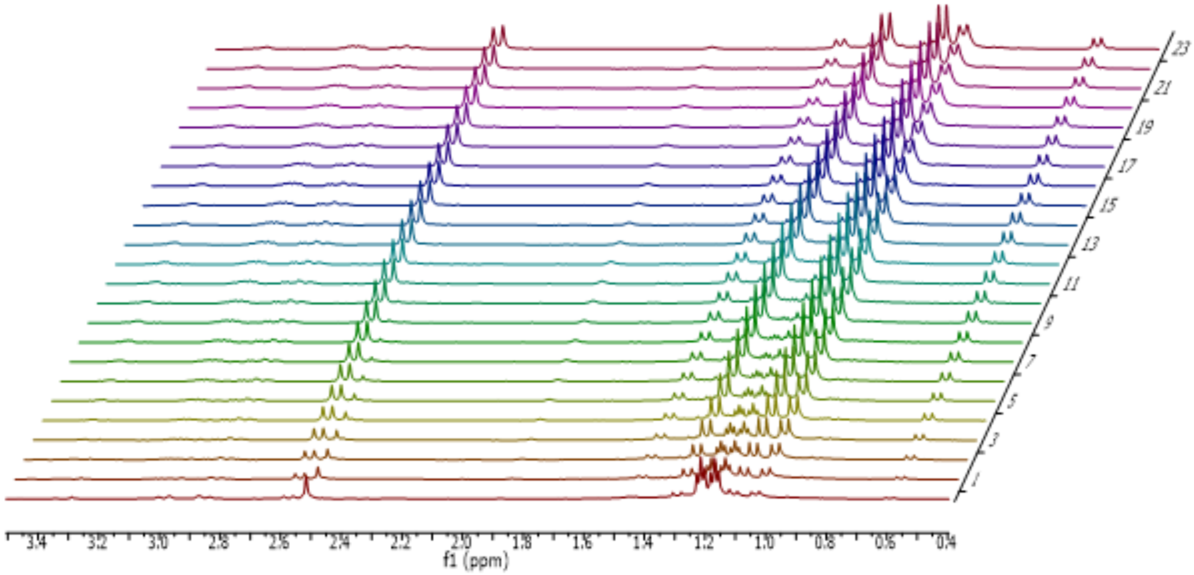

*Fig. S31: Reaction of **2-H** in excess benzoquinone was monitored with one acquisition every 5 minutes for 2 hours (total 24 scans). Growth of unsymmetrical signals attributable to cyclometalated complex **4** are seen as the reaction progresses, and at later times this is the only distinguishable iridium complex in solution.*

Quenching iridium(II) intermediate with TEMPO-H to return 2-H

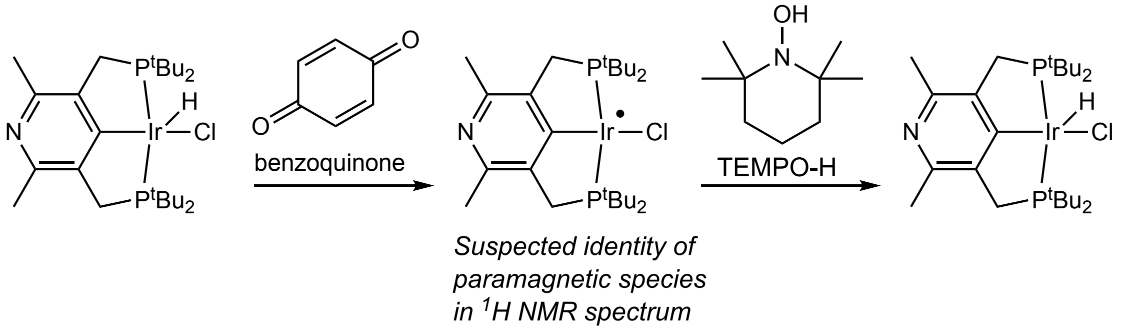

Inside an argon-filled glovebox, **2-H** (7.8 mg, 12 umol, 19.8 mM) was dissolved in benzene-d_6 (0.6 mL) and transferred into a J-Young NMR tube. The NMR tube was sealed with a rubber septum. A ^{1}H NMR spectrum was acquired. Then, benzoquinone was added (50 uL, 185 mM in benzene-d_6, 9.25 umol, 1.5 eq) by syringe through the septum. This mixture was allowed to react for 5 minutes inside the NMR (25 °C) before a spectrum was recorded. Then, 50 uL of 640 mM TEMPO-H (32 µmol, 2.7 eq) was injected through the septum by syringe. Immediate precipitates (presumable hydroquinone) form. Final NMR is taken.

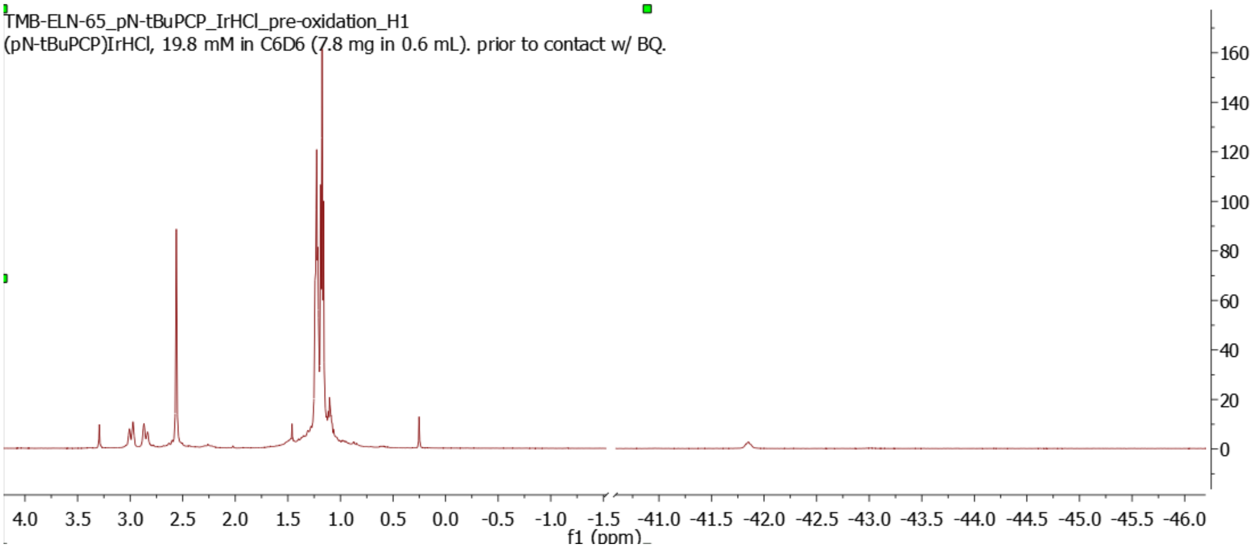

*Fig. S32: **2-H** (19.8 mM in C6D6) before mixing with benzoquinone.*

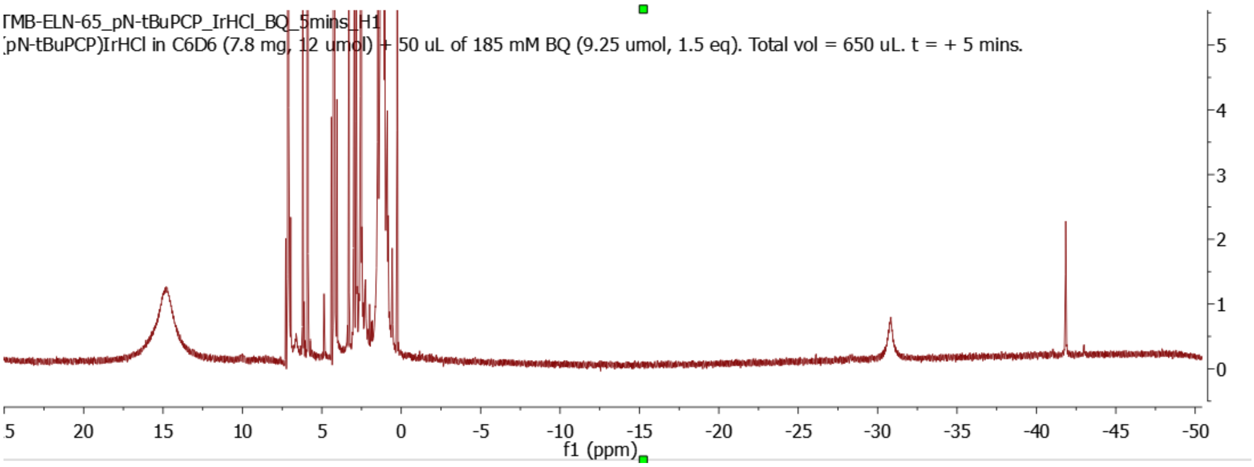

Fig. S33. ***2-H*** *+ 1.5 eq. benzoquinone, after 5 minutes of reaction at 25 °C. Signals attributable to* ***2•*** *have appeared at +15 ppm and -31 ppm.*

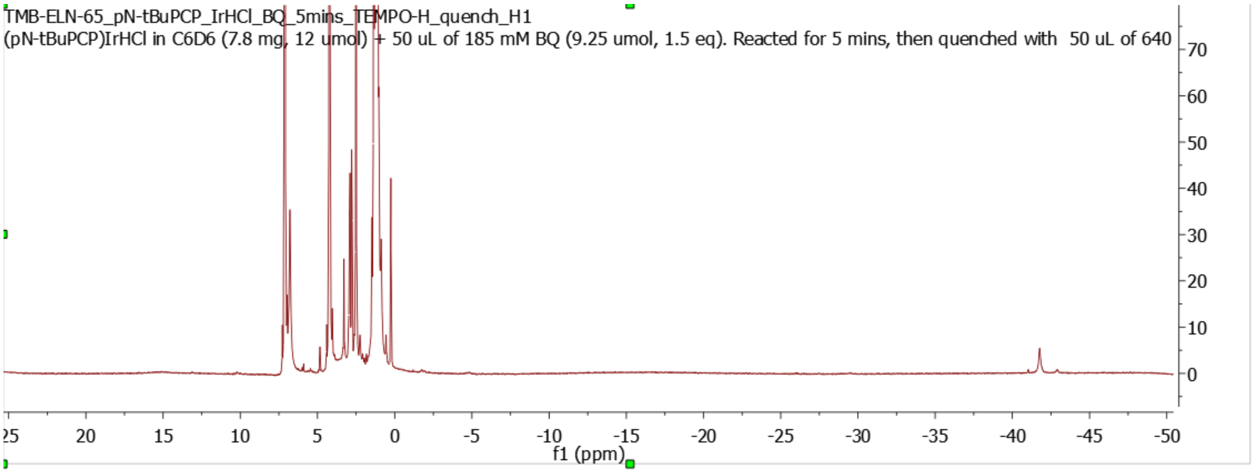

Fig. S34. ***2-H*** *+ 1.5 eq. benzoquinone, after 5 minutes of reaction at 25 °C and then addition of TEMPO-H. Signals of* ***2•*** *have completely disappeared. Only remaining signals for iridium complex are attributable to* ***2-H****.*

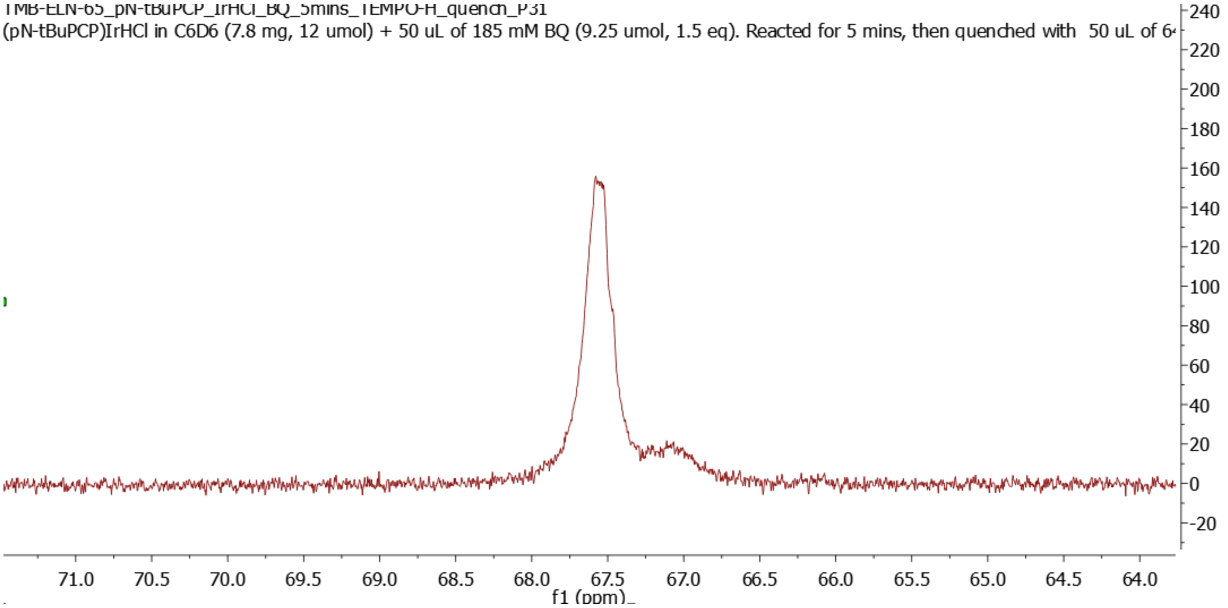

Fig. S35. ***2-H*** *+ 1.5 eq. benzoquinone, after 5 minutes of reaction at 25°C and then addition of TEMPO-H.* ^{31}*P-NMR spectrum showing signal attributable to* ***2-H*** *after reaction*

Scheme 9. Reaction of 2-H with 2,4,6-tri-*tert*-butylphenoxy radical.

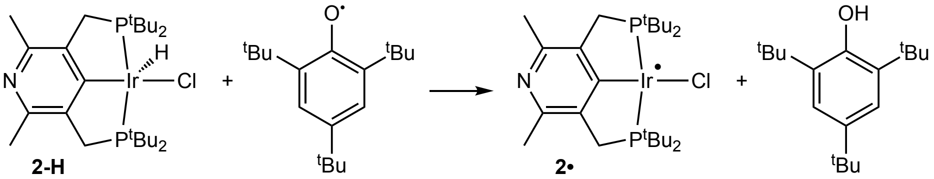

2-H (6.6 mg, 10.1 μmol, 20 mM) was weighed into a 1 dram vial and dissolved into 500 μL benzene-d_6. An NMR spectrum of this solution was recorded. This solution was pipetted into a second 1 dram vial containing tri-*tert*-butylphenoxy radical (2.6 mg, 10.1 μmol, 20 mM, 1 eq.), agitated with a pipette to dissolve, then transferred to a J. Young NMR tube and quickly frozen in liquid nitrogen to transport to the NMR. At the instrument, the solution was thawed and inserted. Locking and shimming took 2 minutes. The reaction was monitored over the next 30 minutes at 25°C, with one spectrum recorded every 30 seconds. The appearance of paramagnetic **2•** is seen, both its concentration and that of **2-H** remain constant over this period. After monitoring for 30 minutes, a final spectrum is recorded.

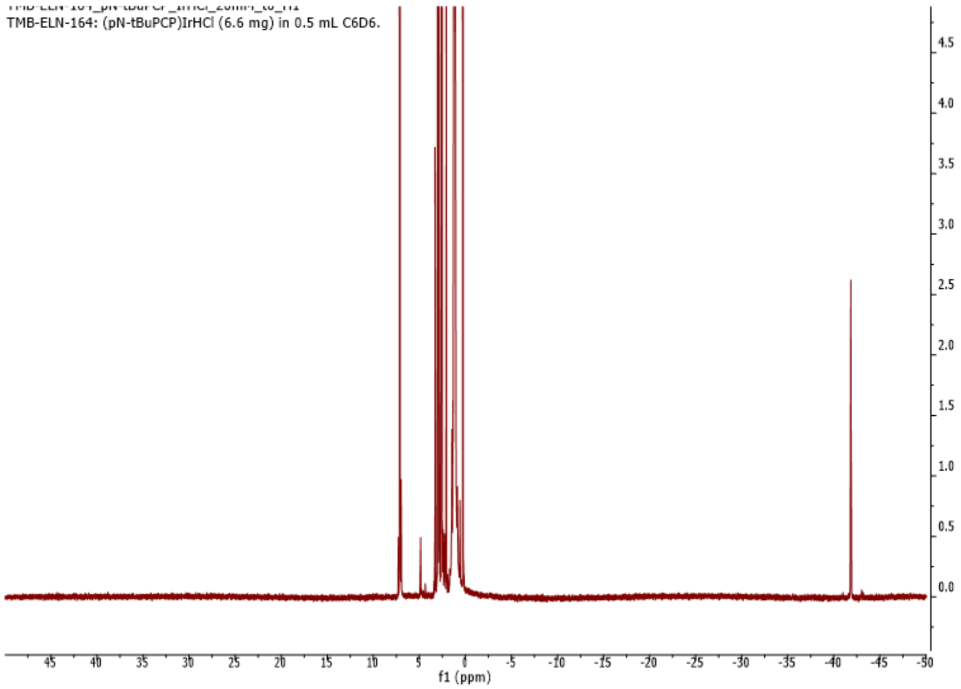

*Fig. S36. 20 mM **2-H** in benzene-d_6, before contacting tri-tert-butylphenoxy radical. No signals attributable to **2•** are visible.*

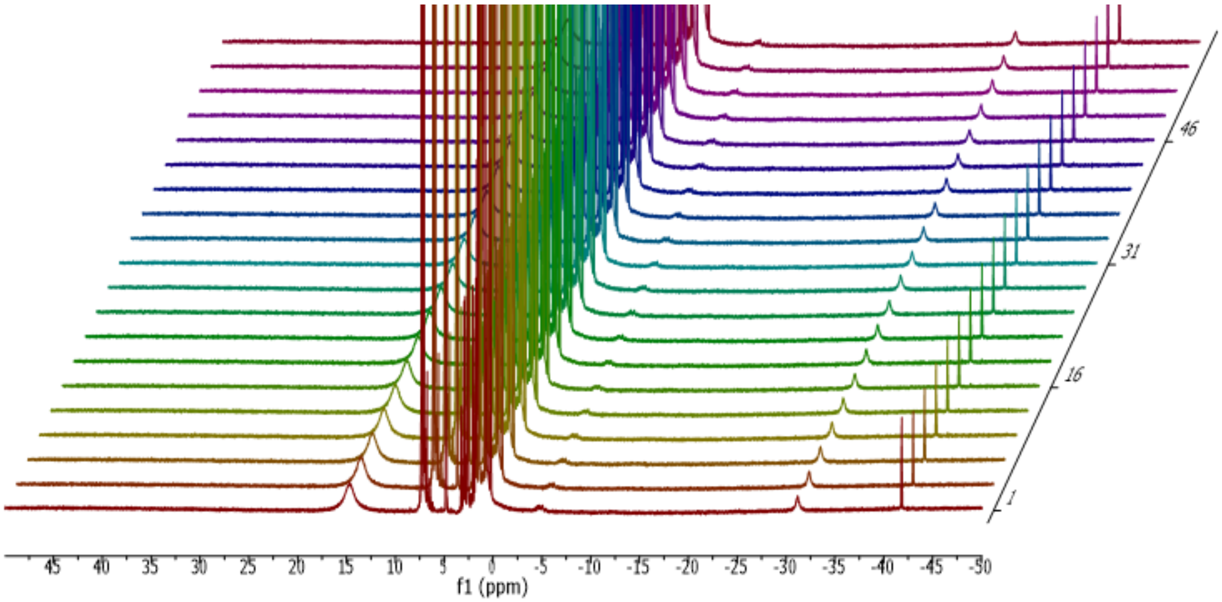

*Fig. S37. Monitoring of reaction of **2-H** with 1 eq. tri-tert-butylphenoxy radical over 30 minutes at 25°C. Signals of paramagnetic complex are seen at -31 ppm and +15 ppm. One of every three timepoints is shown for clarity.*

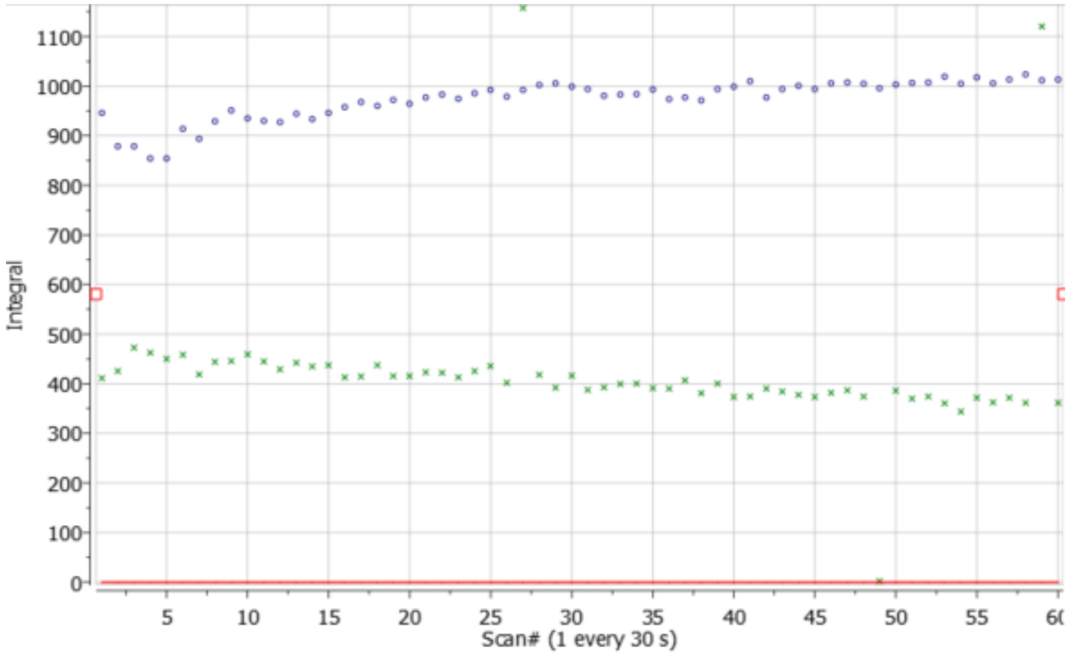

*Fig. S38. Time dependent integrals of **2-H** and **2•**. Blue series: integral for **2-H** methyl groups at 2.56 ppm. Green series: integral for **2•** methyl groups at -31 ppm.*

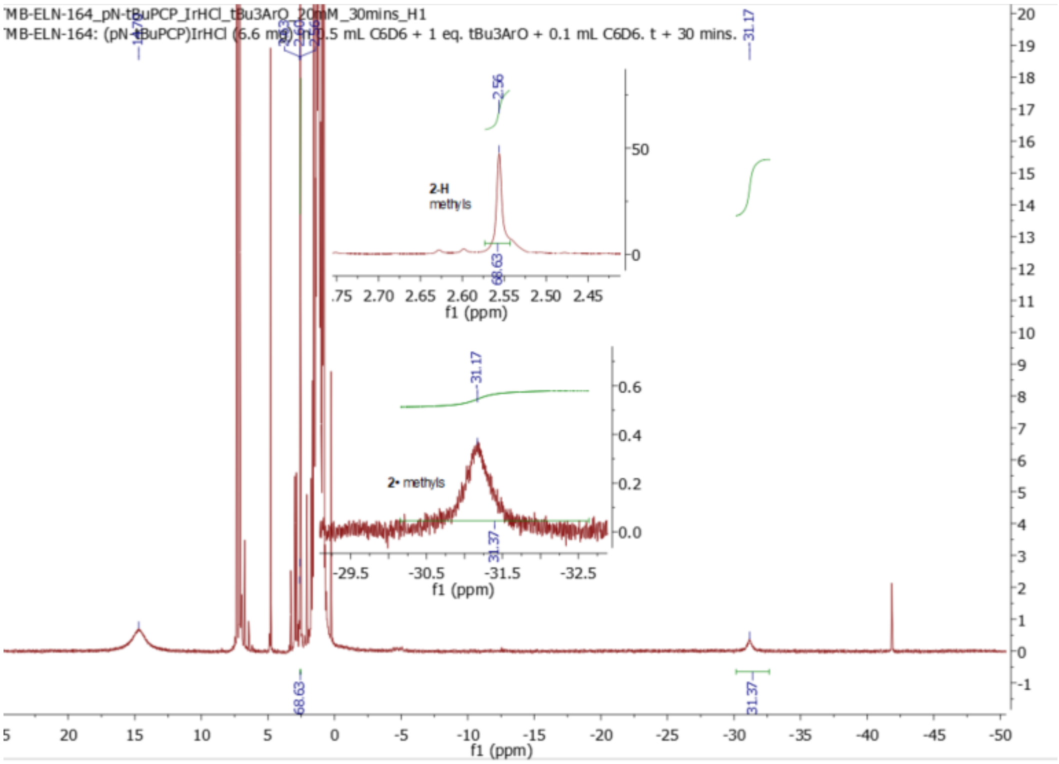

*Fig. S39. Final spectrum after 30 minutes, showing 31% conversion to **2•.***

Cyclic voltammetry

General considerations:

Cyclic voltammograms are recorded in tetrahydrofuran inside an argon-filled glovebox, using an Epsilon Eclipse™ Potentiostat (BASi Research Products, West Lafayette, IN). Solution concentrations are 1 mM in the respective analytes, with 100 mM tetrabutylammonium hexafluorophosphate as supporting electrolyte. Working electrode is glassy carbon (3 mm diameter, BASi), counter-electrode is a platinum wire, and reference electrode is Ag/AgNO3. Potentials are referenced to the ferrocene/ferrocenium redox couple using ferrocene as an internal standard, which was added to the analyte solutions after study.

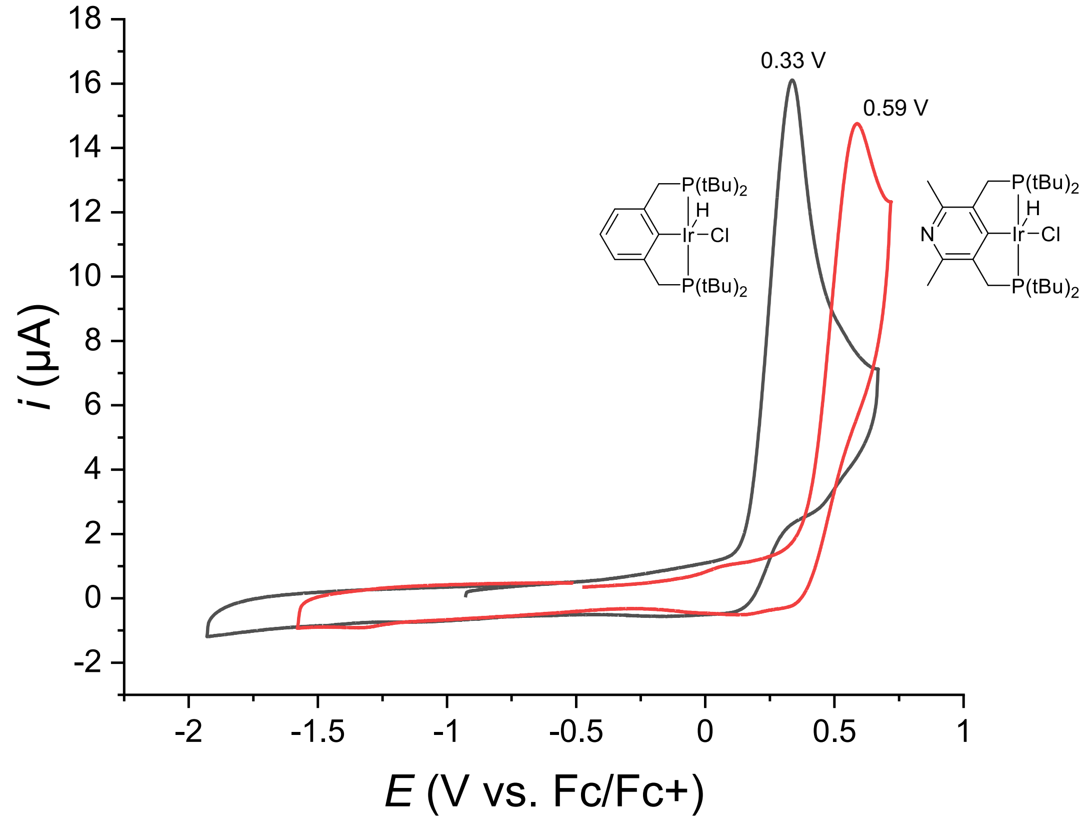

*Fig. S40. Cyclic voltammogram of **2-H** (Red) vs. **3-H** (Black) at a scan rate of (100 mV/s), measured in THF.*

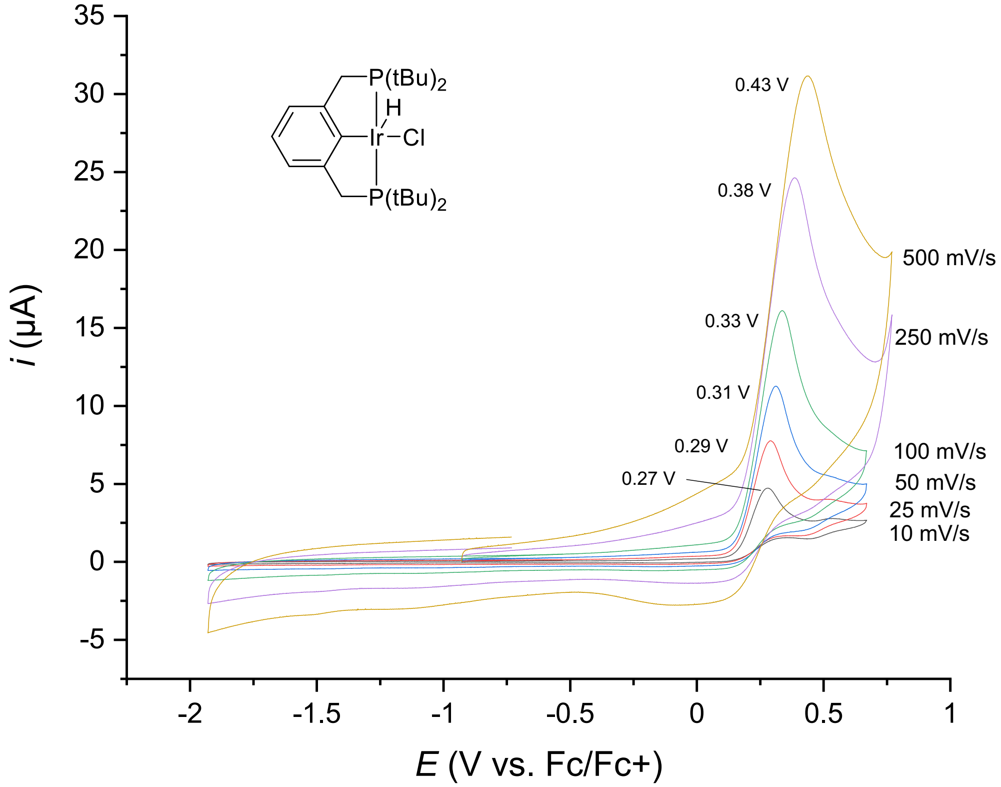

*Fig. S41. Cyclic voltammograms of complex **3-H** at variable scan rates in THF. Potentials are defined against Fc/Fc+.*

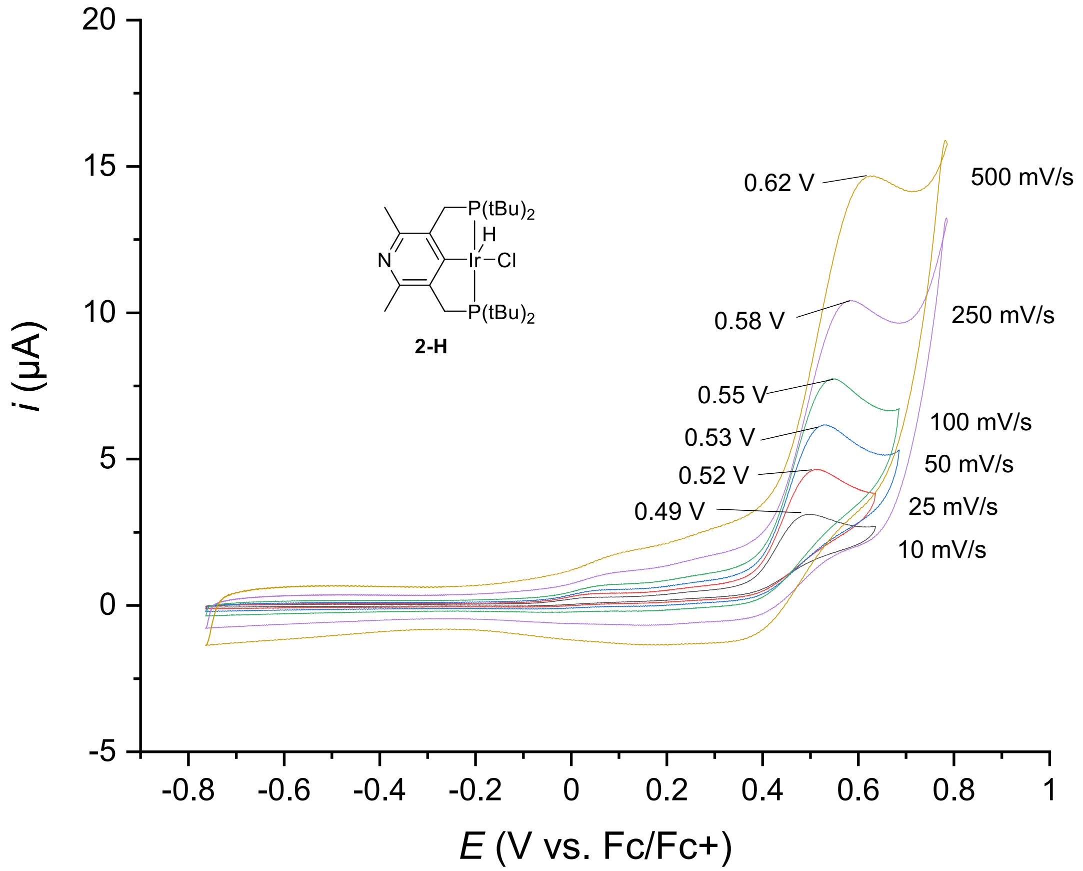

Fig. S42. Cyclic voltammograms of complex ***2-H*** *at variable scan rates in THF. Potentials are defined against Fc/Fc+.*

Computational Methods

Except for the molecules in Figure 7, geometry optimization was done with *Gaussian-09*[1] using the M06L density functional[2] in a polarizable continuum representing toluene as solvent.[3,4] For this purpose the 6-311G(d,p) basis set was used on the main group elements,[5,6] while iridium carried the SDD relativistic effective core potential (ECP) and associated basis set[7] augmented with a polarization *f*-function from the Frenking basis set.[8] Normal mode vibrational analyses using this level afforded the thermal and entropy correction terms to the standard state Gibbs free energy at 298 K and 1 M concentrations.[9,10] Final electronic energies were obtained in dichloromethane from single point calculations on the given optimized geometries using the M06L-D3 functional,[11,12] the def2-tzvp basis set on the non-metal elements, and the def2qzvp basis set with the associated ECP on Ir along with the density fitting approximation of Ahlrichs and coworkers as implemented in *Gaussian-16*.[13] The same M06L-D3 functional and def2 basis sets were also used to compute the final energies of the molecules in Figure 6, but in this case the initial optimization was done without a polarizable continuum, while the final electronic energies were computed without use of a density fitting approximation, and with a polarizable continuum representing *n*-heptane solvent to better model typical conditions of alkane dehydrogenation catalysis.

References

(1) Gaussian 09, Rev. A.03. Frisch, M. J. et. al. Gaussian, Inc., Wallingford CT, 2009.

(2) Zhao, Y.; Truhlar, D. G. A new local density functional for main-group thermochemistry, transition metal bonding, thermochemical kinetics, and noncovalent interactions. *J. Chem. Phys.* **2006**, 125, 194101-194118. https://doi.org/10.1063/1.2370993

(3) Marenich, A, V.; Cramer, C. J.; Truhlar, C. J. Universal solvation model based on solute electron density and on a continuum model of the solvent defined by the bulk dielectric constant and atomic surface tensions. *J. Phys. Chem. B* **2009**, *113*, 6378-6396. https://doi.org/10.1021/jp810292n

(4) Ribeiro, R. F.; Marenich, A. V.; Cramer, C. J.; Truhlar D. G. Use of Solution-Phase Vibrational Frequencies in Continuum Models for the Free Energy of Solvation. *J. Phys. Chem. B* **2011**, *115*, 14556–14562. https://doi.org/10.1021/jp205508z

(5) Raghavachari, K.; Binkley, J. S.; Seeger, R.; Pople, J. A. Self-Consistent Molecular Orbital Methods. 20. Basis set for correlated wave-functions. *J. Chem. Phys.* **1980**, *72*, 650-654. https://doi.org/10.1063/1.438955

(6) McLean, A. D.; Chandler, G. S. Contracted Gaussian basis sets for molecular calculations. I. Second row atoms, Z = 11–18. *J. Chem. Phys.* **1980**, 72, 5639-5648. https://doi.org/10.1063/1.438980

(7) Andrae, D.; Häußermann, U.; Dolg, M.; Stoll, H.; Preuss, H. Energy-adjusted *ab initio* pseudopotentials for the second and third row transition elements. *Theor. Chim. Acta.* **1990**, *77*, 123-141. https://doi.org/10.1007/BF01114537

(8) Ehlers, A. W.; Böhlers, A. W.; Bohme, M.; Dapprich, S.; Gobbi, A.; Höllwarth, A.; Jonas, V.; Köhler, K. F.; Stegmann, R.; Veldkamp, A.; Frenking, G. A set of f-polarization Functions for Pseudo-Potential Basis Sets of the Transition Metals Sc-Cu, Y-Ag and La-Au *Chem. Phys. Lett.* **1993**, *208*, 111–114. https://doi.org/10.1016/0009-2614(93)80086-5

(9) Cramer, C. J. Essentials of Computational Chemistry: Theories and Models, 2nd ed.; Wiley, 2004.

(10) Jensen, H. I. Predicting accurate absolute binding energies in aqueous solution: thermodynamic considerations for electronic structure methods. *Phys.Chem.Chem.Phys.* **2015**, *17*, 12441-12451. https://doi.org/10.1039/C5CP00628G.

(11) Grimme, S.; Antony, J.; Ehrlich, S.; Krieg, H. A consistent and accurate ab initio parametrization of density functional dispersion correction (DFT-D) for the 94 elements H-Pu. https://doi.org/10.1063/1.3382344

(12) Becke, A. D.; Johnson, E. R. A density-functional model of the dispersion interaction. *J. Chem. Phys.* **2005**, *123*, 154101-154109. https://doi.org/10.1063/1.2065267.

(13) Gaussian 16, Rev. D.01. Frisch, M. J. et. al. Gaussian, Inc., Wallingford CT, 2016.

(14) Hehre, W. J.; Ditchfield, R.; Pople, J. A. Self-Consistent Molecular Orbital Methods. 12. Further extensions of Gaussian-type basis sets for use in molecular-orbital studies of organic-molecules. *J. Chem. Phys.,* **1972**, *52*, 2257-2261. DOI: https://doi.org/10.1063/1.1677527

X-Ray Diffraction Data

General comments

X-Ray diffraction was obtained from an oil coated crystal mounted on a glass fiber. X-ray diffraction data were collected on a Bruker Smart APEX CCD diffractometer with graphite mono-chromatized MoKα radiation (λ = 0.71073Å) at a temperature of 100 K or 120 K. Crystals were immersed in Paratone oil and placed on a glass needle or nylon loop. The data were corrected for Lorenz effects, polarization, and absorption, the latter by a multiscan (SADABS) method.S1 The structures were solved by direct methods (SHELXS86).S2 All non-hydrogen atoms were refined (SHELXL97)3 based upon Fobs. All hydrogen atom coordinates were calculated with idealized geometries (SHELXL97). Scattering factors (fo, f', f") are as described in SHELXL97.

Proligand 1

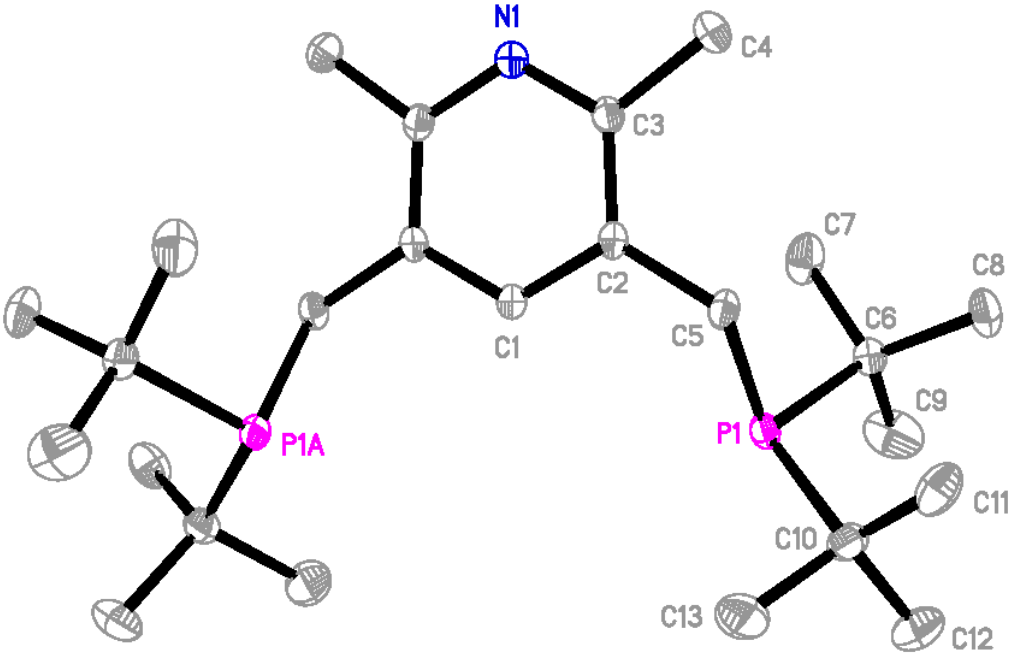

Table S2. Crystal data and structure refinement for Proligand 1

Identification code	tariq_ligand_a	
Empirical formula	C25 H47 N P2	
Formula weight	423.57	
Temperature	120(2) K	
Wavelength	0.71073 Å	
Crystal system	Monoclinic	
Space group	C2/c	
Unit cell dimensions	a = 24.3743(19) Å	[illegible]= 90°.
	b = 10.3424(8) Å	[illegible]= 95.7890(10)°.
	c = 10.5170(8) Å	[illegible] = 90°.
Volume	2637.7(4) Å^3	
Z	4	
Density (calculated)	1.067 Mg/m^3	
Absorption coefficient	0.175 mm^{-1}	
F(000)	936	
Crystal size	.34 x .44 x .54 mm^3	
Theta range for data collection	1.679 to 33.229°.	
Index ranges	-37<=h<=37, -15<=k<=15, -15<=l<=16	
Reflections collected	19546	
Independent reflections	5049 [R(int) = 0.0262]	
Completeness to theta = 25.242°	99.9 %	
Absorption correction	Semi-empirical from equivalents	
Max. and min. transmission	0.7465 and 0.7119	
Refinement method	Full-matrix least-squares on F^2	
Data / restraints / parameters	5049 / 0 / 135	
Goodness-of-fit on F^2	1.063	
Final R indices [I>2sigma(I)]	R1 = 0.0348, wR2 = 0.0939	
R indices (all data)	R1 = 0.0412, wR2 = 0.0986	
Extinction coefficient	n/a	
Largest diff. peak and hole	0.547 and -0.191 e.Å-3	

Table S3. Atomic coordinates (x 104) and equivalent isotropic displacement parameters ($Å^2$x 10^3)

U(eq) is defined as one third of the trace of the orthogonalized U^{ij} tensor.

	x	y	z	U(eq)
P(1)	3876(1)	7505(1)	3183(1)	16(1)
N(1)	5000	3422(1)	2500	17(1)
C(1)	5000	6078(1)	2500	16(1)
C(2)	4644(1)	5424(1)	3232(1)	15(1)
C(3)	4666(1)	4070(1)	3217(1)	16(1)
C(4)	4325(1)	3236(1)	4011(1)	22(1)
C(5)	4276(1)	6172(1)	4040(1)	17(1)
C(6)	3198(1)	6696(1)	2646(1)	17(1)
C(7)	3345(1)	5550(1)	1824(1)	29(1)
C(8)	2859(1)	6197(1)	3680(1)	24(1)
C(9)	2844(1)	7623(1)	1775(1)	39(1)
C(10)	3796(1)	8624(1)	4569(1)	21(1)
C(11)	3685(1)	7997(1)	5837(1)	26(1)
C(12)	3346(1)	9622(1)	4182(1)	32(1)
C(13)	4351(1)	9340(1)	4768(1)	37(1)

Table S4. Bond lengths [Å] and angles [°]

P(1)-C(5)	1.8673(8)	C(7)-H(7B)	0.9800
P(1)-C(10)	1.8860(8)	C(7)-H(7C)	0.9800
P(1)-C(6)	1.8866(8)	C(8)-H(8A)	0.9800
N(1)-C(3)	1.3432(9)	C(8)-H(8B)	0.9800
N(1)-C(3)#1	1.3433(9)	C(8)-H(8C)	0.9800
C(1)-C(2)	1.3920(9)	C(9)-H(9A)	0.9800
C(1)-C(2)#1	1.3920(9)	C(9)-H(9B)	0.9800
C(1)-H(1)	0.9500	C(9)-H(9C)	0.9800
C(2)-C(3)	1.4010(11)	C(10)-C(11)	1.5309(13)
C(2)-C(5)	1.5111(10)	C(10)-C(12)	1.5318(12)
C(3)-C(4)	1.5076(11)	C(10)-C(13)	1.5386(13)
C(4)-H(4A)	0.9800	C(11)-H(11A)	0.9800
C(4)-H(4B)	0.9800	C(11)-H(11B)	0.9800
C(4)-H(4C)	0.9800	C(11)-H(11C)	0.9800
C(5)-H(5A)	0.9900	C(12)-H(12A)	0.9800
C(5)-H(5AB)	0.9900	C(12)-H(12B)	0.9800
C(6)-C(8)	1.5210(11)	C(12)-H(12C)	0.9800
C(6)-C(7)	1.5307(12)	C(13)-H(13A)	0.9800
C(6)-C(9)	1.5311(12)	C(13)-H(13B)	0.9800
C(7)-H(7A)	0.9800	C(13)-H(13C)	0.9800

C(5)-P(1)-C(10)	99.72(4)	H(7B)-C(7)-H(7C)	109.5
C(5)-P(1)-C(6)	102.45(3)	C(6)-C(8)-H(8A)	109.5
C(10)-P(1)-C(6)	110.35(4)	C(6)-C(8)-H(8B)	109.5
C(3)-N(1)-C(3)#1	120.11(9)	H(8A)-C(8)-H(8B)	109.5
C(2)-C(1)-C(2)#1	121.78(10)	C(6)-C(8)-H(8C)	109.5
C(2)-C(1)-H(1)	119.1	H(8A)-C(8)-H(8C)	109.5
C(2)#1-C(1)-H(1)	119.1	H(8B)-C(8)-H(8C)	109.5
C(1)-C(2)-C(3)	116.96(7)	C(6)-C(9)-H(9A)	109.5
C(1)-C(2)-C(5)	120.04(7)	C(6)-C(9)-H(9B)	109.5
C(3)-C(2)-C(5)	122.95(7)	H(9A)-C(9)-H(9B)	109.5
N(1)-C(3)-C(2)	122.07(7)	C(6)-C(9)-H(9C)	109.5
N(1)-C(3)-C(4)	115.13(7)	H(9A)-C(9)-H(9C)	109.5
C(2)-C(3)-C(4)	122.79(7)	H(9B)-C(9)-H(9C)	109.5
C(3)-C(4)-H(4A)	109.5	C(11)-C(10)-C(12)	109.39(7)
C(3)-C(4)-H(4B)	109.5	C(11)-C(10)-C(13)	108.38(8)
H(4A)-C(4)-H(4B)	109.5	C(12)-C(10)-C(13)	107.96(8)
C(3)-C(4)-H(4C)	109.5	C(11)-C(10)-P(1)	117.05(6)
H(4A)-C(4)-H(4C)	109.5	C(12)-C(10)-P(1)	109.57(6)
H(4B)-C(4)-H(4C)	109.5	C(13)-C(10)-P(1)	104.04(6)
C(2)-C(5)-P(1)	114.68(5)	C(10)-C(11)-H(11A)	109.5
C(2)-C(5)-H(5A)	108.6	C(10)-C(11)-H(11B)	109.5
P(1)-C(5)-H(5A)	108.6	H(11A)-C(11)-H(11B)	109.5
C(2)-C(5)-H(5AB)	108.6	C(10)-C(11)-H(11C)	109.5
P(1)-C(5)-H(5AB)	108.6	H(11A)-C(11)-H(11C)	109.5
H(5A)-C(5)-H(5AB)	107.6	H(11B)-C(11)-H(11C)	109.5
C(8)-C(6)-C(7)	108.46(7)	C(10)-C(12)-H(12A)	109.5
C(8)-C(6)-C(9)	109.05(8)	C(10)-C(12)-H(12B)	109.5
C(7)-C(6)-C(9)	107.24(8)	H(12A)-C(12)-H(12B)	109.5
C(8)-C(6)-P(1)	117.34(5)	C(10)-C(12)-H(12C)	109.5
C(7)-C(6)-P(1)	105.35(6)	H(12A)-C(12)-H(12C)	109.5
C(9)-C(6)-P(1)	108.92(6)	H(12B)-C(12)-H(12C)	109.5
C(6)-C(7)-H(7A)	109.5	C(10)-C(13)-H(13A)	109.5
C(6)-C(7)-H(7B)	109.5	C(10)-C(13)-H(13B)	109.5
H(7A)-C(7)-H(7B)	109.5	H(13A)-C(13)-H(13B)	109.5
C(6)-C(7)-H(7C)	109.5	C(10)-C(13)-H(13C)	109.5
H(7A)-C(7)-H(7C)	109.5	H(13A)-C(13)-H(13C)	109.5

H(13B)-C(13)-H(13C)	109.5

Symmetry transformations used to generate equivalent atoms:

#1 -x+1,y,-z+1/2

Table S5. Anisotropic displacement parameters ($Å^2 x 10^3$).
The anisotropic displacement factor exponent takes the form: $-2\pi^2[h^2 a^{*2}U^{11} + ... + 2 h k a^* b^* U^{12}]$

	U^{11}	U^{22}	U^{33}	U^{23}	U^{13}	U^{12}
P(1)	14(1)	17(1)	17(1)	2(1)	4(1)	-1(1)
N(1)	16(1)	17(1)	19(1)	0	4(1)	0
C(1)	13(1)	16(1)	19(1)	0	4(1)	0
C(2)	12(1)	18(1)	17(1)	0(1)	4(1)	1(1)
C(3)	14(1)	18(1)	17(1)	1(1)	3(1)	-1(1)
C(4)	22(1)	21(1)	24(1)	3(1)	8(1)	-2(1)
C(5)	14(1)	20(1)	17(1)	0(1)	4(1)	2(1)
C(6)	16(1)	21(1)	15(1)	1(1)	1(1)	0(1)
C(7)	24(1)	39(1)	25(1)	-15(1)	4(1)	-4(1)
C(8)	18(1)	32(1)	23(1)	-3(1)	7(1)	-7(1)
C(9)	34(1)	36(1)	42(1)	13(1)	-18(1)	-1(1)
C(10)	18(1)	18(1)	28(1)	-4(1)	2(1)	1(1)
C(11)	27(1)	32(1)	19(1)	-7(1)	2(1)	7(1)
C(12)	34(1)	23(1)	40(1)	-1(1)	4(1)	11(1)
C(13)	28(1)	27(1)	55(1)	-13(1)	3(1)	-9(1)

Table S6. Hydrogen coordinates (x 10^4) and isotropic displacement parameters ($Å^2$x 10^3)

	x	y	z	U(eq)
H(1)	5000	6997	2500	19
H(4A)	4424	3420	4919	33
H(4B)	4396	2323	3841	33
H(4C)	3933	3424	3788	33
H(5A)	4506	6545	4779	20
H(5AB)	4013	5563	4379	20
H(7A)	3006	5155	1419	44
H(7B)	3553	4909	2363	44
H(7C)	3570	5853	1163	44
H(8A)	2533	5740	3282	36
H(8B)	2741	6927	4182	36
H(8C)	3083	5603	4242	36
H(9A)	2521	7162	1373	58
H(9B)	3062	7956	1111	58
H(9C)	2724	8346	2282	58
H(11A)	3662	8670	6485	39
H(11B)	3986	7402	6115	39
H(11C)	3337	7518	5722	39
H(12A)	3365	10321	4814	48
H(12B)	2983	9205	4143	48
H(12C)	3401	9978	3341	48
H(13A)	4337	9987	5444	55
H(13B)	4423	9770	3971	55
H(13C)	4647	8719	5014	55

Table S7. Torsion angles [°]

C(2)#1-C(1)-C(2)-C(3)	1.02(5)
C(2)#1-C(1)-C(2)-C(5)	178.34(7)
C(3)#1-N(1)-C(3)-C(2)	1.12(5)
C(3)#1-N(1)-C(3)-C(4)	-178.10(7)
C(1)-C(2)-C(3)-N(1)	-2.14(10)
C(5)-C(2)-C(3)-N(1)	-179.39(6)
C(1)-C(2)-C(3)-C(4)	177.01(6)
C(5)-C(2)-C(3)-C(4)	-0.23(11)
C(1)-C(2)-C(5)-P(1)	49.48(8)
C(3)-C(2)-C(5)-P(1)	-133.36(7)
C(10)-P(1)-C(5)-C(2)	-152.18(6)
C(6)-P(1)-C(5)-C(2)	94.29(6)
C(5)-P(1)-C(6)-C(8)	62.56(7)
C(10)-P(1)-C(6)-C(8)	-42.89(7)
C(5)-P(1)-C(6)-C(7)	-58.21(6)
C(10)-P(1)-C(6)-C(7)	-163.66(6)
C(5)-P(1)-C(6)-C(9)	-172.98(7)
C(10)-P(1)-C(6)-C(9)	81.57(8)
C(5)-P(1)-C(10)-C(11)	-39.17(7)
C(6)-P(1)-C(10)-C(11)	68.10(7)
C(5)-P(1)-C(10)-C(12)	-164.43(6)
C(6)-P(1)-C(10)-C(12)	-57.15(7)
C(5)-P(1)-C(10)-C(13)	80.34(7)
C(6)-P(1)-C(10)-C(13)	-172.38(6)

Symmetry transformations used to generate equivalent atoms:

#1 -x+1,y,-z+1/2

Complex 2-H

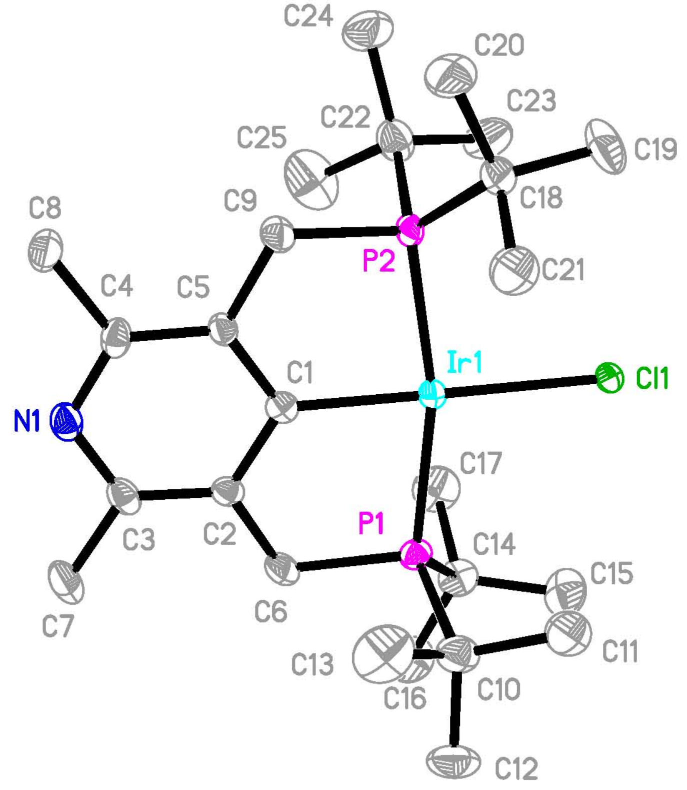

C24
C20
C23
C22
C25
C19
C9
C18
C8
P2
C21
C4
C5
C1
Ir1
Cl1
N1
C17
P1
C14
C3
C2
C15
C6
C7
C11
C13
C10
C16
C12

Table S8. Crystal data and structure refinement

Empirical formula	C25 H47 Cl Ir N P2	
Formula weight	651.22	
Temperature	120(2) K	
Wavelength	0.71073 Å	
Crystal system	Monoclinic	
Space group	$P2_1/n$	
Unit cell dimensions	a = 8.1501(11) Å	α= 90°.
	b = 15.531(2) Å	β= 93.822(2)°.
	c = 21.958(3) Å	γ = 90°.
Volume	2773.2(6) $Å^3$	
Z	4	
Density (calculated)	1.560 Mg/m^3	
Absorption coefficient	5.039 mm^{-1}	
F(000)	1312	
Crystal size	0.220 x 0.060 x 0.040 mm^3	
Theta range for data collection	1.607 to 29.127°.	
Index ranges	-11<=h<=10, -21<=k<=21, -30<=l<=30	
Reflections collected	31084	
Independent reflections	7428 [R(int) = 0.0654]	
Completeness to theta = 25.242°	100.0 %	
Absorption correction	Semi-empirical from equivalents	
Max. and min. transmission	0.7466 and 0.5098	
Refinement method	Full-matrix least-squares on F^2	
Data / restraints / parameters	7428 / 3 / 288	
Goodness-of-fit on F^2	1.117	
Final R indices [I>2sigma(I)]	R1 = 0.0411, wR2 = 0.0926	
R indices (all data)	R1 = 0.0487, wR2 = 0.0963	
Extinction coefficient	n/a	
Largest diff. peak and hole	2.322 and -1.999 e.$Å^{-3}$	

Table S9. Atomic coordinates (x 10^4) and equivalent isotropic displacement parameters ($Å^2$x 10^3)
U(eq) is defined as one third of the trace of the orthogonalized U^{ij} tensor.

	x	y	z	U(eq)
Ir(1)	6444(1)	7074(1)	3569(1)	17(1)
Cl(1)	8043(1)	8330(1)	3289(1)	22(1)
P(1)	7015(2)	6231(1)	2749(1)	19(1)
P(2)	5528(2)	7637(1)	4458(1)	19(1)
N(1)	3067(5)	4641(3)	4080(2)	23(1)
C(1)	5066(5)	6048(3)	3785(2)	18(1)
C(2)	4787(6)	5344(3)	3386(2)	20(1)
C(3)	3813(6)	4662(3)	3551(2)	22(1)
C(4)	3309(6)	5308(3)	4466(2)	22(1)
C(5)	4309(6)	6002(3)	4345(2)	20(1)
C(6)	5480(6)	5369(3)	2767(2)	24(1)
C(7)	3476(7)	3900(3)	3136(3)	32(1)
C(8)	2405(7)	5266(3)	5042(2)	29(1)
C(9)	4661(6)	6693(3)	4823(2)	24(1)
C(10)	9055(7)	5691(4)	2870(3)	32(1)
C(11)	10390(7)	6345(4)	3028(3)	43(2)
C(12)	9559(8)	5155(4)	2326(3)	44(2)
C(13)	8959(9)	5059(5)	3401(3)	52(2)
C(14)	6647(7)	6690(4)	1965(2)	28(1)
C(15)	8078(9)	7279(4)	1809(3)	42(2)
C(16)	6362(8)	6009(4)	1466(2)	41(2)
C(17)	5113(8)	7250(4)	1992(3)	44(2)
C(18)	7084(6)	8059(3)	5053(2)	25(1)
C(19)	7691(8)	8945(4)	4879(3)	41(2)
C(20)	6453(8)	8088(4)	5691(3)	41(2)
C(21)	8528(7)	7429(4)	5062(3)	38(1)
C(22)	3722(7)	8378(3)	4317(3)	31(1)
C(23)	4197(8)	9173(4)	3960(3)	43(2)
C(24)	2950(8)	8686(4)	4903(3)	46(2)
C(25)	2396(9)	7868(5)	3950(4)	58(2)

Table S10. Bond lengths [Å] and angles [°]

Ir(1)-C(1)	2.025(5)	C(11)-H(11A)	0.9800
Ir(1)-P(1)	2.2984(12)	C(11)-H(11B)	0.9800
Ir(1)-P(2)	2.3076(12)	C(11)-H(11C)	0.9800
Ir(1)-Cl(1)	2.4475(11)	C(12)-H(12A)	0.9800
Ir(1)-H(1)	1.6000(10)	C(12)-H(12B)	0.9800
P(1)-C(6)	1.835(5)	C(12)-H(12C)	0.9800
P(1)-C(10)	1.865(6)	C(13)-H(13A)	0.9800
P(1)-C(14)	1.869(5)	C(13)-H(13B)	0.9800
P(2)-C(9)	1.835(5)	C(13)-H(13C)	0.9800
P(2)-C(18)	1.877(5)	C(14)-C(17)	1.527(8)
P(2)-C(22)	1.879(6)	C(14)-C(16)	1.530(8)
N(1)-C(4)	1.345(6)	C(14)-C(15)	1.538(8)
N(1)-C(3)	1.348(7)	C(15)-H(15A)	0.9800
C(1)-C(2)	1.410(6)	C(15)-H(15B)	0.9800
C(1)-C(5)	1.413(7)	C(15)-H(15C)	0.9800
C(2)-C(3)	1.386(7)	C(16)-H(16A)	0.9800
C(2)-C(6)	1.507(7)	C(16)-H(16B)	0.9800
C(3)-C(7)	1.508(7)	C(16)-H(16C)	0.9800
C(4)-C(5)	1.387(7)	C(17)-H(17A)	0.9800
C(4)-C(8)	1.508(7)	C(17)-H(17B)	0.9800
C(5)-C(9)	1.514(6)	C(17)-H(17C)	0.9800
C(6)-H(6A)	0.9900	C(18)-C(19)	1.520(7)
C(6)-H(6AB)	0.9900	C(18)-C(20)	1.525(8)
C(7)-H(7A)	0.9800	C(18)-C(21)	1.530(8)
C(7)-H(7AB)	0.9800	C(19)-H(19A)	0.9800
C(7)-H(7AC)	0.9800	C(19)-H(19B)	0.9800
C(8)-H(8A)	0.9800	C(19)-H(19C)	0.9800
C(8)-H(8AB)	0.9800	C(20)-H(20A)	0.9800
C(8)-H(8AC)	0.9800	C(20)-H(20B)	0.9800
C(9)-H(9A)	0.9900	C(20)-H(20C)	0.9800
C(9)-H(9AB)	0.9900	C(21)-H(21A)	0.9800
C(10)-C(11)	1.512(8)	C(21)-H(21B)	0.9800
C(10)-C(13)	1.530(9)	C(21)-H(21C)	0.9800
C(10)-C(12)	1.533(8)	C(22)-C(23)	1.526(8)

C(22)-C(25)	1.527(9)	C(24)-H(24B)	0.9800
C(22)-C(24)	1.544(8)	C(24)-H(24C)	0.9800
C(23)-H(23A)	0.9800	C(25)-H(25A)	0.9800
C(23)-H(23B)	0.9800	C(25)-H(25B)	0.9800
C(23)-H(23C)	0.9800	C(25)-H(25C)	0.9800
C(24)-H(24A)	0.9800		

C(1)-Ir(1)-P(1)	83.11(13)	N(1)-C(3)-C(2)	122.9(4)
C(1)-Ir(1)-P(2)	83.31(13)	N(1)-C(3)-C(7)	115.3(4)
P(1)-Ir(1)-P(2)	166.40(4)	C(2)-C(3)-C(7)	121.8(5)
C(1)-Ir(1)-Cl(1)	178.37(13)	N(1)-C(4)-C(5)	122.8(4)
P(1)-Ir(1)-Cl(1)	96.79(4)	N(1)-C(4)-C(8)	115.8(4)
P(2)-Ir(1)-Cl(1)	96.81(4)	C(5)-C(4)-C(8)	121.4(5)
C(1)-Ir(1)-H(1)	86.4(2)	C(4)-C(5)-C(1)	120.1(4)
P(1)-Ir(1)-H(1)	89.36(5)	C(4)-C(5)-C(9)	120.3(4)
P(2)-Ir(1)-H(1)	89.03(5)	C(1)-C(5)-C(9)	119.6(4)
Cl(1)-Ir(1)-H(1)	95.3(2)	C(2)-C(6)-P(1)	109.6(3)
C(6)-P(1)-C(10)	105.7(3)	C(2)-C(6)-H(6A)	109.8
C(6)-P(1)-C(14)	103.4(2)	P(1)-C(6)-H(6A)	109.8
C(10)-P(1)-C(14)	112.9(3)	C(2)-C(6)-H(6AB)	109.8
C(6)-P(1)-Ir(1)	102.99(16)	P(1)-C(6)-H(6AB)	109.8
C(10)-P(1)-Ir(1)	111.71(18)	H(6A)-C(6)-H(6AB)	108.2
C(14)-P(1)-Ir(1)	118.41(18)	C(3)-C(7)-H(7A)	109.5
C(9)-P(2)-C(18)	103.7(2)	C(3)-C(7)-H(7AB)	109.5
C(9)-P(2)-C(22)	104.0(3)	H(7A)-C(7)-H(7AB)	109.5
C(18)-P(2)-C(22)	112.7(2)	C(3)-C(7)-H(7AC)	109.5
C(9)-P(2)-Ir(1)	102.88(16)	H(7A)-C(7)-H(7AC)	109.5
C(18)-P(2)-Ir(1)	118.67(17)	H(7AB)-C(7)-H(7AC)	109.5
C(22)-P(2)-Ir(1)	112.78(19)	C(4)-C(8)-H(8A)	109.5
C(4)-N(1)-C(3)	117.9(4)	C(4)-C(8)-H(8AB)	109.5
C(2)-C(1)-C(5)	116.1(4)	H(8A)-C(8)-H(8AB)	109.5
C(2)-C(1)-Ir(1)	122.2(3)	C(4)-C(8)-H(8AC)	109.5
C(5)-C(1)-Ir(1)	121.7(3)	H(8A)-C(8)-H(8AC)	109.5
C(3)-C(2)-C(1)	120.1(4)	H(8AB)-C(8)-H(8AC)	109.5
C(3)-C(2)-C(6)	120.7(4)	C(5)-C(9)-P(2)	108.9(3)
C(1)-C(2)-C(6)	119.1(4)	C(5)-C(9)-H(9A)	109.9

P(2)-C(9)-H(9A)	109.9	H(15A)-C(15)-H(15B)	109.5
C(5)-C(9)-H(9AB)	109.9	C(14)-C(15)-H(15C)	109.5
P(2)-C(9)-H(9AB)	109.9	H(15A)-C(15)-H(15C)	109.5
H(9A)-C(9)-H(9AB)	108.3	H(15B)-C(15)-H(15C)	109.5
C(11)-C(10)-C(13)	109.1(5)	C(14)-C(16)-H(16A)	109.5
C(11)-C(10)-C(12)	108.5(5)	C(14)-C(16)-H(16B)	109.5
C(13)-C(10)-C(12)	105.9(5)	H(16A)-C(16)-H(16B)	109.5
C(11)-C(10)-P(1)	110.6(4)	C(14)-C(16)-H(16C)	109.5
C(13)-C(10)-P(1)	107.8(4)	H(16A)-C(16)-H(16C)	109.5
C(12)-C(10)-P(1)	114.6(4)	H(16B)-C(16)-H(16C)	109.5
C(10)-C(11)-H(11A)	109.5	C(14)-C(17)-H(17A)	109.5
C(10)-C(11)-H(11B)	109.5	C(14)-C(17)-H(17B)	109.5
H(11A)-C(11)-H(11B)	109.5	H(17A)-C(17)-H(17B)	109.5
C(10)-C(11)-H(11C)	109.5	C(14)-C(17)-H(17C)	109.5
H(11A)-C(11)-H(11C)	109.5	H(17A)-C(17)-H(17C)	109.5
H(11B)-C(11)-H(11C)	109.5	H(17B)-C(17)-H(17C)	109.5
C(10)-C(12)-H(12A)	109.5	C(19)-C(18)-C(20)	109.8(5)
C(10)-C(12)-H(12B)	109.5	C(19)-C(18)-C(21)	108.6(5)
H(12A)-C(12)-H(12B)	109.5	C(20)-C(18)-C(21)	108.3(5)
C(10)-C(12)-H(12C)	109.5	C(19)-C(18)-P(2)	110.9(4)
H(12A)-C(12)-H(12C)	109.5	C(20)-C(18)-P(2)	113.4(4)
H(12B)-C(12)-H(12C)	109.5	C(21)-C(18)-P(2)	105.6(4)
C(10)-C(13)-H(13A)	109.5	C(18)-C(19)-H(19A)	109.5
C(10)-C(13)-H(13B)	109.5	C(18)-C(19)-H(19B)	109.5
H(13A)-C(13)-H(13B)	109.5	H(19A)-C(19)-H(19B)	109.5
C(10)-C(13)-H(13C)	109.5	C(18)-C(19)-H(19C)	109.5
H(13A)-C(13)-H(13C)	109.5	H(19A)-C(19)-H(19C)	109.5
H(13B)-C(13)-H(13C)	109.5	H(19B)-C(19)-H(19C)	109.5
C(17)-C(14)-C(16)	109.7(5)	C(18)-C(20)-H(20A)	109.5
C(17)-C(14)-C(15)	107.8(5)	C(18)-C(20)-H(20B)	109.5
C(16)-C(14)-C(15)	109.5(5)	H(20A)-C(20)-H(20B)	109.5
C(17)-C(14)-P(1)	105.2(4)	C(18)-C(20)-H(20C)	109.5
C(16)-C(14)-P(1)	113.7(4)	H(20A)-C(20)-H(20C)	109.5
C(15)-C(14)-P(1)	110.7(4)	H(20B)-C(20)-H(20C)	109.5
C(14)-C(15)-H(15A)	109.5	C(18)-C(21)-H(21A)	109.5
C(14)-C(15)-H(15B)	109.5	C(18)-C(21)-H(21B)	109.5

H(21A)-C(21)-H(21B)	109.5	H(23A)-C(23)-H(23C)	109.5
C(18)-C(21)-H(21C)	109.5	H(23B)-C(23)-H(23C)	109.5
H(21A)-C(21)-H(21C)	109.5	C(22)-C(24)-H(24A)	109.5
H(21B)-C(21)-H(21C)	109.5	C(22)-C(24)-H(24B)	109.5
C(23)-C(22)-C(25)	110.1(6)	H(24A)-C(24)-H(24B)	109.5
C(23)-C(22)-C(24)	108.0(5)	C(22)-C(24)-H(24C)	109.5
C(25)-C(22)-C(24)	106.7(6)	H(24A)-C(24)-H(24C)	109.5
C(23)-C(22)-P(2)	110.9(4)	H(24B)-C(24)-H(24C)	109.5
C(25)-C(22)-P(2)	106.7(4)	C(22)-C(25)-H(25A)	109.5
C(24)-C(22)-P(2)	114.3(4)	C(22)-C(25)-H(25B)	109.5
C(22)-C(23)-H(23A)	109.5	H(25A)-C(25)-H(25B)	109.5
C(22)-C(23)-H(23B)	109.5	C(22)-C(25)-H(25C)	109.5
H(23A)-C(23)-H(23B)	109.5	H(25A)-C(25)-H(25C)	109.5
C(22)-C(23)-H(23C)	109.5	H(25B)-C(25)-H(25C)	109.5

Symmetry transformations used to generate equivalent atoms:

Table S11. Anisotropic displacement parameters ($Å^2$x 10^3).

The anisotropic displacement factor exponent takes the form: $-2\pi^2$[h^2 $a^{*2}U^{11}$ + ... + 2 h k a* b* U^{12}]

	U^{11}	U^{22}	U^{33}	U^{23}	U^{13}	U^{12}
Ir(1)	18(1)	15(1)	17(1)	0(1)	3(1)	-1(1)
Cl(1)	24(1)	12(1)	31(1)	0(1)	11(1)	-2(1)
P(1)	20(1)	21(1)	17(1)	-3(1)	2(1)	0(1)
P(2)	26(1)	14(1)	18(1)	1(1)	4(1)	-4(1)
N(1)	18(2)	18(2)	34(2)	3(2)	1(2)	0(2)
C(1)	16(2)	16(2)	23(2)	3(2)	0(2)	2(2)
C(2)	15(2)	20(2)	25(2)	0(2)	-1(2)	2(2)
C(3)	19(2)	16(2)	31(3)	-1(2)	-5(2)	2(2)
C(4)	17(2)	20(2)	29(2)	7(2)	3(2)	0(2)
C(5)	20(2)	17(2)	24(2)	0(2)	0(2)	1(2)
C(6)	28(3)	19(2)	24(2)	-6(2)	1(2)	-4(2)
C(7)	28(3)	19(2)	48(3)	-2(2)	-2(2)	-3(2)
C(8)	28(3)	23(3)	36(3)	5(2)	9(2)	-3(2)
C(9)	26(3)	24(2)	20(2)	-3(2)	0(2)	-3(2)
C(10)	25(3)	34(3)	37(3)	-9(2)	-1(2)	3(2)
C(11)	23(3)	41(3)	65(4)	-10(3)	-4(3)	2(2)
C(12)	35(3)	40(3)	55(4)	-18(3)	-1(3)	13(3)
C(13)	39(4)	64(5)	52(4)	15(3)	-6(3)	13(3)
C(14)	30(3)	33(3)	21(2)	2(2)	0(2)	1(2)
C(15)	52(4)	45(4)	31(3)	6(3)	12(3)	-8(3)
C(16)	43(4)	60(4)	20(3)	-6(3)	-4(2)	-6(3)
C(17)	37(3)	48(4)	46(4)	6(3)	-4(3)	9(3)
C(18)	25(3)	25(3)	26(2)	-5(2)	1(2)	-4(2)
C(19)	52(4)	31(3)	39(3)	-7(2)	-6(3)	-18(3)
C(20)	41(3)	55(4)	27(3)	-16(3)	4(2)	-9(3)
C(21)	23(3)	39(3)	52(4)	-7(3)	-3(2)	1(2)
C(22)	28(3)	23(3)	43(3)	1(2)	1(2)	3(2)
C(23)	49(4)	32(3)	50(4)	13(3)	21(3)	17(3)
C(24)	42(4)	41(4)	59(4)	0(3)	22(3)	12(3)
C(25)	45(4)	49(4)	78(5)	-1(4)	-26(4)	6(3)

Table S12. Hydrogen coordinates (x 10^4) and isotropic displacement parameters ($Å^2$ x 10^3)

	x	y	z	U(eq)
H(1)	7924(5)	6621(3)	3963(1)	25
H(6A)	4584	5472	2449	29
H(6AB)	5997	4809	2682	29
H(7A)	3010	4100	2738	48
H(7AB)	2695	3512	3318	48
H(7AC)	4506	3591	3083	48
H(8A)	1651	5756	5055	43
H(8AB)	3198	5286	5398	43
H(8AC)	1778	4728	5049	43
H(9A)	5450	6473	5149	28
H(9AB)	3633	6854	5010	28
H(11A)	11428	6046	3135	65
H(11B)	10086	6692	3376	65
H(11C)	10521	6721	2676	65
H(12A)	8705	4727	2219	66
H(12B)	10600	4861	2437	66
H(12C)	9694	5534	1976	66
H(13A)	8094	4634	3300	78
H(13B)	8704	5374	3769	78
H(13C)	10017	4764	3472	78
H(15A)	7760	7611	1440	63
H(15B)	9045	6927	1738	63
H(15C)	8342	7674	2149	63
H(16A)	6168	6292	1069	62
H(16B)	5402	5659	1551	62
H(16C)	7333	5638	1460	62
H(17A)	4851	7515	1592	66
H(17B)	5317	7702	2299	66
H(17C)	4187	6893	2102	66
H(19A)	8060	8927	4464	62
H(19B)	8609	9116	5164	62

H(19C)	6794	9363	4897	62
H(20A)	5543	8500	5697	61
H(20B)	7344	8268	5985	61
H(20C)	6065	7515	5801	61
H(21A)	8138	6847	5146	57
H(21B)	9362	7599	5381	57
H(21C)	9009	7437	4665	57
H(23A)	4700	8993	3587	64
H(23B)	4984	9520	4212	64
H(23C)	3212	9516	3851	64
H(24A)	1958	9023	4792	70
H(24B)	3742	9046	5143	70
H(24C)	2661	8186	5145	70
H(25A)	1402	8220	3887	88
H(25B)	2140	7344	4174	88
H(25C)	2794	7710	3554	88

Table S13. Torsion angles [°]

C(5)-C(1)-C(2)-C(3)	0.3(6)
Ir(1)-C(1)-C(2)-C(3)	-178.9(3)
C(5)-C(1)-C(2)-C(6)	176.8(4)
Ir(1)-C(1)-C(2)-C(6)	-2.4(6)
C(4)-N(1)-C(3)-C(2)	-1.0(7)
C(4)-N(1)-C(3)-C(7)	-179.3(4)
C(1)-C(2)-C(3)-N(1)	1.4(7)
C(6)-C(2)-C(3)-N(1)	-175.0(4)
C(1)-C(2)-C(3)-C(7)	179.7(4)
C(6)-C(2)-C(3)-C(7)	3.2(7)
C(3)-N(1)-C(4)-C(5)	-1.2(7)
C(3)-N(1)-C(4)-C(8)	178.1(4)
N(1)-C(4)-C(5)-C(1)	2.9(7)
C(8)-C(4)-C(5)-C(1)	-176.4(4)
N(1)-C(4)-C(5)-C(9)	-174.2(4)
C(8)-C(4)-C(5)-C(9)	6.6(7)
C(2)-C(1)-C(5)-C(4)	-2.3(7)
Ir(1)-C(1)-C(5)-C(4)	176.9(3)
C(2)-C(1)-C(5)-C(9)	174.8(4)
Ir(1)-C(1)-C(5)-C(9)	-6.0(6)
C(3)-C(2)-C(6)-P(1)	-169.1(4)
C(1)-C(2)-C(6)-P(1)	14.4(6)
C(10)-P(1)-C(6)-C(2)	99.8(4)
C(14)-P(1)-C(6)-C(2)	-141.4(4)
Ir(1)-P(1)-C(6)-C(2)	-17.6(4)
C(4)-C(5)-C(9)-P(2)	-165.0(4)
C(1)-C(5)-C(9)-P(2)	17.9(6)
C(18)-P(2)-C(9)-C(5)	-143.7(3)
C(22)-P(2)-C(9)-C(5)	98.3(4)
Ir(1)-P(2)-C(9)-C(5)	-19.5(4)
C(6)-P(1)-C(10)-C(11)	-165.2(4)
C(14)-P(1)-C(10)-C(11)	82.5(5)
Ir(1)-P(1)-C(10)-C(11)	-53.9(5)
C(6)-P(1)-C(10)-C(13)	-45.9(5)

C(14)-P(1)-C(10)-C(13)	-158.2(4)
Ir(1)-P(1)-C(10)-C(13)	65.4(5)
C(6)-P(1)-C(10)-C(12)	71.8(5)
C(14)-P(1)-C(10)-C(12)	-40.5(5)
Ir(1)-P(1)-C(10)-C(12)	-176.9(4)
C(6)-P(1)-C(14)-C(17)	77.3(4)
C(10)-P(1)-C(14)-C(17)	-169.0(4)
Ir(1)-P(1)-C(14)-C(17)	-35.7(5)
C(6)-P(1)-C(14)-C(16)	-42.8(5)
C(10)-P(1)-C(14)-C(16)	71.0(5)
Ir(1)-P(1)-C(14)-C(16)	-155.8(4)
C(6)-P(1)-C(14)-C(15)	-166.5(4)
C(10)-P(1)-C(14)-C(15)	-52.8(5)
Ir(1)-P(1)-C(14)-C(15)	80.5(4)
C(9)-P(2)-C(18)-C(19)	-168.7(4)
C(22)-P(2)-C(18)-C(19)	-57.0(5)
Ir(1)-P(2)-C(18)-C(19)	78.0(4)
C(9)-P(2)-C(18)-C(20)	-44.6(5)
C(22)-P(2)-C(18)-C(20)	67.1(5)
Ir(1)-P(2)-C(18)-C(20)	-157.9(4)
C(9)-P(2)-C(18)-C(21)	73.8(4)
C(22)-P(2)-C(18)-C(21)	-174.5(4)
Ir(1)-P(2)-C(18)-C(21)	-39.5(4)
C(9)-P(2)-C(22)-C(23)	-174.9(4)
C(18)-P(2)-C(22)-C(23)	73.5(5)
Ir(1)-P(2)-C(22)-C(23)	-64.2(5)
C(9)-P(2)-C(22)-C(25)	-55.0(5)
C(18)-P(2)-C(22)-C(25)	-166.6(5)
Ir(1)-P(2)-C(22)-C(25)	55.8(5)
C(9)-P(2)-C(22)-C(24)	62.7(5)
C(18)-P(2)-C(22)-C(24)	-48.9(5)
Ir(1)-P(2)-C(22)-C(24)	173.4(4)

Symmetry transformations used to generate equivalent atoms:

Table S14. Hydrogen bonds [Å and °].

D-H...A	d(D-H)	d(H...A)	d(D...A)	<(DHA)

Complex 4

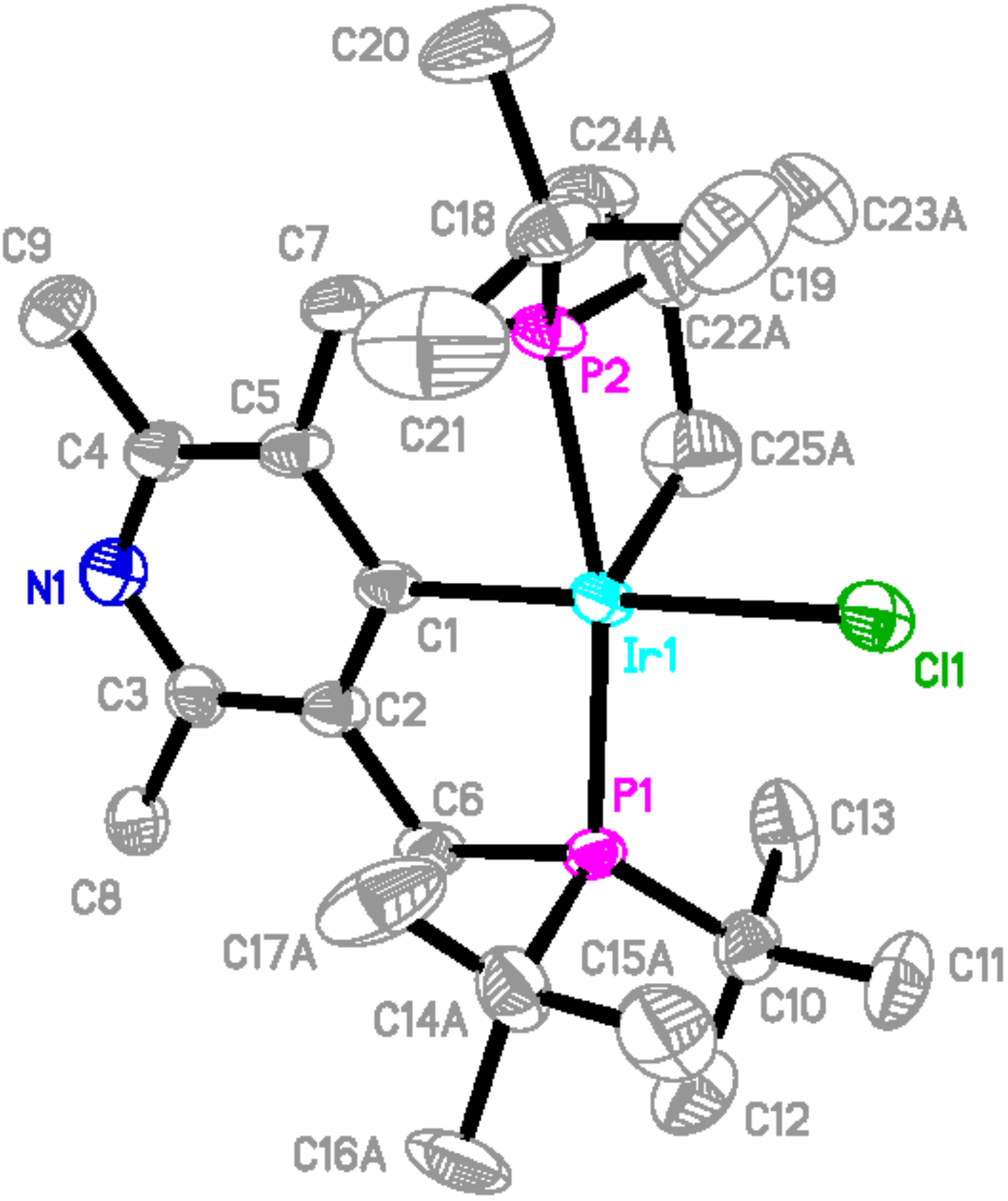

C20
C24A
C18
C23A
C9
C7
C19
C22A
P2
C5
C21
C4
C25A
N1
C1
Ir1
Cl1
C3
C2
C6
P1
C13
C8
C17A
C15A
C11
C14A
C10
C12
C16A

Table S15. Crystal data and structure refinement.

Identification code	tariq_3_again_0m	
Empirical formula	C25 H45 Cl Ir N P2	
Formula weight	649.21	
Temperature	120(2) K	
Wavelength	0.71073 Å	
Crystal system	Monoclinic	
Space group	$P2_1/n$	
Unit cell dimensions	a = 8.0217(19) Å	α= 90°.
	b = 15.278(4) Å	β= 91.466(4)°.
	c = 22.057(5) Å	γ = 90°.
Volume	2702.2(11) $Å^3$	
Z	4	
Density (calculated)	1.596 Mg/m^3	
Absorption coefficient	5.171 mm^{-1}	
F(000)	1304	
Crystal size	0.30 x 0.08 x 0.05 mm^3	
Theta range for data collection	2.278 to 29.130°.	
Index ranges	-10<=h<=10, -20<=k<=20, -29<=l<=28	
Reflections collected	17572	
Independent reflections	7166 [R(int) = 0.0489]	
Completeness to theta = 25.242°	99.7 %	
Absorption correction	Numerical	
Max. and min. transmission	0.7461 and 0.5245	
Refinement method	Full-matrix least-squares on F^2	
Data / restraints / parameters	7166 / 726 / 338	
Goodness-of-fit on F^2	1.038	
Final R indices [I>2sigma(I)]	R1 = 0.0420, wR2 = 0.0830	
R indices (all data)	R1 = 0.0655, wR2 = 0.0900	
Extinction coefficient	n/a	
Largest diff. peak and hole	1.662 and -1.607 e.$Å^{-3}$	

Table S16. Atomic coordinates (x 10^4) and equivalent isotropic displacement parameters ($Å^2$x 10^3)

U(eq) is defined as one third of the trace of the orthogonalized U^{ij} tensor.

	x	y	z	U(eq)
Ir(1)	3653(1)	7027(1)	6518(1)	24(1)
Cl(1)	2013(2)	8278(1)	6856(1)	42(1)
P(1)	4497(2)	7642(1)	5632(1)	27(1)
P(2)	3217(2)	6152(1)	7340(1)	27(1)
N(1)	6941(6)	4570(3)	5846(2)	34(1)
C(1)	5034(7)	5997(3)	6240(2)	26(1)
C(2)	5768(7)	5989(3)	5671(2)	31(1)
C(3)	6742(7)	5272(3)	5497(3)	32(1)
C(4)	6211(7)	4551(3)	6383(3)	32(1)
C(5)	5272(7)	5245(3)	6606(2)	31(1)
C(6)	5456(8)	6733(3)	5228(2)	35(1)
C(7)	4569(8)	5201(3)	7223(3)	39(1)
C(8)	7622(8)	5265(4)	4903(3)	36(1)
C(9)	6501(8)	3735(3)	6755(3)	41(2)
C(10)	3140(8)	8183(4)	5055(3)	39(1)
C(11)	2396(9)	9032(4)	5299(3)	52(2)
C(12)	4039(10)	8370(5)	4473(3)	55(2)
C(13)	1707(8)	7536(5)	4932(4)	58(2)
C(18)	3628(9)	6496(4)	8131(2)	38(1)
C(19)	2558(13)	7237(5)	8315(3)	80(3)
C(20)	3446(12)	5745(4)	8578(3)	70(3)
C(21)	5440(12)	6820(6)	8149(4)	87(3)
C(14A)	6440(20)	8342(14)	5885(9)	45(2)
C(15A)	5790(30)	9237(18)	6156(14)	69(3)
C(16A)	7498(18)	8644(9)	5333(6)	62(4)
C(17A)	7400(20)	7860(9)	6320(7)	72(5)
C(22A)	1150(20)	5727(12)	7013(8)	56(3)
C(23A)	-240(20)	6090(14)	7290(10)	80(4)
C(24A)	860(20)	4776(9)	6815(7)	68(4)

C(25A)	1500(20)	6307(10)	6388(7)	62(4)
C(14B)	930(20)	5793(11)	7241(7)	56(3)
C(15B)	-430(20)	6480(12)	7451(9)	80(4)
C(16B)	510(20)	5855(9)	6513(6)	60(4)
C(17B)	546(19)	4937(9)	7414(8)	67(4)
C(22B)	6150(20)	8261(14)	6000(9)	45(2)
C(23B)	5890(30)	9158(17)	6103(15)	69(3)
C(24B)	7899(16)	8046(9)	5801(8)	58(4)
C(25B)	5813(15)	7731(8)	6648(5)	38(3)

Table S17. Bond lengths [Å] and angles [°]

Ir(1)-C(1)	2.028(5)	C(10)-C(12)	1.517(9)
Ir(1)-C(25B)	2.053(11)	C(10)-C(11)	1.531(8)
Ir(1)-C(25A)	2.061(15)	C(10)-C(13)	1.535(9)
Ir(1)-P(2)	2.2857(14)	C(11)-H(11A)	0.9800
Ir(1)-P(1)	2.2864(14)	C(11)-H(11B)	0.9800
Ir(1)-Cl(1)	2.4470(14)	C(11)-H(11C)	0.9800
P(1)-C(22B)	1.80(2)	C(12)-H(12A)	0.9800
P(1)-C(6)	1.830(5)	C(12)-H(12B)	0.9800
P(1)-C(10)	1.849(7)	C(12)-H(12C)	0.9800
P(1)-C(14A)	1.96(2)	C(13)-H(13A)	0.9800
P(2)-C(7)	1.836(6)	C(13)-H(13B)	0.9800
P(2)-C(18)	1.844(6)	C(13)-H(13C)	0.9800
P(2)-C(22A)	1.90(2)	C(18)-C(19)	1.483(9)
P(2)-C(14B)	1.92(2)	C(18)-C(20)	1.522(8)
N(1)-C(3)	1.326(7)	C(18)-C(21)	1.535(11)
N(1)-C(4)	1.335(7)	C(19)-H(19A)	0.9800
C(1)-C(2)	1.399(7)	C(19)-H(19B)	0.9800
C(1)-C(5)	1.416(7)	C(19)-H(19C)	0.9800
C(2)-C(3)	1.405(7)	C(20)-H(20A)	0.9800
C(2)-C(6)	1.516(7)	C(20)-H(20B)	0.9800
C(3)-C(8)	1.504(7)	C(20)-H(20C)	0.9800
C(4)-C(5)	1.398(7)	C(21)-H(21A)	0.9800
C(4)-C(9)	1.508(7)	C(21)-H(21B)	0.9800
C(5)-C(7)	1.486(7)	C(21)-H(21C)	0.9800
C(6)-H(6A)	0.9900	C(14A)-C(17A)	1.42(2)
C(6)-H(6B)	0.9900	C(14A)-C(15A)	1.59(2)
C(7)-H(7A)	0.9900	C(14A)-C(16A)	1.571(17)
C(7)-H(7B)	0.9900	C(15A)-H(15A)	0.9800
C(8)-H(8A)	0.9800	C(15A)-H(15B)	0.9800
C(8)-H(8B)	0.9800	C(15A)-H(15C)	0.9800
C(8)-H(8C)	0.9800	C(16A)-H(16A)	0.9800
C(9)-H(9A)	0.9800	C(16A)-H(16B)	0.9800
C(9)-H(9B)	0.9800	C(16A)-H(16C)	0.9800
C(9)-H(9C)	0.9800	C(17A)-H(17A)	0.9800

C(17A)-H(17B)	0.9800	C(15B)-H(15F)	0.9800
C(17A)-H(17C)	0.9800	C(16B)-H(16D)	0.9800
C(22A)-C(23A)	1.40(2)	C(16B)-H(16E)	0.9800
C(22A)-C(24A)	1.53(2)	C(16B)-H(16F)	0.9800
C(22A)-C(25A)	1.669(18)	C(17B)-H(17D)	0.9800
C(23A)-H(23A)	0.9800	C(17B)-H(17E)	0.9800
C(23A)-H(23B)	0.9800	C(17B)-H(17F)	0.9800
C(23A)-H(23C)	0.9800	C(22B)-C(23B)	1.41(2)
C(24A)-H(24A)	0.9800	C(22B)-C(24B)	1.52(2)
C(24A)-H(24B)	0.9800	C(22B)-C(25B)	1.670(18)
C(24A)-H(24C)	0.9800	C(23B)-H(23D)	0.9800
C(25A)-H(25A)	0.9900	C(23B)-H(23E)	0.9800
C(25A)-H(25B)	0.9900	C(23B)-H(23F)	0.9800
C(14B)-C(17B)	1.399(19)	C(24B)-H(24D)	0.9800
C(14B)-C(15B)	1.59(2)	C(24B)-H(24E)	0.9800
C(14B)-C(16B)	1.635(18)	C(24B)-H(24F)	0.9800
C(15B)-H(15D)	0.9800	C(25B)-H(25C)	0.9900
C(15B)-H(15E)	0.9800	C(25B)-H(25D)	0.9900

C(1)-Ir(1)-C(25B)	89.0(4)	C(6)-P(1)-C(14A)	102.2(6)
C(1)-Ir(1)-C(25A)	90.3(5)	C(10)-P(1)-C(14A)	113.7(6)
C(1)-Ir(1)-P(2)	83.24(14)	C(22B)-P(1)-Ir(1)	93.5(5)
C(25B)-Ir(1)-P(2)	110.0(3)	C(6)-P(1)-Ir(1)	103.99(17)
C(25A)-Ir(1)-P(2)	69.7(4)	C(10)-P(1)-Ir(1)	126.2(2)
C(1)-Ir(1)-P(1)	83.34(14)	C(14A)-P(1)-Ir(1)	103.4(5)
C(25B)-Ir(1)-P(1)	68.7(3)	C(7)-P(2)-C(18)	105.4(3)
C(25A)-Ir(1)-P(1)	111.5(4)	C(7)-P(2)-C(22A)	100.9(6)
P(2)-Ir(1)-P(1)	166.54(5)	C(18)-P(2)-C(22A)	126.0(5)
C(1)-Ir(1)-Cl(1)	179.43(16)	C(7)-P(2)-C(14B)	108.9(6)
C(25B)-Ir(1)-Cl(1)	90.4(4)	C(18)-P(2)-C(14B)	109.6(5)
C(25A)-Ir(1)-Cl(1)	90.2(5)	C(7)-P(2)-Ir(1)	104.40(17)
P(2)-Ir(1)-Cl(1)	96.94(5)	C(18)-P(2)-Ir(1)	123.79(19)
P(1)-Ir(1)-Cl(1)	96.47(5)	C(22A)-P(2)-Ir(1)	92.8(5)
C(22B)-P(1)-C(6)	107.7(7)	C(14B)-P(2)-Ir(1)	104.0(5)
C(22B)-P(1)-C(10)	119.3(6)	C(3)-N(1)-C(4)	118.8(5)
C(6)-P(1)-C(10)	104.5(3)	C(2)-C(1)-C(5)	116.8(5)

C(2)-C(1)-Ir(1)	121.6(4)	H(9A)-C(9)-H(9C)	109.5
C(5)-C(1)-Ir(1)	121.6(4)	H(9B)-C(9)-H(9C)	109.5
C(1)-C(2)-C(3)	120.1(5)	C(12)-C(10)-C(11)	109.7(5)
C(1)-C(2)-C(6)	120.3(5)	C(12)-C(10)-C(13)	110.0(6)
C(3)-C(2)-C(6)	119.5(5)	C(11)-C(10)-C(13)	108.1(5)
N(1)-C(3)-C(2)	122.2(5)	C(12)-C(10)-P(1)	112.5(5)
N(1)-C(3)-C(8)	116.5(5)	C(11)-C(10)-P(1)	111.3(4)
C(2)-C(3)-C(8)	121.3(5)	C(13)-C(10)-P(1)	105.0(4)
N(1)-C(4)-C(5)	123.3(5)	C(10)-C(11)-H(11A)	109.5
N(1)-C(4)-C(9)	115.9(5)	C(10)-C(11)-H(11B)	109.5
C(5)-C(4)-C(9)	120.8(5)	H(11A)-C(11)-H(11B)	109.5
C(4)-C(5)-C(1)	118.7(5)	C(10)-C(11)-H(11C)	109.5
C(4)-C(5)-C(7)	120.6(5)	H(11A)-C(11)-H(11C)	109.5
C(1)-C(5)-C(7)	120.7(5)	H(11B)-C(11)-H(11C)	109.5
C(2)-C(6)-P(1)	108.6(4)	C(10)-C(12)-H(12A)	109.5
C(2)-C(6)-H(6A)	110.0	C(10)-C(12)-H(12B)	109.5
P(1)-C(6)-H(6A)	110.0	H(12A)-C(12)-H(12B)	109.5
C(2)-C(6)-H(6B)	110.0	C(10)-C(12)-H(12C)	109.5
P(1)-C(6)-H(6B)	110.0	H(12A)-C(12)-H(12C)	109.5
H(6A)-C(6)-H(6B)	108.3	H(12B)-C(12)-H(12C)	109.5
C(5)-C(7)-P(2)	109.4(4)	C(10)-C(13)-H(13A)	109.5
C(5)-C(7)-H(7A)	109.8	C(10)-C(13)-H(13B)	109.5
P(2)-C(7)-H(7A)	109.8	H(13A)-C(13)-H(13B)	109.5
C(5)-C(7)-H(7B)	109.8	C(10)-C(13)-H(13C)	109.5
P(2)-C(7)-H(7B)	109.8	H(13A)-C(13)-H(13C)	109.5
H(7A)-C(7)-H(7B)	108.2	H(13B)-C(13)-H(13C)	109.5
C(3)-C(8)-H(8A)	109.5	C(19)-C(18)-C(20)	109.4(6)
C(3)-C(8)-H(8B)	109.5	C(19)-C(18)-C(21)	107.6(6)
H(8A)-C(8)-H(8B)	109.5	C(20)-C(18)-C(21)	109.4(6)
C(3)-C(8)-H(8C)	109.5	C(19)-C(18)-P(2)	112.7(5)
H(8A)-C(8)-H(8C)	109.5	C(20)-C(18)-P(2)	112.3(4)
H(8B)-C(8)-H(8C)	109.5	C(21)-C(18)-P(2)	105.2(5)
C(4)-C(9)-H(9A)	109.5	C(18)-C(19)-H(19A)	109.5
C(4)-C(9)-H(9B)	109.5	C(18)-C(19)-H(19B)	109.5
H(9A)-C(9)-H(9B)	109.5	H(19A)-C(19)-H(19B)	109.5
C(4)-C(9)-H(9C)	109.5	C(18)-C(19)-H(19C)	109.5

H(19A)-C(19)-H(19C)	109.5
H(19B)-C(19)-H(19C)	109.5
C(18)-C(20)-H(20A)	109.5
C(18)-C(20)-H(20B)	109.5
H(20A)-C(20)-H(20B)	109.5
C(18)-C(20)-H(20C)	109.5
H(20A)-C(20)-H(20C)	109.5
H(20B)-C(20)-H(20C)	109.5
C(18)-C(21)-H(21A)	109.5
C(18)-C(21)-H(21B)	109.5
H(21A)-C(21)-H(21B)	109.5
C(18)-C(21)-H(21C)	109.5
H(21A)-C(21)-H(21C)	109.5
H(21B)-C(21)-H(21C)	109.5
C(17A)-C(14A)-C(15A)	111.7(18)
C(17A)-C(14A)-C(16A)	112.3(15)
C(15A)-C(14A)-C(16A)	103.2(17)
C(17A)-C(14A)-P(1)	108.9(13)
C(15A)-C(14A)-P(1)	108.1(13)
C(16A)-C(14A)-P(1)	112.5(13)
C(14A)-C(15A)-H(15A)	109.5
C(14A)-C(15A)-H(15B)	109.5
H(15A)-C(15A)-H(15B)	109.5
C(14A)-C(15A)-H(15C)	109.5
H(15A)-C(15A)-H(15C)	109.5
H(15B)-C(15A)-H(15C)	109.5
C(14A)-C(16A)-H(16A)	109.5
C(14A)-C(16A)-H(16B)	109.5
H(16A)-C(16A)-H(16B)	109.5
C(14A)-C(16A)-H(16C)	109.5
H(16A)-C(16A)-H(16C)	109.5
H(16B)-C(16A)-H(16C)	109.5
C(14A)-C(17A)-H(17A)	109.5
C(14A)-C(17A)-H(17B)	109.5
H(17A)-C(17A)-H(17B)	109.5
C(14A)-C(17A)-H(17C)	109.5
H(17A)-C(17A)-H(17C)	109.5
H(17B)-C(17A)-H(17C)	109.5
C(23A)-C(22A)-C(24A)	112.5(16)
C(23A)-C(22A)-C(25A)	107.6(15)
C(24A)-C(22A)-C(25A)	107.1(12)
C(23A)-C(22A)-P(2)	113.3(14)
C(24A)-C(22A)-P(2)	123.8(13)
C(25A)-C(22A)-P(2)	88.2(10)
C(22A)-C(23A)-H(23A)	109.5
C(22A)-C(23A)-H(23B)	109.5
H(23A)-C(23A)-H(23B)	109.5
C(22A)-C(23A)-H(23C)	109.5
H(23A)-C(23A)-H(23C)	109.5
H(23B)-C(23A)-H(23C)	109.5
C(22A)-C(24A)-H(24A)	109.5
C(22A)-C(24A)-H(24B)	109.5
H(24A)-C(24A)-H(24B)	109.5
C(22A)-C(24A)-H(24C)	109.5
H(24A)-C(24A)-H(24C)	109.5
H(24B)-C(24A)-H(24C)	109.5
C(22A)-C(25A)-Ir(1)	109.0(10)
C(22A)-C(25A)-H(25A)	109.9
Ir(1)-C(25A)-H(25A)	109.9
C(22A)-C(25A)-H(25B)	109.9
Ir(1)-C(25A)-H(25B)	109.9
H(25A)-C(25A)-H(25B)	108.3
C(17B)-C(14B)-C(15B)	112.3(15)
C(17B)-C(14B)-C(16B)	106.3(14)
C(15B)-C(14B)-C(16B)	97.1(13)
C(17B)-C(14B)-P(2)	116.9(13)
C(15B)-C(14B)-P(2)	115.9(11)
C(16B)-C(14B)-P(2)	105.4(10)
C(14B)-C(15B)-H(15D)	109.5
C(14B)-C(15B)-H(15E)	109.5
H(15D)-C(15B)-H(15E)	109.5
C(14B)-C(15B)-H(15F)	109.5

H(15D)-C(15B)-H(15F)	109.5
H(15E)-C(15B)-H(15F)	109.5
C(14B)-C(16B)-H(16D)	109.5
C(14B)-C(16B)-H(16E)	109.5
H(16D)-C(16B)-H(16E)	109.5
C(14B)-C(16B)-H(16F)	109.5
H(16D)-C(16B)-H(16F)	109.5
H(16E)-C(16B)-H(16F)	109.5
C(14B)-C(17B)-H(17D)	109.5
C(14B)-C(17B)-H(17E)	109.5
H(17D)-C(17B)-H(17E)	109.5
C(14B)-C(17B)-H(17F)	109.5
H(17D)-C(17B)-H(17F)	109.5
H(17E)-C(17B)-H(17F)	109.5
C(23B)-C(22B)-C(24B)	113.4(18)
C(23B)-C(22B)-C(25B)	108(2)
C(24B)-C(22B)-C(25B)	108.2(14)
C(23B)-C(22B)-P(1)	118.5(15)
C(24B)-C(22B)-P(1)	115.5(14)
C(25B)-C(22B)-P(1)	89.9(10)
C(22B)-C(23B)-H(23D)	109.5
C(22B)-C(23B)-H(23E)	109.5
H(23D)-C(23B)-H(23E)	109.5
C(22B)-C(23B)-H(23F)	109.5
H(23D)-C(23B)-H(23F)	109.5
H(23E)-C(23B)-H(23F)	109.5
C(22B)-C(24B)-H(24D)	109.5
C(22B)-C(24B)-H(24E)	109.5
H(24D)-C(24B)-H(24E)	109.5
C(22B)-C(24B)-H(24F)	109.5
H(24D)-C(24B)-H(24F)	109.5
H(24E)-C(24B)-H(24F)	109.5
C(22B)-C(25B)-Ir(1)	106.8(10)
C(22B)-C(25B)-H(25C)	110.4
Ir(1)-C(25B)-H(25C)	110.4
C(22B)-C(25B)-H(25D)	110.4
Ir(1)-C(25B)-H(25D)	110.4
H(25C)-C(25B)-H(25D)	108.6

Table S18. Anisotropic displacement parameters ($Å^2 x 10^3$)

The anisotropic displacement factor exponent takes the form: $-2\pi^2[h^2 a^{*2}U^{11} + ... + 2 h k a^* b^* U^{12}]$

	U^{11}	U^{22}	U^{33}	U^{23}	U^{13}	U^{12}
Ir(1)	29(1)	17(1)	27(1)	-4(1)	10(1)	-4(1)
Cl(1)	55(1)	23(1)	48(1)	-9(1)	28(1)	1(1)
P(1)	35(1)	17(1)	31(1)	3(1)	12(1)	1(1)
P(2)	36(1)	23(1)	22(1)	-3(1)	8(1)	-7(1)
N(1)	41(3)	22(2)	39(3)	-6(2)	8(2)	2(2)
C(1)	32(3)	18(2)	29(3)	0(2)	7(2)	-6(2)
C(2)	40(3)	21(2)	31(3)	-1(2)	12(2)	-2(2)
C(3)	33(3)	28(3)	36(3)	-2(2)	11(2)	4(2)
C(4)	41(3)	18(2)	37(3)	-1(2)	8(2)	-1(2)
C(5)	44(3)	20(2)	29(3)	0(2)	10(2)	-5(2)
C(6)	46(4)	31(3)	28(3)	4(2)	18(3)	3(2)
C(7)	60(4)	26(3)	31(3)	4(2)	17(3)	4(3)
C(8)	40(4)	31(3)	39(3)	-5(2)	12(3)	8(3)
C(9)	51(4)	20(2)	52(4)	3(2)	3(3)	2(2)
C(10)	45(4)	34(3)	38(3)	-4(2)	8(3)	8(2)
C(11)	72(5)	47(4)	38(3)	0(3)	2(3)	31(3)
C(12)	86(6)	51(4)	27(3)	4(3)	4(3)	17(4)
C(13)	36(4)	63(4)	75(5)	-19(4)	-4(4)	9(3)
C(18)	61(4)	31(3)	24(3)	1(2)	2(3)	-3(3)
C(19)	145(8)	59(5)	37(4)	-14(3)	5(5)	34(5)
C(20)	137(8)	45(4)	28(3)	10(3)	11(4)	0(4)
C(21)	94(6)	114(7)	50(5)	6(5)	-33(5)	-46(6)
C(14A)	33(5)	39(4)	63(5)	-10(4)	6(4)	-3(4)
C(15A)	58(5)	33(4)	116(7)	-21(4)	22(5)	-17(3)
C(16A)	42(8)	75(9)	69(9)	18(7)	17(7)	-26(7)
C(17A)	95(10)	46(7)	74(9)	21(7)	-38(8)	-34(7)
C(22A)	49(4)	57(4)	63(6)	-6(5)	16(5)	5(4)
C(23A)	48(5)	96(10)	96(8)	23(7)	28(6)	10(7)

C(24A)	64(9)	93(8)	48(8)	37(7)	12(7)	-1(7)
C(25A)	57(8)	70(8)	59(7)	4(6)	-7(6)	-13(7)
C(14B)	49(4)	57(4)	63(6)	-6(5)	16(5)	5(4)
C(15B)	48(5)	96(10)	96(8)	23(7)	28(6)	10(7)
C(16B)	79(9)	45(7)	54(7)	4(6)	-23(7)	-23(7)
C(17B)	47(8)	65(8)	90(10)	26(8)	1(8)	-14(7)
C(22B)	33(5)	39(4)	63(5)	-10(4)	6(4)	-3(4)
C(23B)	58(5)	33(4)	116(7)	-21(4)	22(5)	-17(3)
C(24B)	38(6)	57(8)	81(10)	-10(7)	8(7)	-8(6)
C(25B)	28(5)	37(5)	49(6)	-8(5)	-4(5)	-15(4)

Table S19. Hydrogen coordinates (x 10^4) and isotropic displacement parameters (Å^{2}x 10^3)

	x	y	z	U(eq)
H(6A)	4707	6536	4892	42
H(6B)	6522	6925	5055	42
H(7A)	5485	5197	7532	47
H(7B)	3918	4655	7265	47
H(8A)	8430	5745	4897	55
H(8B)	6804	5338	4570	55
H(8C)	8206	4706	4857	55
H(9A)	5425	3487	6868	62
H(9B)	7153	3882	7123	62
H(9C)	7112	3306	6517	62
H(11A)	1576	9264	5004	78
H(11B)	3286	9462	5368	78
H(11C)	1848	8912	5683	78
H(12A)	3289	8682	4190	82
H(12B)	4387	7817	4289	82
H(12C)	5023	8732	4563	82
H(13A)	870	7808	4661	87
H(13B)	1194	7380	5316	87
H(13C)	2142	7007	4740	87
H(19A)	2999	7488	8695	121
H(19B)	1419	7026	8373	121
H(19C)	2547	7686	7997	121
H(20A)	3628	5963	8992	105
H(20B)	4271	5291	8493	105
H(20C)	2322	5498	8536	105
H(21A)	5723	7043	8555	130
H(21B)	5569	7288	7851	130
H(21C)	6186	6334	8053	130
H(15A)	6541	9427	6487	103
H(15B)	4665	9153	6311	103
H(15C)	5755	9684	5837	103

H(16A)	8625	8801	5477	93
H(16B)	6968	9154	5140	93
H(16C)	7562	8166	5038	93
H(17A)	8410	8192	6432	109
H(17B)	7721	7296	6146	109
H(17C)	6743	7761	6681	109
H(23A)	-1146	6159	6988	119
H(23B)	58	6664	7460	119
H(23C)	-599	5703	7615	119
H(24A)	-197	4734	6585	102
H(24B)	820	4399	7174	102
H(24C)	1775	4586	6559	102
H(25A)	548	6704	6301	75
H(25B)	1619	5909	6038	75
H(15D)	-1497	6356	7243	119
H(15E)	-65	7074	7349	119
H(15F)	-561	6435	7890	119
H(16D)	-695	5902	6444	89
H(16E)	925	5328	6312	89
H(16F)	1060	6372	6346	89
H(17D)	-652	4835	7351	101
H(17E)	847	4854	7843	101
H(17F)	1172	4522	7168	101
H(23D)	6842	9396	6339	103
H(23E)	4869	9240	6328	103
H(23F)	5795	9465	5714	103
H(24D)	8707	8404	6031	88
H(24E)	7990	8169	5367	88
H(24F)	8131	7425	5876	88
H(25C)	5694	8152	6985	46
H(25D)	6757	7334	6748	46

Table S20. Torsion angles [°]

C(5)-C(1)-C(2)-C(3)	-2.4(8)	C(14A)-P(1)-C(10)-C(12)	61.1(8)
Ir(1)-C(1)-C(2)-C(3)	178.9(4)	Ir(1)-P(1)-C(10)-C(12)	-169.4(4)
C(5)-C(1)-C(2)-C(6)	174.8(5)	C(22B)-P(1)-C(10)-C(11)	-52.8(8)
Ir(1)-C(1)-C(2)-C(6)	-3.9(8)	C(6)-P(1)-C(10)-C(11)	-173.1(5)
C(4)-N(1)-C(3)-C(2)	-1.0(9)	C(14A)-P(1)-C(10)-C(11)	-62.5(8)
C(4)-N(1)-C(3)-C(8)	178.8(5)	Ir(1)-P(1)-C(10)-C(11)	67.0(5)
C(1)-C(2)-C(3)-N(1)	3.0(9)	C(22B)-P(1)-C(10)-C(13)	-169.5(7)
C(6)-C(2)-C(3)-N(1)	-174.2(5)	C(6)-P(1)-C(10)-C(13)	70.1(5)
C(1)-C(2)-C(3)-C(8)	-176.7(5)	C(14A)-P(1)-C(10)-C(13)	-179.2(7)
C(6)-C(2)-C(3)-C(8)	6.1(9)	Ir(1)-P(1)-C(10)-C(13)	-49.8(5)
C(3)-N(1)-C(4)-C(5)	-1.6(9)	C(7)-P(2)-C(18)-C(19)	-176.8(6)
C(3)-N(1)-C(4)-C(9)	-179.6(5)	C(22A)-P(2)-C(18)-C(19)	-60.5(9)
N(1)-C(4)-C(5)-C(1)	2.1(9)	C(14B)-P(2)-C(18)-C(19)	-59.7(8)
C(9)-C(4)-C(5)-C(1)	-180.0(5)	Ir(1)-P(2)-C(18)-C(19)	63.5(6)
N(1)-C(4)-C(5)-C(7)	-176.6(6)	C(7)-P(2)-C(18)-C(20)	-52.7(6)
C(9)-C(4)-C(5)-C(7)	1.3(9)	C(22A)-P(2)-C(18)-C(20)	63.6(9)
C(2)-C(1)-C(5)-C(4)	0.0(8)	C(14B)-P(2)-C(18)-C(20)	64.4(8)
Ir(1)-C(1)-C(5)-C(4)	178.7(4)	Ir(1)-P(2)-C(18)-C(20)	-172.4(5)
C(2)-C(1)-C(5)-C(7)	178.7(5)	C(7)-P(2)-C(18)-C(21)	66.3(5)
Ir(1)-C(1)-C(5)-C(7)	-2.5(8)	C(22A)-P(2)-C(18)-C(21)	-177.5(8)
C(1)-C(2)-C(6)-P(1)	13.2(7)	C(14B)-P(2)-C(18)-C(21)	-176.7(7)
C(3)-C(2)-C(6)-P(1)	-169.6(5)	Ir(1)-P(2)-C(18)-C(21)	-53.4(6)
C(22B)-P(1)-C(6)-C(2)	83.6(7)	C(23A)-C(22A)-C(25A)-Ir(1)	109.7(15)
C(10)-P(1)-C(6)-C(2)	-148.7(4)	C(24A)-C(22A)-C(25A)-Ir(1)	-129.2(12)
C(14A)-P(1)-C(6)-C(2)	92.6(7)	P(2)-C(22A)-C(25A)-Ir(1)	-4.2(10)
Ir(1)-P(1)-C(6)-C(2)	-14.8(5)	C(6)-P(1)-C(22B)-C(23B)	152(2)
C(4)-C(5)-C(7)-P(2)	-173.5(5)	C(10)-P(1)-C(22B)-C(23B)	33(2)
C(1)-C(5)-C(7)-P(2)	7.8(7)	Ir(1)-P(1)-C(22B)-C(23B)	-102(2)
C(18)-P(2)-C(7)-C(5)	-140.5(4)	C(6)-P(1)-C(22B)-C(24B)	12.5(14)
C(22A)-P(2)-C(7)-C(5)	87.2(6)	C(10)-P(1)-C(22B)-C(24B)	-106.2(13)
C(14B)-P(2)-C(7)-C(5)	102.0(7)	Ir(1)-P(1)-C(22B)-C(24B)	118.4(12)
Ir(1)-P(2)-C(7)-C(5)	-8.6(5)	C(6)-P(1)-C(22B)-C(25B)	-97.7(8)
C(22B)-P(1)-C(10)-C(12)	70.8(8)	C(10)-P(1)-C(22B)-C(25B)	143.5(7)
C(6)-P(1)-C(10)-C(12)	-49.5(5)	Ir(1)-P(1)-C(22B)-C(25B)	8.1(9)

C(23B)-C(22B)-C(25B)-Ir(1)	110.5(15)	P(1)-C(22B)-C(25B)-Ir(1)	-9.5(10)
C(24B)-C(22B)-C(25B)-Ir(1)	-126.4(13)		

Table S21. Hydrogen bonds

D-H...A	d(D-H)	d(H...A)	d(D...A)	<(DHA)
C(6)-H(6A)...N(1)#1	0.99	2.67	3.611(8)	158.5
C(11)-H(11C)...Cl(1)	0.98	2.76	3.642(7)	149.5
C(19)-H(19C)...Cl(1)	0.98	2.70	3.606(8)	154.0
C(15B^b)-H(15E^b)...Cl(1)	0.98	2.73	3.64(2)	154.7

Symmetry transformations used to generate equivalent atoms:

#1 -x+1,-y+1,-z+1
